# An Artificial [Fe_4_S_4_]-Containing Metalloenzyme for the Reduction of CO_2_ to Hydrocarbons

**DOI:** 10.1021/jacs.3c03546

**Published:** 2023-06-30

**Authors:** Valerie Waser, Manjistha Mukherjee, Ryo Tachibana, Nico V. Igareta, Thomas R. Ward

**Affiliations:** † Department of Chemistry, University of Basel, BPR 1096, Mattenstrasse 22, 4058 Basel, Switzerland

## Abstract

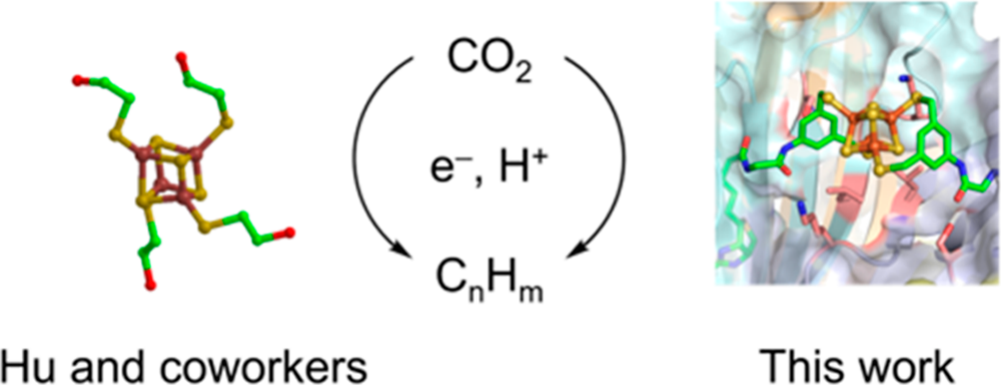

Iron–sulfur clusters have been reported to catalyze various redox transformations, including the multielectron reduction of CO_2_ to hydrocarbons. Herein, we report the design and assembly of an artificial [Fe_4_S_4_]-containing Fischer–Tropschase relying on the biotin–streptavidin technology. For this purpose, we synthesized a bis-biotinylated [Fe_4_S_4_] cofactor with marked aqueous stability and incorporated it in streptavidin. The effect of the second coordination sphere provided by the protein environment was scrutinized by cyclic voltammetry, highlighting the accessibility of the doubly reduced [Fe_4_S_4_] cluster. The Fischer–Tropschase activity was improved by chemo-genetic means for the reduction of CO_2_ to hydrocarbons with up to 14 turnovers.

## 1 Introduction

Iron–sulfur metallocofactors are ubiquitous in nature and involved in some of the earth’s most fundamental biological processes. Among them, the cuboidal [Fe_4_S_4_] cluster is the most common representative and is widely known for its role in mediating electron transfer. However, the catalytic function of [Fe_4_S_4_] is increasingly recognized.^1−3^ While electron transport chains rely on the redox couples [Fe_4_S_4_]^3+/2+^ and [Fe_4_S_4_]^2+/1+^, the oxidation state [Fe_4_S_4_]^0^ is particularly intriguing for catalytic purposes, as such highly reduced species are capable of activating very inert moieties. For instance, it was reported recently that a radical *S*-adenosylmethionine (SAM) enzyme of *Methanocaldococcus jannaschii* catalyzes the coupling of two lipid chains.^4^ Thereby, an [Fe_4_S_4_]^0^ cluster activates two sp^3^-carbon centers, ultimately leading to the formation of a C–C bond. Further, Suess and co-workers have investigated the electronic configuration of a synthetic [Fe_4_S_4_]^0^ cluster supported by N-heterocyclic carbene ligands.^5^ The binding of a CO ligand to the FeS core induces an intramolecular valence disproportionation, and the CO-bound Fe site adopts a low-valent Fe^1+^ oxidation state. Thereby, the C–O bond exhibits remarkable activation, as evidenced by spectroscopy. However, elucidating the fascinating properties of [Fe_4_S_4_]^0^ clusters is challenged by their pronounced reactivity. Although all-thiolate ligated [Fe_4_S_4_]^0^ clusters have been observed electrochemically since the 1970s, isolating such clusters has not been realized until recently.^6,7^

The aforementioned lipid-modifying SAM enzyme, as well as Suess’ CO-bound [Fe_4_S_4_]^0^ cluster, relies on a 3:1 site-differentiated [Fe_4_S_4_] cluster, in which the unique iron atom is coordinated to a labile ligand (i.e., histidine/Cl^–^) before being replaced by the substrate/CO ligand. The 3:1 site-differentiated pattern is found throughout several classes of catalytically active FeS proteins, including isoprenoid synthesis proteins (IspG and IspH), aconitase, (*R*)-2-hydroxyacyl-CoA dehydratase, and the superfamily of SAM enzymes.^8−11^ It has been postulated that these unsaturated forms may be essential for reactivity.^12^

The remarkable catalytic properties of FeS clusters are further highlighted by the work of the Ribbe and Hu groups. They reported on the propensity of [Fe_4_S_4_]-containing metalloproteins to catalyze the reduction of CO and CO_2_ to hydrocarbons (alka/enes hereafter).^13−16^ Strikingly, they showed that the reaction was also catalyzed by a synthetic [Fe_4_S_4_(SCH_2_CH_2_OH)_4_] cluster in the presence of strong reducing agents, in either aqueous or organic solvents.^17^ These results contrast with most catalytic systems, which rarely lead to the formation of multiple C–C bonds upon reduction of CO_2_.^18−20^

Inspired by these results, we speculated that embedding a biotinylated [Fe_4_S_4_] cluster into a protein environment may enable us to engineer and evolve an artificial metalloenzyme (ArM) for the reduction of CO_2_ to alka/enes (Fischer–Tropschase, FTase hereafter). Anchoring a metallocofactor into a protein scaffold provides a well-defined second coordination sphere around the cofactor, thus offering straightforward means of optimizing the catalytic performance by chemical and genetic methods.^21^ A scaffold of particular interest is streptavidin (Sav), thanks to its exceptionally high affinity for biotin.^22,23^ In the past 20 years, Sav has proven to be a privileged host for incorporating various biotinylated cofactors. The resulting ArMs were chemo-genetically optimized to catalyze various reactions including metathesis, C–H activation, hydroamination, hydrogenation, and hydrogen production.^24−31^ Other versatile host proteins that have been used for the generation of ArMs include carbonic anhydrase, hemoproteins, prolyl oligopeptidase, helical bundles, the lactococcal multiresistance regulator, and *de novo* designed metallopeptides.^32−39^ [Fe_4_S_4_] clusters have been incorporated into thioredoxin, cytochrome *c* peroxidase, and several *de novo* structures.^40−46^ Herein, we report on our efforts to optimize the catalytic performance of an [Fe_4_S_4_]-containing FTase based on the biotin–streptavidin technology.

## 2 Results and Discussion

### 2.1 Cofactor Design

With the aim of minimizing the decomposition of the [Fe_4_S_4_] core, we selected a 3,5-bis(mercaptomethyl)benzene scaffold, which has been shown by Holm and co-workers to bind to two adjacent Fe centers in [Fe_4_S_4_] tightly.^6,47^ We speculated that an incoming substrate might displace one of the thiols of the bidentate ligand. However, as the thiol remains in proximity of the Fe center during and after substrate turnover, the decomposition of the cluster by aquation may be minimized.

**Figure 1:**
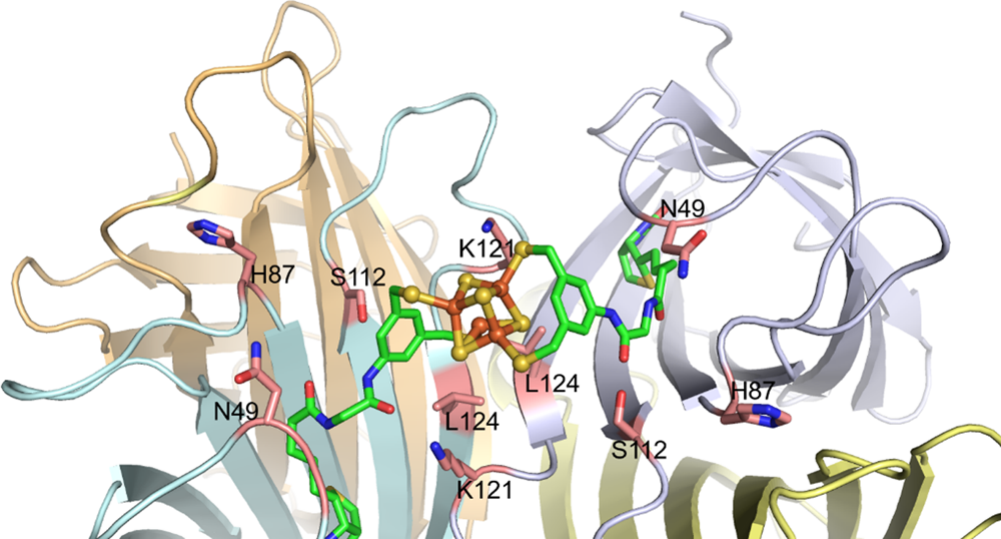
Close-up view of the calculated structure of **[(Biot-gly)**_**2**_**Fe**_**4**_**S**_**4**_**]**·Sav WT (color code cofactor: C: green, O: red, N: blue, Fe: orange, and S: yellow, the close-lying residues N49, H87, S112, K121, and L124 are highlighted as sticks: C: tan).

The homotetrameric structure of Sav can be described as a dimer-of-dimers, with each dimer consisting of two biotin-binding sites facing each other. Capitalizing on this feature, we hypothesized that it might be possible to coordinate the [Fe_4_S_4_] cluster with two biotinylated 3,5-bis(mercaptomethyl)benzene ligands—and thus four thiolate donors—to firmly anchor the cofactor within the biotin-binding vestibule. Relying on QM-MM calculations, we selected glycine as a spacer to enforce the coordination of the cofactor to two adjacent biotin-binding sites and thus minimize the cross-linking of Sav to afford oligomers. The modeled structure of **[(Biot-gly)**_**2**_**Fe**_**4**_**S**_**4**_**]**·Sav WT is displayed in Figure 1. Computational details are collected in the Supporting Information.

### 2.2 Synthesis of **[(Biot-gly)_2_Fe_4_S_4_]**

**Scheme 1:**
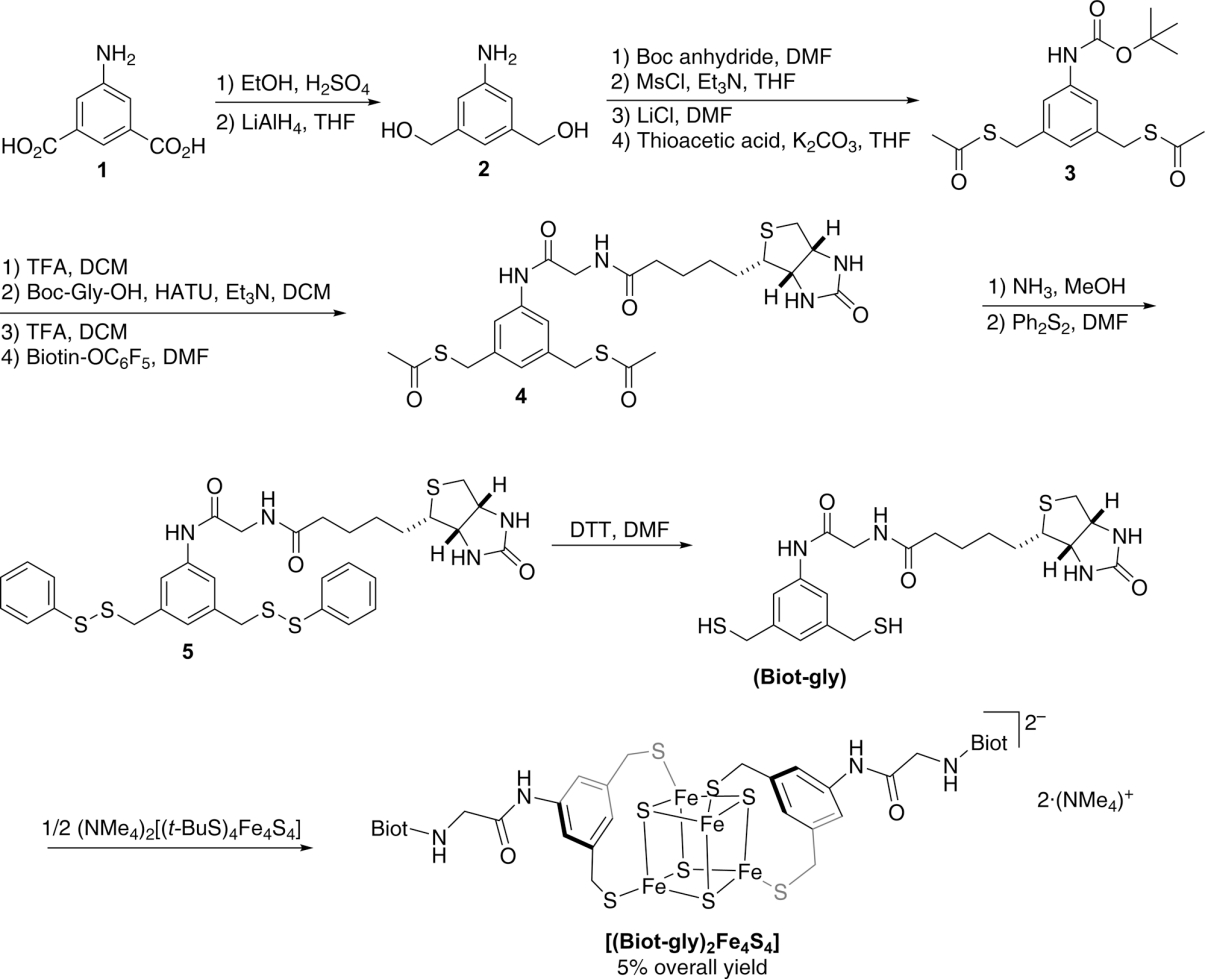
Thirteen-Step Synthesis of the Biotinylated **(Biot-gly)** and Ligand Exchange with [(*t*-BuS)_4_Fe_4_S_4_]^2–^ to Afford the Corresponding Bis-Biotinylated Cluster **[(Biot-gly)**_**2**_**Fe**_**4**_**S**_**4**_**]**

We set out to synthesize a biotinylated ligand bearing a glycine spacer between the biotin anchor and the 3,5-bis(mercaptomethyl)aniline, Scheme 1. Esterification of the *m-*dicarboxylate **1** was followed by LiAlH_4_ reduction to afford the corresponding diol **2**. Next, *N-*Boc protection of the aniline and nucleophilic substitution of the benzylic alcohol yielded the bis(thioester) **3**. After N-Boc deprotection, the glycine spacer was introduced using HATU as a coupling agent. Finally, biotin was introduced using biotin pentafluorophenyl ester to afford the bis(thioester) **4**. For purification purposes, the bis(thioester) **4** was converted into the corresponding bis(disulfide) **5**, which was isolated in analytically pure form following silica gel chromatography. The addition of excess dithiothreitol yielded the desired ligand **(Biot-gly)**. Having purified and fully characterized the **(Biot-gly)** by NMR and HRMS, it was reacted with half an equivalent of [(*t*-BuS)_4_Fe_4_S_4_]^2–^ to afford the corresponding bis-biotinylated cluster **[(Biot-gly)**_**2**_**Fe**^**II**^_**2**_**Fe**^**III**^_**2**_**S**_**4**_**]**^**2–**^ and four equivalents of *t*-BuSH, which were removed *in vacuo*.^48^ The cluster was characterized by UV–vis, HRMS, and paramagnetic ^1^H NMR, to confirm the purity of the oxygen-sensitive bis-biotinylated cluster **[(Biot-gly)**_**2**_**Fe**_**4**_**S**_**4**_**]**; see the Supporting Information for experimental details.

### 2.3 Aqueous Stability of **[(Biot-gly)_2_Fe_4_S_4_]**

**Figure 2:**
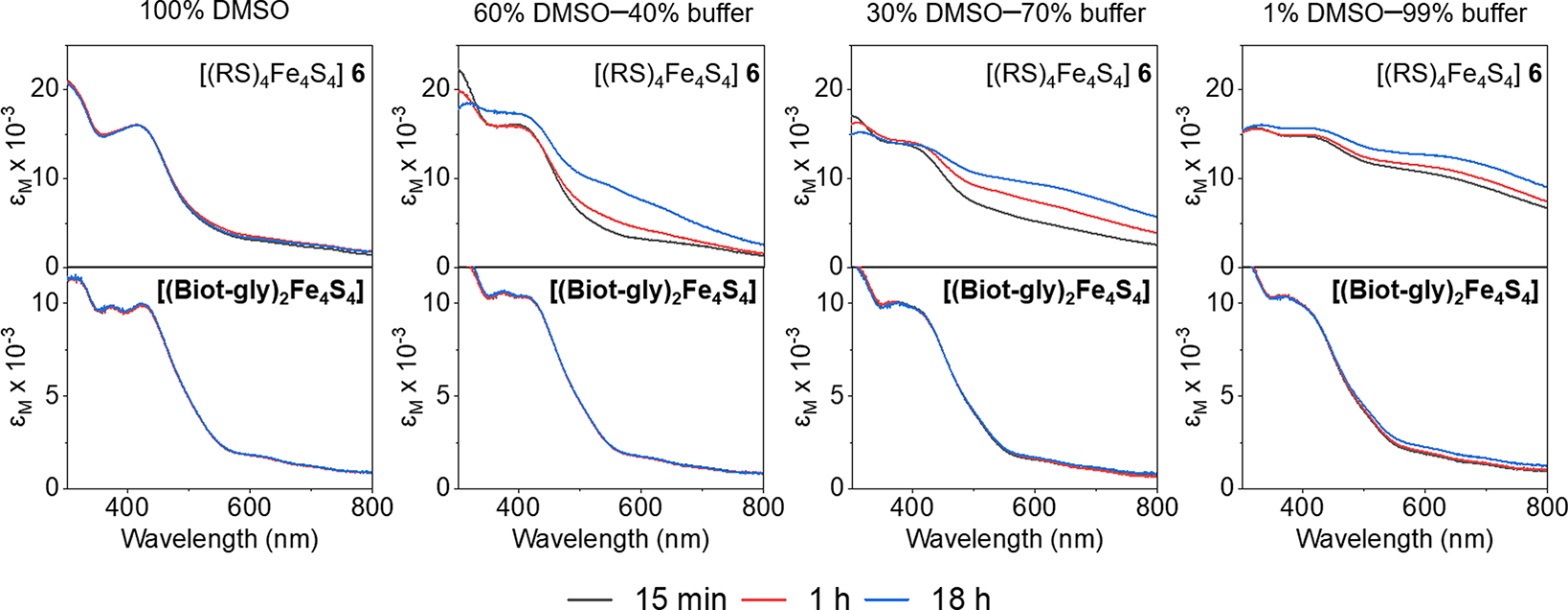
Stability of **[(Biot-gly)**_**2**_**Fe**_**4**_**S**_**4**_**]** and cluster [(RS)_4_Fe_4_S_4_] **6** in aqueous solutions. The UV–vis spectra were recorded in DMSO–borate buffer (pH 8.2, 0.2 M) mixtures at different time points.

Typically, [(RS)_4_Fe_4_S_4_] clusters display limited stability toward water.^49,50^ As the thiolate ligands are prone to substitution reactions with coordinating solvents, the addition of excess ligand stabilizes the FeS cores under basic conditions.^51−54^ Further, the stability can be improved by using large hydrophilic ligands or adding surfactants.^47,50,55,56^ However, in the absence of excess ligand or surfactant, the reported [(RS)_4_Fe_4_S_4_] clusters are only stable up to a water content of around 40%. We hypothesized that the chelating nature of the dithiolate ligand **(Biot-gly)** might minimize aquation, thus increasing the stability of **[(Biot-gly)**_**2**_**Fe**_**4**_**S**_**4**_**]** in water. Inspired by a publication by Holm and co-workers, we examined the aqueous stability of **[(Biot-gly)**_**2**_**Fe**_**4**_**S**_**4**_**]** spectrophotometrically.^50^ For comparison, the same experiment was conducted with (NMe_4_)_2_[(HOCH_2_CH_2_S)_4_Fe_4_S_4_] (**6**), which previously had been reported to be stable in partially aqueous solutions.^50^ Accordingly, the two clusters were dissolved in mixtures of DMSO and borate buffer (pH 8.2, 0.2 M), and the spectral changes were monitored over time, Figure 2. As reported, the spectrum of the model cluster [(RS)_4_Fe_4_S_4_] **6** in DMSO remained unchanged for >18 h. However, in the presence of 40% borate buffer, the spectrum already exhibits changes after 1 h. After 18 h, a significantly elevated baseline and decreased features at 400 nm are observed, indicative of cluster decomposition.^47^ At 99% aqueous content, the spectrum is nearly featureless after 15 min. Gratifyingly, **[(Biot-gly)**_**2**_**Fe**_**4**_**S**_**4**_**]** proved remarkably stable even in 99% borate buffer: no notable spectral change was apparent for >18 h. To the best of our knowledge, **[(Biot-gly)**_**2**_**Fe**_**4**_**S**_**4**_**]** represents the first synthetic [Fe_4_S_4_] cluster that is stable in an aqueous solution in the absence of excess ligand.^49^

### 2.4 Characterization of **[(Biot-gly)_2_Fe_4_S_4_]**·Sav

**Figure 3:**
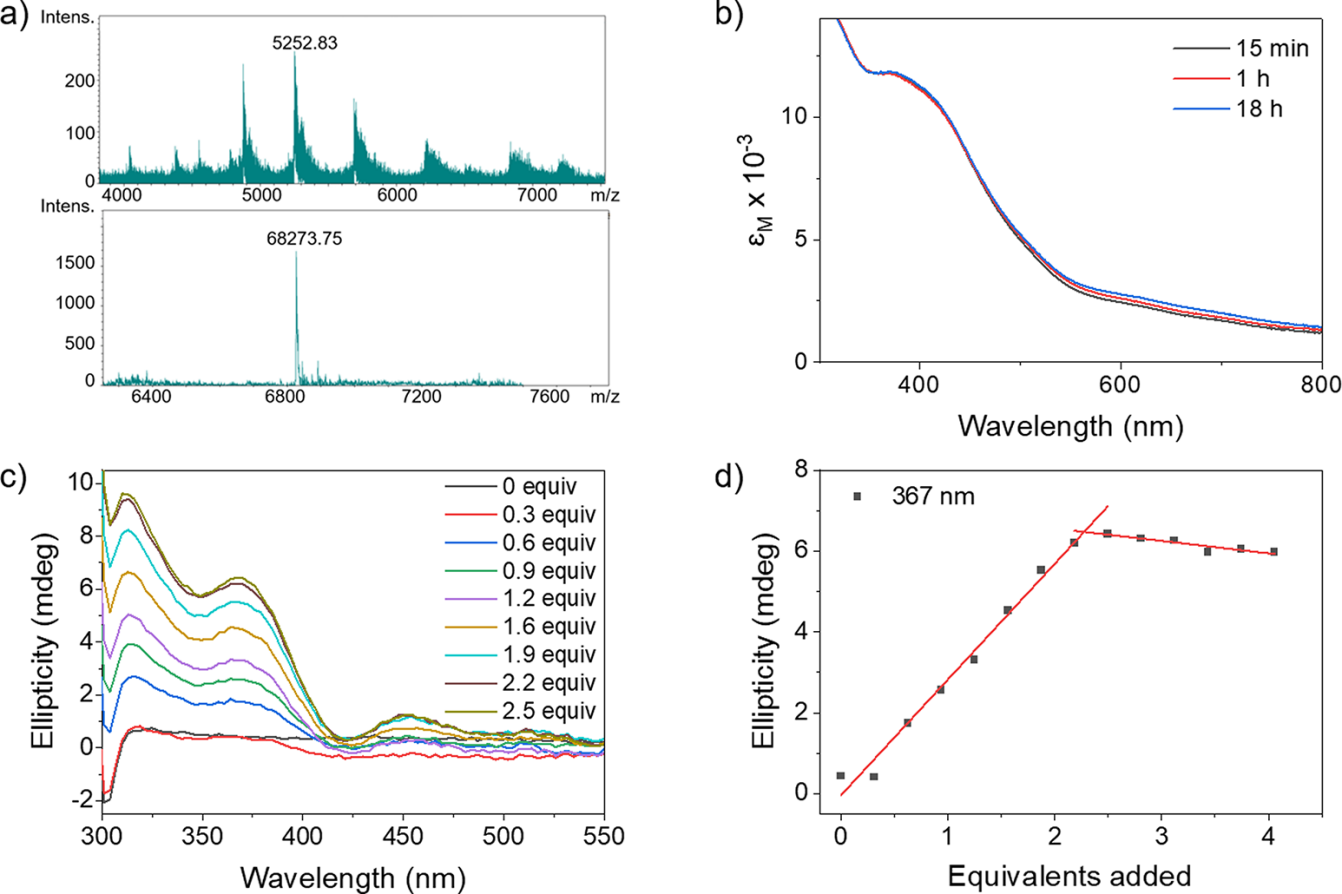
Structural characterization of **[(Biot-gly)**_**2**_**Fe**_**4**_**S**_**4**_**]**·Sav. (a) Native mass spectrum of **[(Biot-gly)**_**2**_**Fe**_**4**_**S**_**4**_**]**_2_·Sav WT: charge-state envelope (top) and deconvoluted (i.e., summed zero-charge mass distribution, bottom) mass spectrum resulting from incubating 2 equiv of **[(Biot-gly)**_**2**_**Fe**_**4**_**S**_**4**_**]** with 1 equiv of homotetrameric Sav (calculated for **[(Biot-gly)**_**2**_**Fe**_**4**_**S**_**4**_**]**_2_·Sav WT: 68273.6 *m*/*z*, found: 68273.8 *m*/*z*). (b) UV–vis spectra of **[(Biot-gly)**_**2**_**Fe**_**4**_**S**_**4**_**]**·Sav AA in a 1% DMSO–99% borate buffer (pH 8.2, 0.2 M) mixture at different time points highlighting a stability for >18 h. (c) CD titration of Sav AA with **[(Biot-gly)**_**2**_**Fe**_**4**_**S**_**4**_**]** revealing the appearance of three CD bands (λ_max_ = 316 nm, λ_max_ = 376 nm, and λ_max_ = 457 nm). (d) Monitoring of the resulting molar ellipticity at 367 nm CD highlighting the linear segment profile of the titration with an equivalence point reached at 2.3 equiv, thus confirming the 2:1 stoichiometry 2 **[(Biot-gly)**_**2**_**Fe**_**4**_**S**_**4**_**]**:homotetrameric Sav.

Next, we set out to investigate the incorporation of **[(Biot-gly)**_**2**_**Fe**_**4**_**S**_**4**_**]** within Sav. Both circular dichroism spectroscopy (CD) and native mass spectroscopy (HRMS) unambiguously highlight the formation of discrete **[(Biot-gly)**_**2**_**Fe**_**4**_**S**_**4**_**]**_**2**_·Sav intramolecular assemblies (i.e., two clusters per homotetrameric Sav), rather than the formation of cross-linked, oligomeric **[(Biot-gly)**_**2**_**Fe**_**4**_**S**_**4**_**]**_*n*_·Sav_*m*_ assemblies. As can be appreciated, anchoring of two **[(Biot-gly)**_**2**_**Fe**_**4**_**S**_**4**_**]** clusters within homotetrameric Sav WT is unambiguously confirmed by the presence of a peak at 68273.8 *m*/*z* in native HRMS experiments (**[(Biot-gly)**_**2**_**Fe**_**4**_**S**_**4**_**]**_**2**_·Sav WT calculated peak: 68273.6 *m*/*z*), Figure 3a. No significant peak at higher *m*/*z* was detected, thus supporting the hypothesis that the topology of **[(Biot-gly)**_**2**_**Fe**_**4**_**S**_**4**_**]** favors the incorporation of its two biotins in adjacent biotin-binding sites over the formation of cross-linked Sav aggregates **[(Biot-gly)**_**2**_**Fe**_**4**_**S**_**4**_**]**_*n*_·Sav_*m*_. Next, a CD titration was carried out whereby a Sav S112A K121A sample (Sav AA hereafter) was treated with increasing amounts of **[(Biot-gly)**_**2**_**Fe**_**4**_**S**_**4**_**]**. The gradual appearance of an induced CD signal in the absorbance window of **[(Biot-gly)**_**2**_**Fe**_**4**_**S**_**4**_**]** confirms that the achiral metal assembly experiences a well-defined chiral environment, thus highlighting its incorporation within Sav. Monitoring the increase in ellipticity at 367 nm reveals a linear increase up to 2.3 equivalents of **[(Biot-gly)**_**2**_**Fe**_**4**_**S**_**4**_**]** vs tetrameric Sav, thus confirming the incorporation of two equivalents of the bis-biotinylated cofactor **[(Biot-gly)**_**2**_**Fe**_**4**_**S**_**4**_**]** vs homotetrameric Sav, Figure 3c,d. Further, the aqueous stability of **[(Biot-gly)**_**2**_**Fe**_**4**_**S**_**4**_**]**_**2**_·Sav AA was examined in a solution containing 1% DMSO and 99% borate buffer (pH 8.2, 0.2 M); the spectrum exhibited only marginal changes over the course of 18 h, suggesting that the [Fe_4_S_4_] core remains intact, Figure 3b.

### 2.5 Cyclic Voltammetry

**Figure 4:**
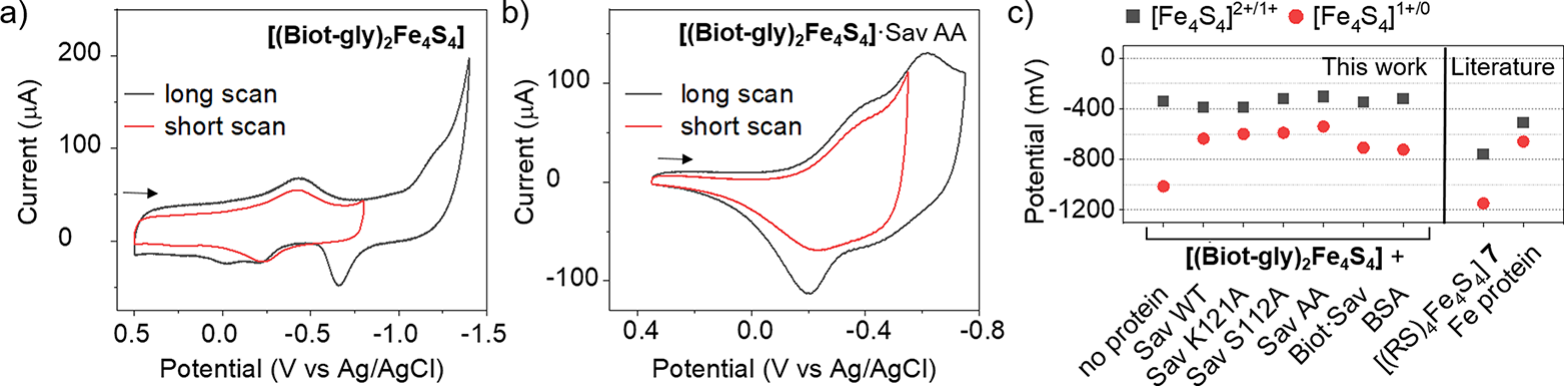
Effect of the protein environment on the redox potential of **[(Biot-gly)**_**2**_**Fe**_**4**_**S**_**4**_**]**·Sav. (a) Cyclic voltammogram of **[(Biot-gly)**_**2**_**Fe**_**4**_**S**_**4**_**]** (200 μM) in borate buffer (pH 8.2, 100 mM) (glassy carbon working electrode, scan rate 1 V·s^–1^). (b) Cyclic voltammogram of **[(Biot-gly)**_**2**_**Fe**_**4**_**S**_**4**_**]**·Sav AA in borate buffer (pH 8.2, 100 mM) absorbed on an l-cysteine-modified gold electrode (scan rate 1 V·s^–1^). (c) Measured redox potentials of various **[(Biot-gly)**_**2**_**Fe**_**4**_**S**_**4**_**]**·Sav mutants (see Figures S13, S18, S19) and reported redox potentials of the Fe protein of nitrogenase^64^ and cluster [(RS)_4_Fe_4_S_4_] **7**.^57^ All data were measured in triplicate; the standard deviation was ≤20 mV in all cases, Table S1.

The electrochemical properties of **[(Biot-gly)**_**2**_**Fe**_**4**_**S**_**4**_**]** and **[(Biot-gly)**_**2**_**Fe**_**4**_**S**_**4**_**]**·Sav were investigated by cyclic voltammetry (CV) in an aqueous borate buffer (pH 8.2, 100 mM) with KPF_6_ (100 mM) as a supporting electrolyte. The free cofactor **[(Biot-gly)**_**2**_**Fe**_**4**_**S**_**4**_**]** presents a reversible reduction wave with a half-wave potential (*E*_1/2_) of −343 mV (all potentials vs Ag/AgCl (saturated KCl)), Figure 4a. This redox process corresponds to the one-electron reduction of the [Fe_4_S_4_]^2+^ core. It is followed by a second irreversible reduction at −1014 mV presumably corresponding to the [Fe_4_S_4_]^1+/0^ couple, Figure 4a.^57^ The anodic scan after the second reduction reveals a nontrivial behavior: (i) a high current density at −649 mV is observed, and (ii) the anodic peak corresponding to the [Fe_4_S_4_]^1+/2+^ process splits into two peaks, indicative of some structural changes in the [Fe_4_S_4_] core. However, this does not affect the basic integrity of the cluster as derived from two consecutive CV scans, Figure S10. We hypothesize that a thiolate ligand dissociates from the [Fe_4_S_4_] core during the second reduction.^58^ The redox processes of **[(Biot-gly)**_**2**_**Fe**_**4**_**S**_**4**_**]**·Sav are diffusion-controlled. To circumvent this limitation, the FTase was adsorbed on an l-cysteine-modified gold electrode to scrutinize its redox behavior.^59^ The corresponding cyclic voltammograms reveal that the [Fe_4_S_4_]^2+/1+^ reduction potential of **[(Biot-gly)**_**2**_**Fe**_**4**_**S**_**4**_**]**·Sav shifts by +38 mV to −305 mV upon incorporation into Sav AA, Figure 4b. However, a much larger effect of the protein environment on the [Fe_4_S_4_]^1+/0^ redox couple is observed: the potential shifts by +500 mV to −514 mV, Figure 4b.

Both synthetic and biological clusters have been studied to identify the first- and second-coordination sphere factors that affect the redox potentials of [Fe_4_S_4_] clusters. The most commonly discussed factors include (i) the nature of the solvent and accessibility of the cluster to the solvent, (ii) hydrogen bonds and dipoles from backbone amides in proximity of the cluster, (iii) electrostatic effects, and (iv) the identity of the ligands.^60−63^ These factors dictate by-and-large which redox couple is operative in FeS proteins. A particular case is the Fe protein of nitrogenase, which is among the rare proteins that stabilize the [Fe_4_S_4_]^0^ state.^64−66^ Its ability to stabilize the highly reduced [Fe_4_S_4_]^0^ cluster has been hypothesized to arise from (i) the large number of H-bonds from the protein backbone to the sulfide groups of the [Fe_4_S_4_] core and the thiolate ligands, (ii) high solvent accessibility of the [Fe_4_S_4_] center, and (iii) dipoles arising from the amide backbone.^67,68^ In contrast, the stabilities of synthetic [Fe_4_S_4_]^0^ clusters in an aqueous environment have not been thoroughly investigated. An exception is the cluster [(COOHCH_2_CH_2_S)_4_Fe_4_S_4_] (**7**), whose redox potentials were determined in the presence of excess ligand, Figure 4c.^57^

Upon embedding the cofactor **[(Biot-gly)**_**2**_**Fe**_**4**_**S**_**4**_**]** into Sav AA, marked differences in the redox behavior are clearly apparent, Figure 4a,b. Inspired by the Fe protein of nitrogenase, we hypothesize that hydrogen bonds between the cluster and close-lying amino acid residues may stabilize the highly reactive [Fe_4_S_4_]^0^ species and shift the potentials of the [Fe_4_S_4_]^2+/1+^ and [Fe_4_S_4_]^1+/0^ redox events closer together. The anodic peak splitting, which was observed during the [Fe_4_S_4_]^1+/2+^ process for the free cofactor after the second reduction, disappeared for **[(Biot-gly)**_**2**_**Fe**_**4**_**S**_**4**_**]**·Sav AA. If operative, the ligand dissociation from the [Fe_4_S_4_] core is potentially minimized by the preorganization of the protein-confined ligands (**Biot-gly**), which may favor the rapid thiolate recoordination in the event of ligand (partial) dissociation.

As highlighted in Figure 1, residues 112 and 121 of Sav lie close to the four thiolate ligands of the [Fe_4_S_4_] core. To investigate the effect of these residues on the redox potential, cyclic voltammograms of four single mutants of **[(Biot-gly)**_**2**_**Fe**_**4**_**S**_**4**_**]**·Sav were recorded, Figure 4c. The [Fe_4_S_4_]^2+/1+^ redox couple appears to be affected by the S112A mutation (+68 mV), whereas the mutation K121A only had a marginal effect. On the other hand, the potential for the [Fe_4_S_4_]^1+/0^ couple ranges from −637 to −514 mV, whereby the mutations in both positions S112A and K121A resulted in substantial positive shifts. Thus, it appears that increased hydrophobicity around the thiolate ligands of the [Fe_4_S_4_]^2+^ core contributes to stabilizing both reduction processes, whereby the effect is more pronounced for the [Fe_4_S_4_]^1+/0^ redox process.

To test how nonspecific hydrophobic interactions influence the redox behavior of **[(Biot-gly)**_**2**_**Fe**_**4**_**S**_**4**_**]**, we measured its redox potentials in the presence of bovine serum albumin (BSA), Figure 4c. Likewise, the interaction between **[(Biot-gly)**_**2**_**Fe**_**4**_**S**_**4**_**]** and the surface of Sav AA was examined by saturating the protein with biotin prior to the addition of the cofactor (Biot·Sav, hereafter) to minimize the assembly of the ArM. The [Fe_4_S_4_]^1+/0^ couple was affected in both cases and shifted to more positive potentials. However, the measured potentials are still well below that of **[(Biot-gly)**_**2**_**Fe**_**4**_**S**_**4**_**]**·Sav. This suggests that incorporation of the cofactor in the biotin-binding vestibule significantly contributes to the stabilization of the [Fe_4_S_4_]^0^ core in **[(Biot-gly)**_**2**_**Fe**_**4**_**S**_**4**_**]**·Sav.

### 2.6 Fischer–Tropschase Activity

**Figure 5:**
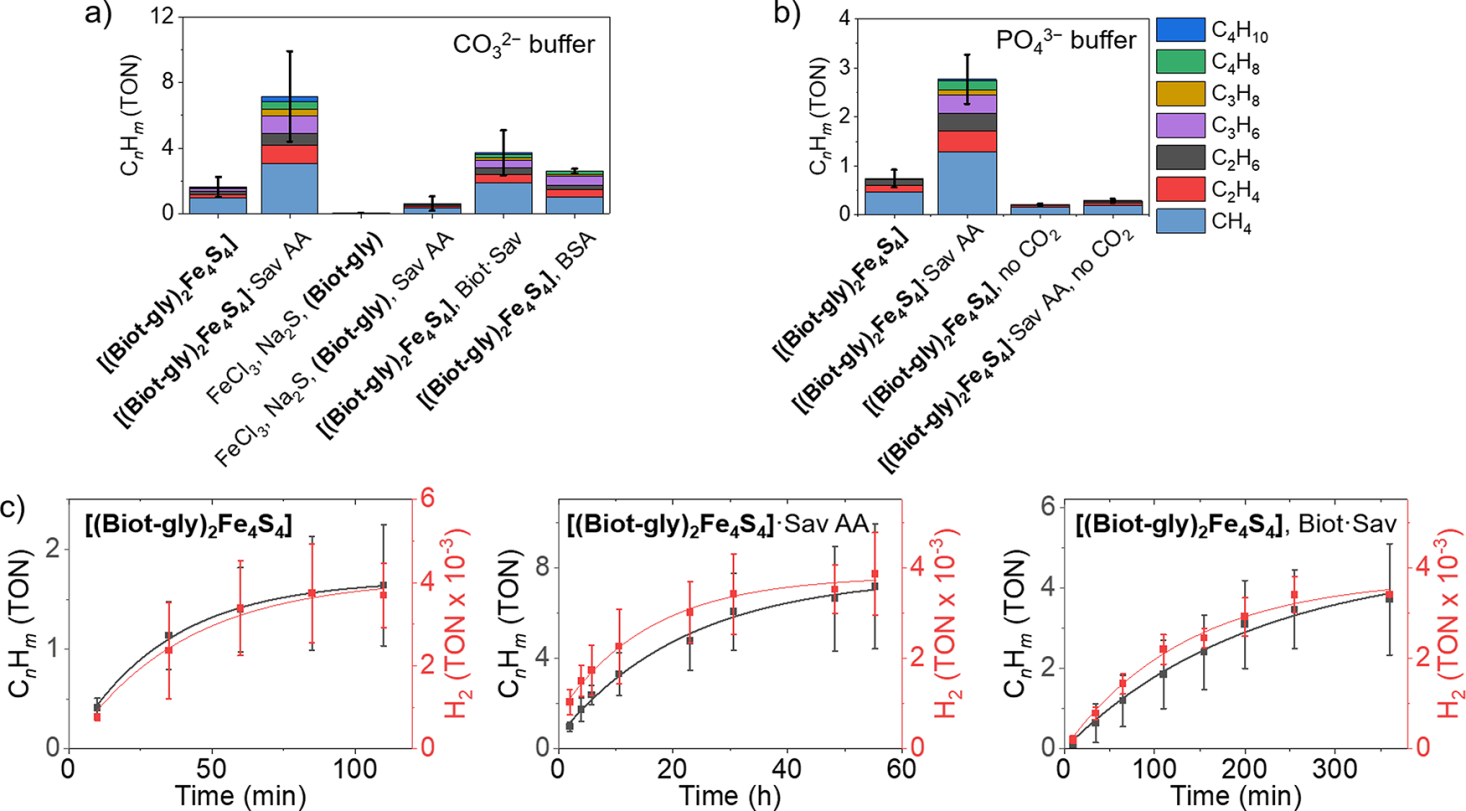
FTase activity of **[(Biot-gly)**_**2**_**Fe**_**4**_**S**_**4**_**]** and **[(Biot-gly)**_**2**_**Fe**_**4**_**S**_**4**_**]**·Sav AA and control experiments (all at 5 μM catalyst concentration ([Fe_4_S_4_] sites) (or an equimolar amount of FeCl_3_/Na_2_S/**(Biot-gly)**) and in the presence of 40 mM Eu(II)-DTPA). (a) CO_2_ reduction in a 100% CO_2_ atmosphere in carbonate buffer (pH 7.5) with **[(Biot-gly)**_**2**_**Fe**_**4**_**S**_**4**_**]** or equimolar amounts of FeCl_3_/Na_2_S/**(Biot-gly)** in the presence and absence of Sav AA, BSA, and Biot·Sav. (b) CO_2_ reduction in phosphate buffer (pH 7.5) in the presence and absence of CO_2_ (generated from NaHCO_3_). (c) Time-course of C_*n*_H_*m*_ production (alka/enes, black) and H_2_ evolution (red) during CO_2_ reduction with **[(Biot-gly)**_**2**_**Fe**_**4**_**S**_**4**_**]** in the presence and absence of Sav AA and Biot·Sav (with a first-order kinetics fit, Table S2). As the C_*n*_H_*m*_ and H_2_ production levels off, addition of Eu(II)-DTPA restores the FTase activity, Figure S21. The experiments were performed in triplicate with standard deviation displayed.

In the presence of CO_2_ (1 atm) and Eu(II)-DTPA (*E*^0^ = −1.3 V vs Ag/AgCl at pH 8)^69^ as reductant, **[(Biot-gly)**_**2**_**Fe**_**4**_**S**_**4**_**]**·Sav AA catalyzes the production of short alkanes and alkenes (C_1_–C_4_), which were detected by GC-FID and GC-MS. Compared to the free cofactor, the ArM displays improved turnover numbers (TONs), Figure 5a. The addition of equimolar amounts of FeCl_3_, Na_2_S, and **(Biot-gly)** instead of the assembled **[(Biot-gly)**_**2**_**Fe**_**4**_**S**_**4**_**]** led to minimal background activity. This strongly suggests that the FTase **[(Biot-gly)**_**2**_**Fe**_**4**_**S**_**4**_**]**·Sav AA is the catalyst precursor for the reduction of CO_2_ to alka/enes. Nonspecific hydrophobic interactions between **[(Biot-gly)**_**2**_**Fe**_**4**_**S**_**4**_**]** and BSA or Biot·Sav led to slightly increased FTase activity compared to the free cofactor. However, to achieve maximal TONs, embedding the cofactor in the biotin-binding pocket is essential.

In the absence of CO_2_, some residual FTase activity is detected, presumably due to the reduction of the cosolvent DMF, Figure 5b. Upon relying on ^13^CO_3_HNa as a CO_2_ source, the detection of ^13^C-alka/enes by GC-MS unambiguously confirms that dissolved CO_2_ (i.e., HCO_3_^–^) is indeed the major C-source of the alka/enes (100% for C_3_ and C_4_, 70% for C_2_H_6_, and 30% for C_2_H_4_), Figure S20.

A general challenge when performing CO_2_ reduction in water is the competing production of dihydrogen in the presence of strong reducing agents.^18^ The hydrogen evolution during CO_2_ reduction with **[(Biot-gly)**_**2**_**Fe**_**4**_**S**_**4**_**]** and **[(Biot-gly)**_**2**_**Fe**_**4**_**S**_**4**_**]**·Sav AA was monitored over time by GC-TCD, Figure 5c. Importantly, the kinetic behavior for both H_2_ and alka/enes follows similar trends: the corresponding first-order kinetic fit reveals no catalytic onset, neither for H_2_ nor for C_*n*_H_*m*_ production. The consumption of the reducing agent leads to leveling-off of C_*n*_H_*m*_ and H_2_ production after 2 h (**[(Biot-gly)**_**2**_**Fe**_**4**_**S**_**4**_**]**) and 48 h (**[(Biot-gly)**_**2**_**Fe**_**4**_**S**_**4**_**]**·Sav AA). However, the addition of Eu(II)-DTPA restores the FTase activity, Figure S21. This suggests that the nature of the active catalyst is by-and-large maintained beyond the indicated times. Further, **[(Biot-gly)**_**2**_**Fe**_**4**_**S**_**4**_**]**·Sav AA reacts much more slowly than the free **[(Biot-gly)**_**2**_**Fe**_**4**_**S**_**4**_**]** cofactor, despite overall higher TON. As observed in the cyclic voltammogram, the free cofactor exhibited structural changes (i.e., potential ligand dissociation) on the CV time scale. If such an event is essential for substrate binding, this might explain the increased CO_2_ fixation rate (albeit at the cost of a reduced TON). In the presence of Biot·Sav (1 equiv Sav AA and excess biotin added), the FTase activity is very similar to that of the free cofactor. This supports the hypothesis that the catalytically active species is indeed embedded in Sav during catalysis.

### 2.7 Chemo-genetic Optimization

**Figure 6:**
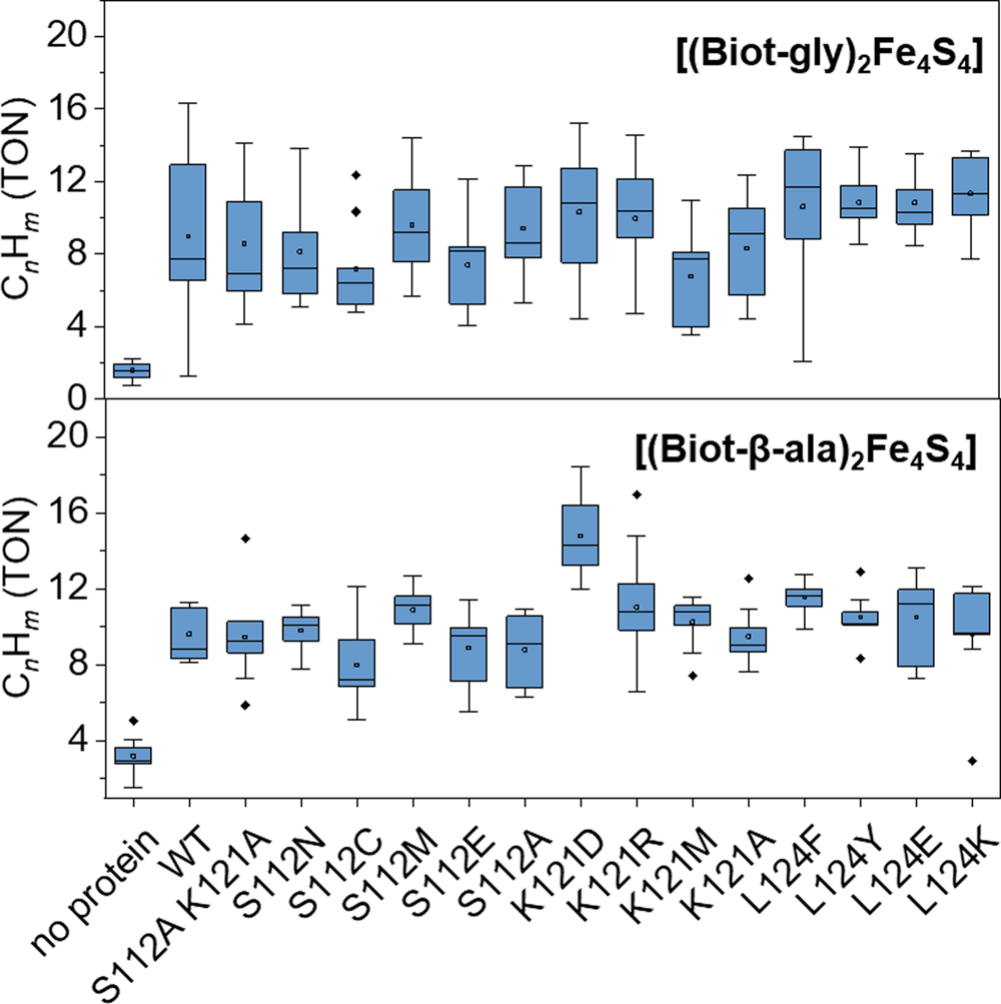
Chemo-genetic optimization of FTases based on the biotin–streptavidin technology. Top: in the presence of the cofactor bearing a glycine spacer: **[(Biot-gly)**_**2**_**Fe**_**4**_**S**_**4**_**]**·Sav isoform. Bottom: in the presence of the cofactor bearing an β-alanine spacer: **[(Biot-β-ala)**_**2**_**Fe**_**4**_**S**_**4**_**]**·Sav isoforms. For these experiments, the respective cofactor and an excess of Sav mutant were incubated 30 min prior to initiating the reaction by adding Eu(II)-DTPA. The experiments were performed three times in triplicates (*n* = 9) (box: interquartile range, whiskers: 1.5 times interquartile range, lines: median, squares: mean, diamonds: outliers).

Next, we turned to the chemo-genetic optimization of the FTase activity. For chemical optimization purposes, we synthesized and characterized a 3,5-bis(mercaptomethyl)benzene-bearing ligand with a β-alanine-spacer ((**Biot-β-ala**) hereafter) instead of the glycine spacer in (**Biot-gly**). (**Biot-β-ala**), **[(Biot-β-ala)**_**2**_**Fe**_**4**_**S**_**4**_**]**, and **[(Biot-β-ala)**_**2**_**Fe**_**4**_**S**_**4**_**]**·Sav isoforms were prepared as described in Sections 2.2 and 2.4. The corresponding characterization experiments are collected in the SI (see Figure S1 for stability assessments, S5 for CD titration, S8 for native HRMS, and S12, S17 for cyclic voltammograms). We evaluated the FTase activity of both **[(Biot-gly)**_**2**_**Fe**_**4**_**S**_**4**_**]** and **[(Biot-β-ala)**_**2**_**Fe**_**4**_**S**_**4**_**]** in the presence of various Sav mutants, Figure 6. Comparison of the catalytic activity of both FTases reveals that the introduction of mutations at either the S112 or K121 position affects FTase performance to a moderate extent, while mutations in position L124 have only marginal effects. The highest overall TON was observed for **[(Biot-β-ala)**_**2**_**Fe**_**4**_**S**_**4**_**]**·Sav K121D. This highlights the critical influence of second coordination sphere interactions between the biotinylated cofactor and close-lying residues. Further, this observation supports the hypothesis that the active catalyst is indeed embedded within Sav. Indeed, in the event of [Fe_4_S_4_] core decomposition or dissociation from its biotinylated ligand, identical catalytic activities would be expected for all FTases. However, more research is needed to understand the effects of the amino acid residues in the binding pocket on the catalytic activity.

## 3 Outlook

Capitalizing on the biotin–streptavidin technology, we have designed, engineered, and genetically improved an artificial Fischer–Tropschase to convert CO_2_ into alka/enes. Several noteworthy features were unraveled in the course of this study: (i) the coordination of the [Fe_4_S_4_] cluster to two 3,5-bis(mercaptomethyl)benzene ligands endows the corresponding cofactor with significantly improved aqueous stability. (ii) The design of a ligand bearing an amino acid spacer between the biotin anchor and the bis-thiolate moiety ensures the formation of discrete **[(Biot-gly)**_**2**_**Fe**_**4**_**S**_**4**_**]**·Sav FTases, rather than cross-linked Sav aggregates. This was confirmed by CD spectroscopy and native MS. (iii) Incorporating **[(Biot-gly)**_**2**_**Fe**_**4**_**S**_**4**_**]** within various Sav mutants significantly affected the redox properties of the corresponding ArM compared to the free cofactor **[(Biot-gly)**_**2**_**Fe**_**4**_**S**_**4**_**]**. (iv) The chemo-genetic optimization of the catalytic performance of the FTase strongly supports the hypothesis that the metalloprotein is critically involved in the catalytic transformation rather than merely acting as a source of FeS nanoparticles, which catalyze the CO_2_ reduction. Current efforts aim to optimize the FTase activity further by evaluating a large library of Sav isoforms, including chimeric streptavidin with a shielded active site.

## Supporting Information Available

Experimental procedures, GC traces and calibrations, computational details, and supplementary figures, cyclic voltammograms, and UV–vis, CD, and HRMS/NMR spectra (PDF)
Calculated structure of **[(Biot-gly)_2_Fe_4_S_4_]**·Sav WT (PDB)

## Supplementary Material

ja3c03546_si_001.pdf

ja3c03546_si_002.pdb
