# Supplementary material for: An Artificial [Fe4S4]-Containing Metalloenzyme for the Reduction of CO2 to Hydrocarbons
Source: J Am Chem Soc. 2023 Jun 30;145(27):14823–30. doi: 10.1021/jacs.3c03546 (PMC10347540; doi:10.1021/jacs.3c03546)
Supplement: Supplementary file 1 — ja3c03546_si_001.pdf [file ja3c03546_si_001.pdf]

Supporting Information for

# An Artificial [$Fe_4S_4$]-Containing Metalloenzyme for the Reduction of $CO_2$ to Hydrocarbons

Valerie Waser, Manjistha Mukherjee, Ryo Tachibana, Nico V. Igareta, Thomas R. Ward*

Department of Chemistry, University of Basel, BPR 1096, Mattenstrasse 22, 4058 Basel, Switzerland

*Email: thomas.ward@unibas.ch

# Contents

**I) Materials and Methods** .......... 1
*I.1) General Information* .......... 1
*I.2) Instrumentation* .......... 1
*I.3) Quantification of $C_nH_m$ Products* .......... 1
*I.4) Quantification of $H_2$* .......... 2
*I.5) GC-MS Analysis of $C_nH_m$ Products* .......... 2

**II) Synthesis** .......... 3
*II.1) Synthesis of **[(Biot-gly)$_2$Fe$_4$S$_4$]*** .......... 3
*II.2) Synthesis of **[(Biot-β-ala)$_2$Fe$_4$S$_4$]*** .......... 7

**III) Characterization of the Cofactors and ArMs** .......... 9
*III.1) Stability Assessments by UV-vis* .......... 9
*III.2) UV-vis Spectra of **[(Biot-gly)$_2$Fe$_4$S$_4$]**·Sav Isoforms and **[(Biot-β-ala)$_2$Fe$_4$S$_4$]**·Sav Isoforms* .... 11
*III.3) CD Titrations* .......... 12
*III.4) Native MS* .......... 14

**IV) Electrochemistry** .......... 15
*IV.1) Electrochemical Analysis of **[(Biot-gly)$_2$Fe$_4$S$_4$]** and **[(Biot-β-ala)$_2$Fe$_4$S$_4$]*** .......... 15
*IV.2) Electrochemical Analysis of **[(Biot-gly)$_2$Fe$_4$S$_4$]**·Sav Isoforms and **[(Biot-β-ala)$_2$Fe$_4$S$_4$]**·Sav AA* 18
*IV.3) Electrochemical Analysis of **[(Biot-gly)$_2$Fe$_4$S$_4$]**, Biot·Sav* .......... 21
*IV.4) Electrochemical Analysis of **[(Biot-gly)$_2$Fe$_4$S$_4$]**, BSA* .......... 22
*IV.5) CV Summary* .......... 23

**V) Modelling Studies** .......... 24

**VI) Catalysis** .......... 24
*VI.1) $CO_2$ Reduction Assay* .......... 24
*VI.2) $CO_2$ Reduction in Phosphate Buffer* .......... 25
*VI.3) $CO_2$ Reduction with $^{13}CO_3HNa$ or $^{12}CO_3HNa$* .......... 26
*VI.4) Kinetics of $CO_2$ Reduction* .......... 27
*VI.5) Addition of Multiple Eu(II)-DTPA Aliquots* .......... 28

**VII) GC Traces and Calibration** .......... 30
*VII.1) GC Calibrations* .......... 30
*VII.2) GC Traces* .......... 31

**VIII) References** .......... 33

**IX) HRMS and NMR Spectra** .......... 35

# I) Materials and Methods

### I.1) General Information

Chemicals were purchased from Sigma Aldrich, Acros Organics, Alfa Aesar or Fluorochem and used without further purification. $CO_2$ with the purity grade 5.3 was obtained from Pangas. Dry solvents were directly purchased from Acros Organics. Water used for molecular biology, catalytic reactions, and electrochemistry was purified by a Milli-Q Advantage system. All [$Fe_4S_4$] clusters were handled under the strict exclusion of air using Schlenk techniques or a MBraun Labstar Glovebox operating under $N_2$ with <0.1 ppm $O_2$. Degassed solvents were prepared via three to five freeze-pump-thaw cycles. Water and buffers were degassed by purging with $N_2$ overnight. Streptavidin (Sav) mutants were produced, purified and characterized as previously described.[1] All listed concentrations refer to homotetrameric Sav.

### I.2) Instrumentation

$^1$H NMR (500 or 600 MHz) and $^{13}$C NMR (126 or 151 MHz) spectra were recorded at RT on a Bruker 500 MHz or 600 MHz spectrometer. HRMS was performed on a Bruker maXis II QTOF ESI mass spectrometer coupled to a Shimadzu liquid chromatography instrument. Air sensitive compounds were injected directly in into the MS device (circumventing preceding liquid chromatography). CD spectra were recorded on a Chirascan spectrometer (AppliedPhotophyscis) at 25 °C using the following parameters: step resolution 3 nm, bandwidth: 1 nm, time per point: 5 s. UV-vis spectra were recorded on a Varian Carry 50 Scan.

### I.3) Quantification of $C_nH_m$ Products

Hydrocarbon products were detected by gas chromatography with a flame ionization detector (GC-FID). $C_1$–$C_4$ products in the kinetic experiments were measured on an

Agilent 6890 chromatograph equipped with an HP-PLOT Q column (30 m, 0.32 mm ID, Agilent). As a carrier gas helium was used with a flow rate of 8.6 mL/min. The samples (250 µL) were injected manually with a gastight syringe (Hamilton). The separation of the products was achieved with a temperate gradient starting from 60 °C (held for 2 min) and then heating to 240 °C at a rate of 30 °C/min. In all other experiments, $C_1$–$C_4$ products were detected on a SRI 8610C chromatograph (SRI instruments) equipped with a Hayesep D column (3 m, 2 mm ID, mesh 80/100, SRI). As a carrier gas, $N_2$ was used with a flow rate of 24 mL/min. The samples (1 mL) were injected with an autosampler (HT2000H, HTA instruments). The separation of the products was achieved with a temperate gradient starting from 70 °C (held for 2 min) and then heating to 220 °C at a rate of 10 °C/min. Both instruments were calibrated using commercial mixture of $CH_4$, $C_2H_6$, $C_2H_8$, $C_3H_8$, $C_3H_{10}$, $C_4H_{10}$ and $C_4H_{12}$ ($C_1$–$C_2$: 1000 ppm and $C_3$–$C_4$: 100 ppm) (Pangas).

### I.4) Quantification of $H_2$

$H_2$ was detected with the SRI device and a TCD detector (GC-TCD). The same method as in section I.3 was used. The instrument was calibrated using commercial $H_2$ (Pangas). For the kinetics experiments, the samples (250 µL) were injected manually with a Hamilton syringe.

### I.5) GC-MS Analysis of $C_nH_m$ Products

GC-MS analyses were performed on a Shimadzu GCMS-QP2010S equipped with a HP-PLOT Q column (30 m, 0.32 mm ID, Agilent). The samples (250 µL) were injected manually with a Hamilton syringe. As a carrier gas, helium was used with a flow rate of 12.8 mL/min. The separation of the products was achieved with a temperature gradient starting from 50 °C and heating to 100 °C at a rate of 10 °C/min, then keeping it at 100 °C for 1 min.

## II) Synthesis

*Tert*-butyl (3,5-bis(chloromethyl)phenyl)carbamate,[2] biotin-$OC_6F_5$,[3] $(NMe_4)_2[(t\mathrm{BuS})_4Fe_4S_4]$,[4] and $((NMe_4)_2[(SCH_2CH_2OH)_4Fe_4S_4])$ (cluster **6**)[5] were synthesized according to literature procedures.

### II.1) Synthesis of $[(\mathrm{Biot\text{-}gly})_2Fe_4S_4]$

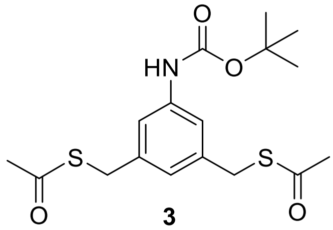

**3**

**Compound 3** To a solution of *tert*-butyl (3,5-bis(chloromethyl)phenyl)carbamate (850 mg, 2.9 mmol) and thioacetic acid (0.8 mL, 11.7 mmol) in THF (40 mL), potassium carbonate (1620 mg, 11.7 mmol) was added as a solid. The mixture was heated to reflux overnight. Water was added, and the product was extracted with EtOAc. The organic layers were combined, dried over sodium sulphate, filtered, and the solvent was removed under vacuum. The crude product was purified by column chromatography (9:1 Cyhex/EtOAc) to isolate the bis(thioester) **3** (950 mg, 88%) as a yellowish solid. **$^1$H NMR** (500 MHz, $CDCl_3$) δ 7.16 (s, 2H), 6.84 (s, 1H), 6.44 (s, 1H), 4.01 (s, 4H), 2.31 (s, 6H), 1.47 (s, 9H, C6H). **$^{13}$C NMR** (126 MHz, $CDCl_3$) δ 195.35, 152.87, 139.25, 139.22, 124.11, 118.02, 33.57, 30.65, 28.65. **HRMS** (ESI positive mode, m/z) calcd. for $C_{17}H_{23}NO_4S_2Na$ ($[M+Na]^+$): 392.0961, found: 392.0964.

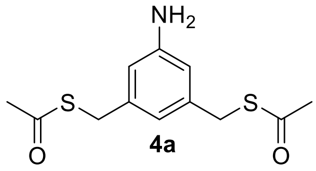

**4a**

**Intermediate 4a** To a solution of bis(thioester) **3** (1083 mg, 2.9 mmol) in DCM (20 mL), TFA (6.8 mL, 87.9 mmol) was added via a syringe. The mixture was stirred overnight at RT. The solvent was removed under vacuum to afford the crude amine **4a** as a yellowish gum. **$^1$H NMR** (500 MHz, $CDCl_3$) δ7.15 (s, 1H) 7.14 (s, 2H), 4.05 (s, 4H), 2.35 (s, 6H). **$^{13}$C NMR** (126 MHz, $CDCl_3$) δ 196.87, 141.30, 130.68, 130.33, 122.57, 32.94, 30.51.

**HRMS** (ESI positive mode, m/z) calcd. for $C_{12}H_{16}NO_2S_2$ ($[M+H]^+$): 270.0617, found: 270.0620.

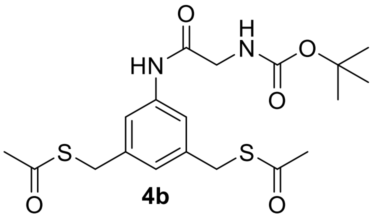

**Intermediate 4b** To a solution of amine **4a** (1112 mg, 2.9 mmol) in DMF (20 mL), HATU (1103 mg, 2.9 mmol) and n-Boc-Gly-OH (660 mg, 3.8 mmol) were added as a solid, and TEA (1.68 mL, 10.1 mmol) was added via a syringe. The mixture was stirred at RT for 1 h, poured into water, and extracted with EtOAc. The crude product was purified by column chromatography (7:3 Cyhex/EtOAc) to isolate the amide **4b** (1114 mg, 90%) as a yellow gum. **$^1$H NMR** $^1$H NMR (500 MHz, $CDCl_3$) δ 8.22 (s, 1H), 7.32 (s, 2H), 6.92 (s, 1H), 4.01 (s, 4H), 2.31 (s, 6H), 1.44 (s, 8H). **$^{13}$C NMR** (126 MHz, $CDCl_3$) δ 195.06, 167.93, 167.30 139.05, 137.96, 127.61, 125.26, 119.20, 33.15, 30.33, 28.32, 26.92. **HRMS** (ESI positive mode, m/z) calcd. for $C_{19}H_{26}N_2O_5S_2Na$ ($[M+Na]^+$): 449.1175, found: 449.1183.

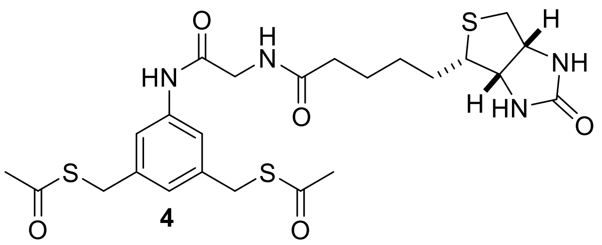

**Compound 4** To a solution of amide **4b** (1028 mg, 2.4 mmol) in DCM (15 mL), TFA (2.8 mL, 36.2 mmol) was added via a syringe. The mixture was stirred at RT overnight and the solvent was removed under vacuum. The residue was dissolved in DMF (15 mL), and the mixture was cooled to 0 °C in an ice bath. Biotin-$OC_6F_5$ (855 mg, 2.2 mmol) was added as a solid, and TEA (1.4 mL, 9.6 mmol) was added via a syringe. The mixture was stirred in the thawing ice bath overnight. The solvent was removed under vacuum and the residue was washed with diethyl ether and acetone to afford the white solid biotin-derivative **4** (1162 mg, 87%). **$^1$H NMR** (500 MHz, DMSO-$d_6$) δ 9.98 (s, 1H), 8.11 (t, $J$ = 5.9 Hz, 1H), 7.41 (s, 2H), 6.90 (s, 1H), 6.41 (s, 1H), 6.35 (s, 1H), 4.30 (dd, $J$ = 7.7, 5.0 Hz, 1H), 4.16 – 4.10 (m, 1H), 4.04 (s, 4H), 3.82 (d, $J$ = 5.8

Hz, 2H), 3.09 (m, 1H), 2.82 (dd, $J$ = 12.4, 5.1 Hz, 1H), 2.58 (d, $J$ = 12.4 Hz, 1H), 2.35 (s, 6H), 2.19 – 2.11 (m, 2H), 1.70 – 1.24 (m, 6H). **$^{13}$C NMR** (126 MHz, DMSO-$d_6$) δ 195.49, 173.53, 168.93, 163.63, 140.18, 139.57, 124.71, 118.98, 61.94, 60.12, 56.34, 43.53, 35.84, 33.25, 31.18, 29.11, 28.95, 26.10. **HRMS** (ESI positive mode, m/z) calcd. for $C_{24}H_{32}N_4O_5S_3$ ([M+Na]$^+$): 575.1433, found: 575.1435.

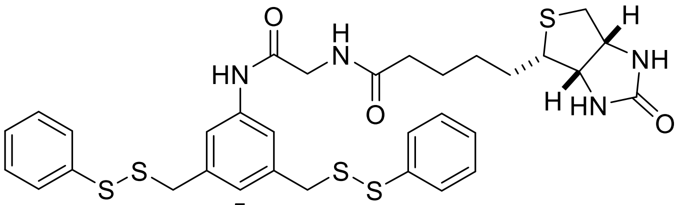

**5**

**Compound 5** A suspension of biotin-derivative **4** (1142 mg, 2.1 mmol) in MeOH (5 mL) was cooled to 0 °C in an ice bath. 2 M ammonia in MeOH (15 ml, 30 mmol) was added dropwise via a syringe. The mixture was stirred at 0 °C for 5 h and the solvent was removed under vacuum. The residue was suspended in DMF (10 mL), and phenyl disulfide (4650 mg, 21.3 mmol) was added as a solid. The mixture was stirred at RT for 2 h and the solvent was removed under vacuum. The residue was washed copiously with diethyl ether. The crude product was purified by column chromatography (0 to 12% MeOH in DCM) to isolate the grey solid bis(disulfide) **5** (260 mg, 18%). **$^1$H NMR** (500 MHz, DMSO-$d_6$) δ 9.97 (s, 1H), 8.13 (t, $J$ = 5.9 Hz, 1H), 7.51 – 7.45 (m, 6H), 7.35 (dd, $J$ = 8.5, 6.9 Hz, 4H), 7.29 – 7.25 (m, 2H), 6.93 (t, $J$ = 1.7 Hz, 1H), 6.42 (t, $J$ = 1.8 Hz, 1H), 6.36 (s, 1H), 4.30 (dd, $J$ = 7.8, 5.1 Hz, 1H), 4.14 (ddd, $J$ = 7.8, 4.4, 1.9 Hz, 1H), 3.96 (s, 4H), 3.85 (d, $J$ = 5.8 Hz, 2H), 3.11 (ddd, $J$ = 8.6, 6.2, 4.3 Hz, 1H), 2.82 (dd, $J$ = 12.4, 5.1 Hz, 1H), 2.58 (d, $J$ = 12.4 Hz, 1H), 2.17 (td, $J$ = 7.2, 2.1 Hz, 2H), 1.69 – 1.27 (m, 6H). **$^{13}$C NMR** (126 MHz, DMSO-$d_6$) δ 173.57, 168.93, 163.63, 140.09, 138.37, 137.14, 130.15, 128.44, 128.08, 126.16, 119.86, 61.94, 60.13, 56.35, 43.58, 43.11, 35.87, 29.11, 28.96, 26.12. **HRMS** (ESI positive mode, m/z) calcd. for $C_{32}H_{36}N_4O_3S_5$ ([M+H]$^+$): 685.1464, found: 685.1458.

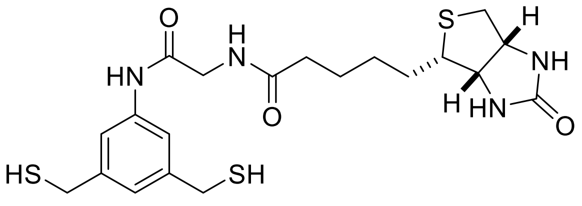


**(Biot-gly)**

**(Biot-gly)** To a solution of bis(disulfide) **5** (250 mg, 0.4 mmol) in DMF (5 mL), DTT (563 mg, 3.7 mmol) was added as a solid. The mixture was stirred overnight at RT and the solvent was removed under vacuum. The residue was washed copiously with diethyl ether and acetone to afford the white solid dithiol **(Biot-gly)** (140 mg, 82%). **$^{1}$H NMR** (500 MHz, DMSO-$d_6$) δ 9.95 (s, 1H), 8.12 (t, *J* = 5.8 Hz, 1H), 7.44 (d, *J* = 1.6 Hz, 2H), 6.99 (s, 1H), 6.41 (s, 1H), 6.36 (s, 1H), 4.35 – 4.28 (m, 1H), 4.19 – 4.12 (m, 1H), 3.84 (d, *J* = 5.9 Hz, 2H), 3.69 (d, *J* = 7.6 Hz, 4H), 3.11 (t, *J* = 9.5 Hz, 1H), 2.82 (m, *J* = 7.4, 3.7 Hz, 3H), 2.58 (d, *J* = 12.4 Hz, 1H), 2.17 (dd, *J* = 8.5, 6.2 Hz, 2H), 1.68 – 1.26 (m, 6H). **$^{13}$C NMR** (151 MHz, DMSO-$d_6$) δ 173.09, 168.41, 142.52, 139.51, 123.35, 117.79, 61.46, 59.65, 55.89, 43.09, 28.65, 28.49, 28.22, 25.65. **HRMS** (ESI positive mode, m/z) calcd. for $C_{20}H_{28}N_4O_3S_3$ ($[M+H]^+$): 469.1396, found: 469.1399.

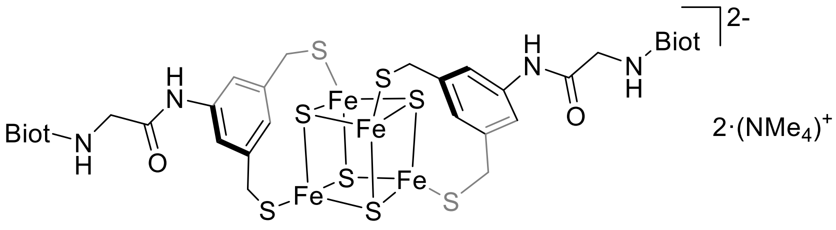


**[(Biot-gly)$_2$Fe$_4$S$_4$]**

**[(Biot-gly)$_2$Fe$_4$S$_4$]** To a solution of $(NMe_4)_2[(t\text{BuS})_4Fe_4S_4]$ (8.4 mg, 0.01 mmol) in DMF (2 mL), a solution of dithiol **(Biot-gly)** (9.4 mg, 0.02 mmol) in DMF (2 mL) was added via a syringe. The mixture was stirred under reduced pressure for 1 h at RT. The solvent was removed under vacuum to afford the crude black solid cluster **[(Biot-gly)$_4$Fe$_4$S$_4$]**. **$^{1}$H NMR** (600 MHz, DMSO-$d_6$) δ 16.48 (s, 8H), 9.86 (s, 2H), 8.13 (s, 2H), 7.56 (s, 6H), 6.44 (s, 2H), 6.34 (s, 2H), 4.30 (m, 2H), 4.14 (m, 2H), 3.92 (s, 4H), 3.11 (m, 2H), 3.09 (s, 24H), 2.86 – 2.79 (m, 2H), 2.57 (d, *J* = 12.4 Hz, 2H), 2.16 (t, *J* = 7.0 Hz, 4H), 1.67 – 1.20 (m,

12H). **HRMS** (ESI negative mode, m/z) calcd. for $C_{40}H_{52}Fe_4N_8O_6S_{10}$ ($[M–2(NMe_4^+)]^{2-}$): 641.9313, found: 641.9326.

### II.2) Synthesis of [(Biot-β-ala)$_2$Fe$_4$S$_4$]

**[(Biot-β-ala)$_2$Fe$_4$S$_4$]** was prepared following the same synthetic route as in section II.1 except that for the preparation of intermediate **4b'** N-Boc-β-Ala-OH was used instead of N-Boc-Gly-OH.

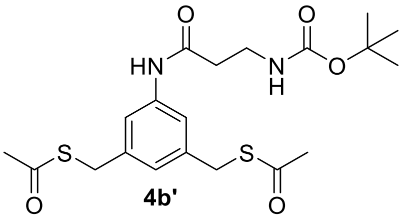


**4b'**

**Intermediate 4b'** The product was isolated as a yellowish solid (128 mg, 86%). **$^1$H NMR** (500 MHz, $CDCl_3$) δ 7.64 (s, 1H), 7.38 (d, *J* = 1.5 Hz, 2H), 6.95 (t, *J* = 1.6 Hz, 1H), 5.15 (s, 1H), 4.05 (s, 4H), 3.48 (q, *J* = 6.0 Hz, 2H), 2.57 (t, *J* = 5.9 Hz, 2H), 2.35 (s, 6H), 1.44 (s, 9H). **$^{13}$C NMR** (151 MHz, $CDCl_3$) δ 195.08, 169.72, 156.33, 139.02, 138.31, 125.06, 118.94, 79.71, 37.62, 36.37, 33.18, 30.37, 28.42. **HRMS** (ESI positive mode, m/z) calcd. for $C_{20}H_{28}N_2O_5S_2Na$ ($[M+Na]^+$): 463.1332, found: 463.1336.

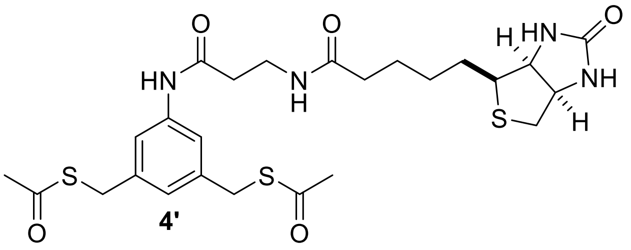


**4'**

**Compound 4'** The product was isolated as a white solid (120 mg, 80%). **$^1$H NMR** (500 MHz, DMSO-$d_6$) δ 9.96 (s, 1H), 7.90 (t, *J* = 5.7 Hz, 1H), 7.43 (d, *J* = 1.7 Hz, 2H), 6.88 (d, *J* = 1.7 Hz, 1H), 6.39 (t, *J* = 1.9 Hz, 1H), 6.34 (s, 1H), 4.25 (ddt, *J* = 7.5, 5.1, 1.1 Hz, 1H), 4.11 – 4.04 (m, 1H), 4.04 (s, 4H), 3.29 (m, 2H), 3.03 (m 1H), 2.76 (dd, *J* = 12.4, 5.1 Hz, 1H), 2.55 (d, *J* = 12.5 Hz, 1H), 2.45 (t, *J* = 6.8 Hz, 2H), 2.35 (s, 6H), 2.08 – 2.00 (m, 2H), 1.64 – 1.20 (m, 6H). **$^{13}$C NMR** (151 MHz, DMSO-$d_6$) δ 194.62, 172.18, 169.64, 162.72, 139.62, 138.55, 123.65, 117.97, 60.99, 59.19, 55.45, 45.71,

36.37, 35.82, 34.95, 32.37, 30.30, 28.14, 28.03, 25.33. **HRMS** (ESI positive mode, m/z) calcd. for $C_{25}H_{34}N_4O_5S_3$ ([M+Na]$^+$): 589.1584, found: 589.1587.

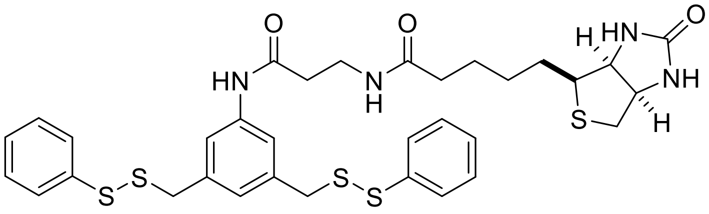


**5'**

**Compound 5'** The product was isolated as an off-white solid (22 mg, 15%).**$^1$H NMR** (600 MHz, DMSO-$d_6$) δ 9.97 (s, 1H), 7.92 (t, *J* = 5.8 Hz, 1H), 7.51 – 7.46 (m, 6H), 7.36 (t, *J* = 7.6 Hz, 4H), 7.29 – 7.24 (m, 2H), 6.92 (s, 1H), 6.41 (s, 1H), 6.36 (s, 1H), 4.26 (dd, *J* = 7.7, 5.1 Hz, 1H), 4.07 (t, *J* = 6.2 Hz, 1H), 3.96 (s, 4H), 3.30 (t, *J* = 6.4 Hz, 2H), 3.07 – 3.01 (m, 1H), 2.77 (dd, *J* = 12.5, 5.0 Hz, 1H), 2.55 (d, *J* = 12.5 Hz, 1H), 2.47 (t, *J* = 6.9 Hz, 2H), 2.05 (t, *J* = 7.4 Hz, 2H), 1.63 – 1.24 (m, 6H). **$^{13}$C NMR** (151 MHz, DMSO-$d_6$) δ 172.62, 170.05, 163.16, 139.97, 137.79, 136.68, 129.71, 127.95, 127.63, 125.55, 119.34, 61.44, 59.63, 55.89, 42.66, 36.88, 35.55, 35.42, 28.61, 28.48, 25.76. **HRMS** (ESI positive mode, m/z) calcd. for $C_{33}H_{38}N_4O_3S_5$ ([M+H]$^+$): 721.1440, found: 721.1444.

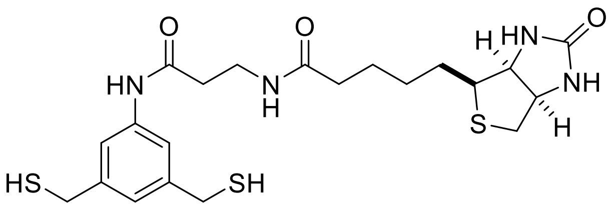


**(Biot-β-ala)**

**(Biot-β-ala)** The product was isolated as a white solid (11 mg, 79%). **$^1$H NMR** (600 MHz, DMSO-$d_6$) δ 9.97 (s, 1H), 7.93 (t, *J* = 5.8 Hz, 1H), 7.47 (s, 2H), 6.98 (s, 1H), 6.42 (s, 1H), 6.37 (s, 1H), 4.27 (t, *J* = 6.4 Hz, 1H), 4.08 (t, *J* = 6.0 Hz, 1H), 3.68 (s, 4H), 3.31 (q, *J* = 6.5 Hz, 2H), 3.09 – 3.00 (m, 1H), 2.81 (s, 2H), 2.77 (dd, *J* = 12.5, 5.1 Hz, 1H), 2.56 (d, *J* = 12.4 Hz, 1H), 2.47 (t, *J* = 6.9 Hz, 2H), 2.05 (t, *J* = 7.4 Hz, 2H), 1.63 – 1.21 (m, 6H). **$^{13}$C NMR** (151 MHz, DMSO-$d_6$) 172.02, 169.39, 162.57, 141.78, 139.24, 122.57, 117.12, 60.82, 59.02, 55.27, 39.89, 36.25, 34.94, 34.85, 27.98, 27.87, 27.65, 25.17. **HRMS** (ESI positive mode, m/z) calcd. for $C_{21}H_{30}N_4O_3S_3$ ([M+K]$^+$): 521.1112, found: 521.1110.

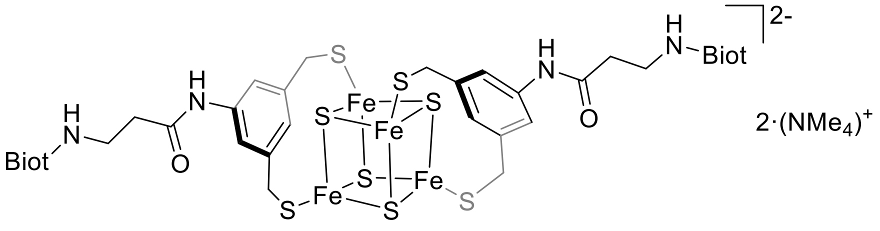


**[(Biot-β-ala)$_2$Fe$_4$S$_4$]**

**[(Biot-β-ala)$_2$Fe$_4$S$_4$]** The crude product was obtained as a black solid. **$^1$H NMR** (600 MHz, DMSO-*d*$_6$) δ 16.48 (s, 8H), 9.86 (s, 2H), 7.97 (s, 2H), 7.59 (s, 6H), 6.39 (s, 2H), 6.33 (s, 2H), 4.28 (s, 2H), 4.10 (s, 2H), 3.32 (m, 4H), 3.09 (s, 24H), 3.06 (m, 2H), 2.80 (m, 2H), 2.57 (m, 2H), 2.04 (t, $J$ = 6.9 Hz, 4H), 1.62 – 1.22 (m, 12H). **HRMS** (ESI negative mode, m/z) calcd. for $C_{42}H_{56}Fe_4N_8O_6S_{10}$ ([M–2(NMe$_4^+$)]$^{2-}$): 655.9469, found: 655.9482.

# III) Characterization of the Cofactors and ArMs

### III.1) Stability Assessments by UV-vis

A solution of **[(Biot-gly)$_2$Fe$_4$S$_4$]** in DMSO (5 mM stock solution, final concentration 50 µM) respectively $(NMe_4)_2[(SCH_2CH_2OH)_4Fe_4S_4]$ in DMSO (5 mM stock solution, final concentration 50 µM) was placed into a gas-tight quartz cuvette and diluted with various mixtures of DMSO and borate buffer (0.2 M, pH 8.2) to a final volume of 200 µL. The spectra were recorded after 15 min, 1 h and 18 h. **[(Biot-gly)$_2$Fe$_4$S$_4$]**·Sav AA: A solution of Sav AA (250 µM stock solution, final concentration 25 µM) in borate buffer (0.2 M, pH 8.2) was added to the cuvettes before dilution. A similar method was used for the stability assessment of **[(Biot-β-ala)$_2$Fe$_4$S$_4$]** and **[(Biot-β-ala)$_2$Fe$_4$S$_4$]**·Sav AA. For the spectra presented in Figure S2, **[(Biot-gly)$_2$Fe$_4$S$_4$]** was dissolved in DMF instead of DMSO.

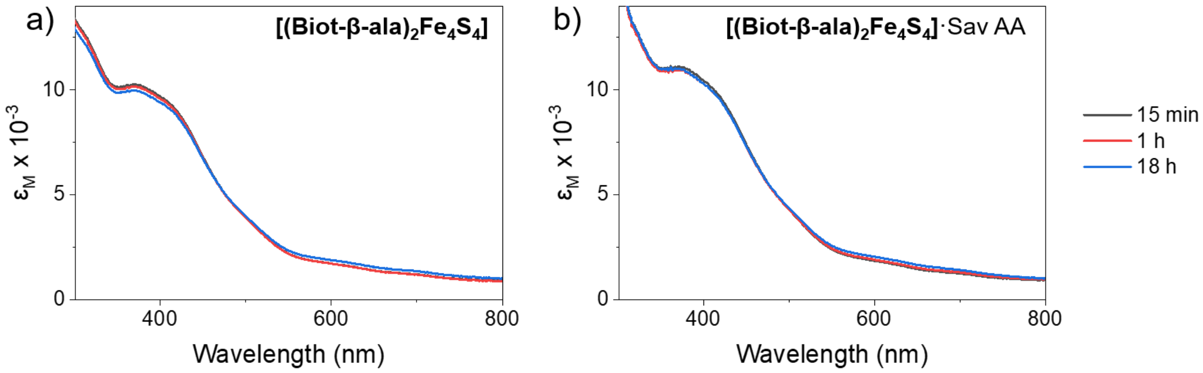


**Figure S1.** UV-vis spectra of **[(Biot-β-ala)$_2$Fe$_4$S$_4$]** and **[(Biot-β-ala)$_2$Fe$_4$S$_4$]**·Sav AA in a 1% DMSO−99% borate buffer (pH 8.2, 0.2 M) mixture. a) UV-vis spectra of **[(Biot-β-ala)$_2$Fe$_4$S$_4$]** at different time points. b) UV-vis spectra of **[(Biot-β-ala)$_2$Fe$_4$S$_4$]**·Sav AA at different time points.

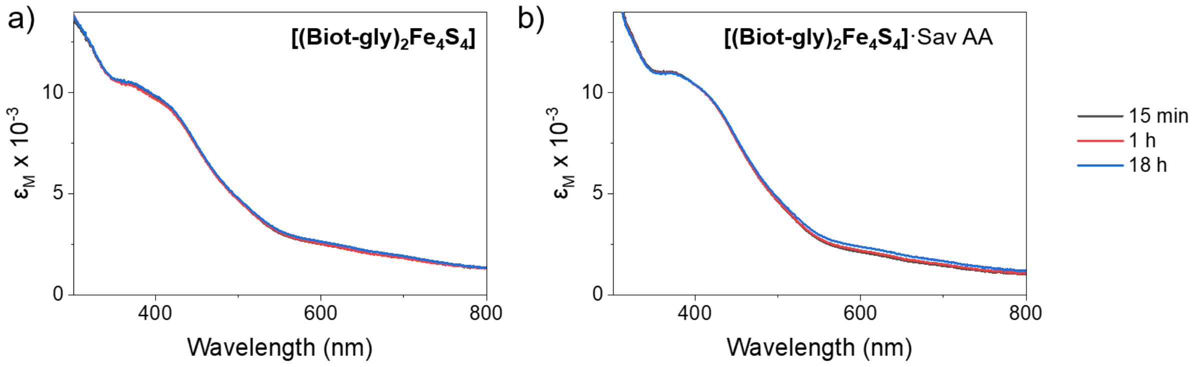


**Figure S2.** UV-vis spectra of **[(Biot-gly)$_2$Fe$_4$S$_4$]** and **[(Biot-gly)$_2$Fe$_4$S$_4$]**·Sav AA in a 1% DMF−99% borate buffer (pH 8.2, 0.2 M) mixture. a) UV-vis spectra of **[(Biot-gly)$_2$Fe$_4$S$_4$]** at different time points. b) UV-vis spectra of **[(Biot-gly)$_2$Fe$_4$S$_4$]**·Sav AA at different time points.

## III.2) UV-vis Spectra of [(Biot-gly)$_2$Fe$_4$S$_4$]·Sav Isoforms and [(Biot-β-ala)$_2$Fe$_4$S$_4$]·Sav Isoforms

A solution of **[(Biot-gly)$_2$Fe$_4$S$_4$]** in DMSO (5 mM stock solution, final concentration 50 µM) was added to a solution of different Sav isoforms in a borate buffer (0.2 M, pH 8.2) (250 µM stock solution, final concentration 25 µM). The spectra were recorded in gas-tight quartz cuvettes (200 µL final volume).

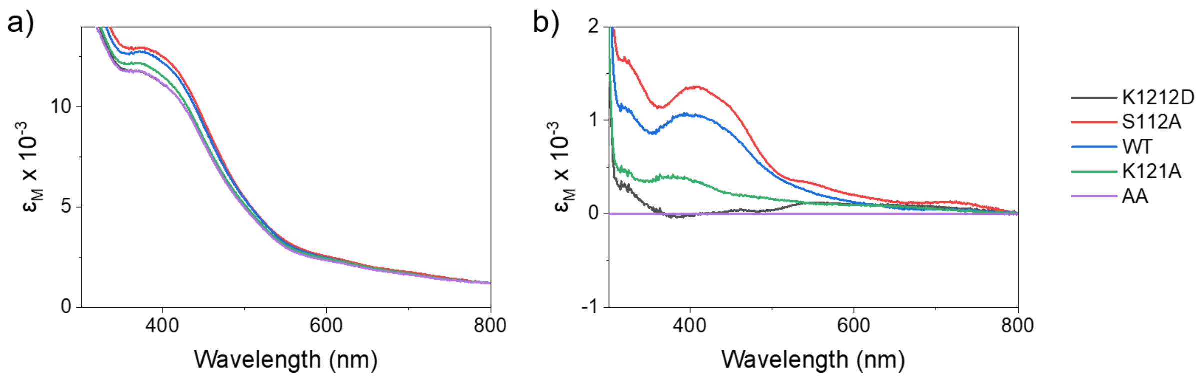


**Figure S3.** UV-vis spectra of **[(Biot-gly)$_2$Fe$_4$S$_4$]**·Sav isoforms in a 1% DMSO−99% borate buffer (pH 8.2, 0.2 M) mixture. a) UV-vis spectra of **[(Biot-gly)$_2$Fe$_4$S$_4$]**·Sav isoforms. b) Difference in absorbance of **[(Biot-gly)$_2$Fe$_4$S$_4$]**·Sav isoforms and **[(Biot-gly)$_2$Fe$_4$S$_4$]**·Sav AA.

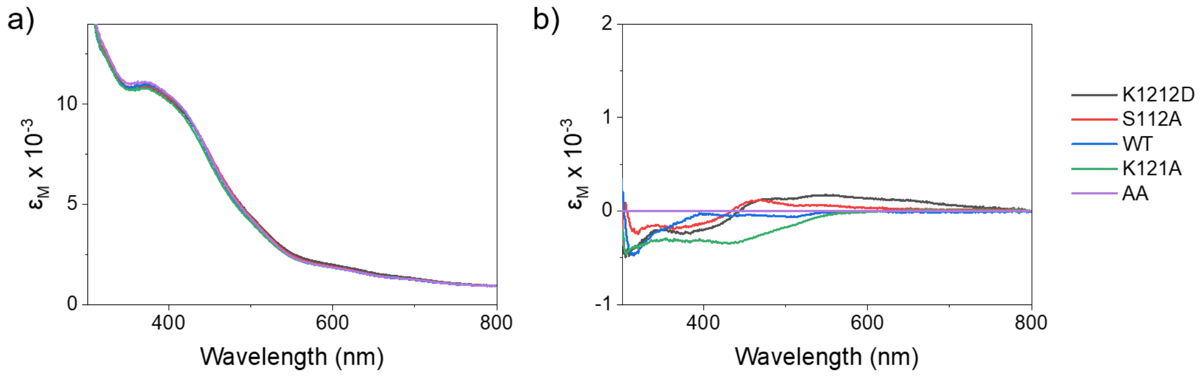


**Figure S4.** UV-vis spectra of **[(Biot-β-ala)$_2$Fe$_4$S$_4$]**·Sav isoforms in a 1% DMSO−99% borate buffer (pH 8.2, 0.2 M) mixture. a) UV-vis spectra of **[(Biot-β-ala)$_2$Fe$_4$S$_4$]**·Sav isoforms. b) Difference in absorbance of **[(Biot-β-ala)$_2$Fe$_4$S$_4$]**·Sav isoforms and **[(Biot-β-ala)$_2$Fe$_4$S$_4$]**·Sav AA.

**III.3) CD Titrations**

CD spectra were measured in a gas-tight quartz cuvette equipped with a screw cap with a septum. A solution of Sav S112A K121A (Sav AA hereafter, 250 µM stock solution, final concentration 12.5 µM) in borate buffer (200 mM, pH 8.2) was diluted with a mixture of the same borate buffer and DMF to a total volume of 2 mL (final concentration of DMF 9.1% v/v). Aliquots of **[(Biot-gly)$_2$Fe$_4$S$_4$]** (7.6 mM stock solution in DMF) were added in 0.3 equivalence steps (1 µL per step up to 4 equivalents) with a gastight syringe. The concentration of DMF was held constant by adding 10 µL borate buffer for each step. A similar titration was used for **[(Biot-β-ala)$_2$Fe$_4$S$_4$]**·Sav AA, using the respective concentrations: Sav AA (6.25 µM) and **[(Biot-β-ala)$_2$Fe$_4$S$_4$]** (5 mM).

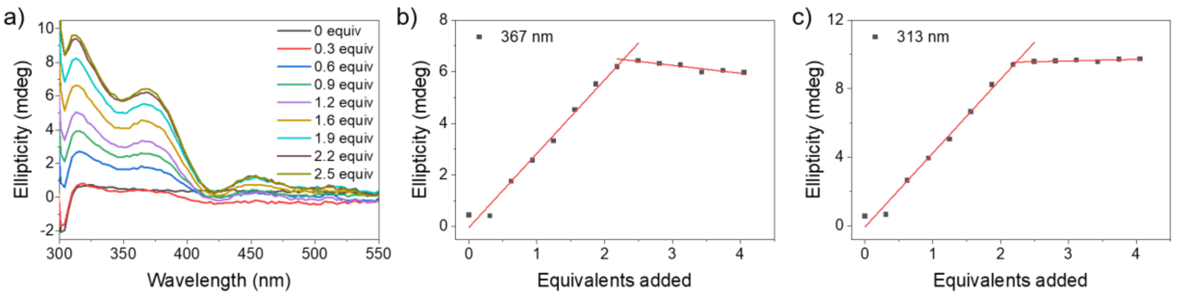


**Figure S5.** CD Titration of **[(Biot-gly)$_2$Fe$_4$S$_4$]**·Sav AA a) CD titration of Sav AA with **[(Biot-gly)$_2$Fe$_4$S$_4$]** revealing the appearance of three CD bands ($\lambda_{max}$ = 313 nm, $\lambda_{max}$ = 367 nm and $\lambda_{max}$ = 454 nm. b) Monitoring of the resulting molar ellipticity at 367 nm: the equivalence points lies at 2.3 equivalents. c) Monitoring of the resulting molar ellipticity at 316 nm: the equivalence points lies at 2.2 equivalents.

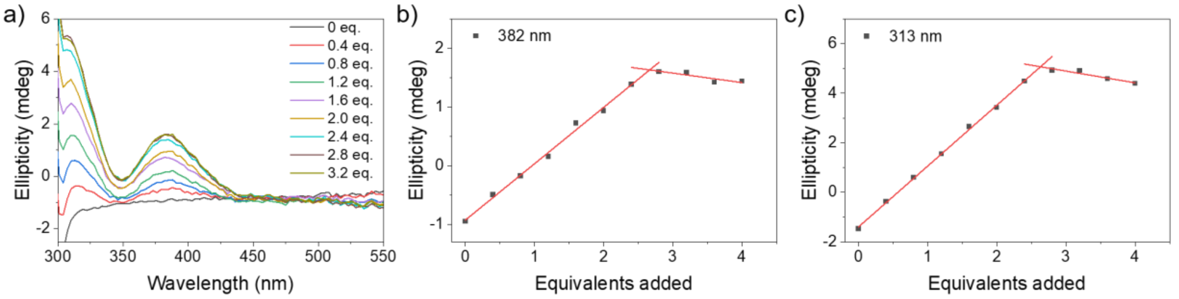


**Figure S6.** CD Titration of **[(Biot-β-ala)$_2$Fe$_4$S$_4$]**·Sav AA a) CD titration of Sav AA with **[(Biot-β-ala)$_2$Fe$_4$S$_4$]** revealing the appearance of two CD bands ($\lambda_{max}$ = 313 nm, $\lambda_{max}$ = 382 nm). b) Monitoring of the resulting molar ellipticity at 316 nm: the equivalence points lies at 2.6 equivalents. c) Monitoring of the resulting molar ellipticity at 382 nm: the equivalence points lies at 2.6 equivalents.

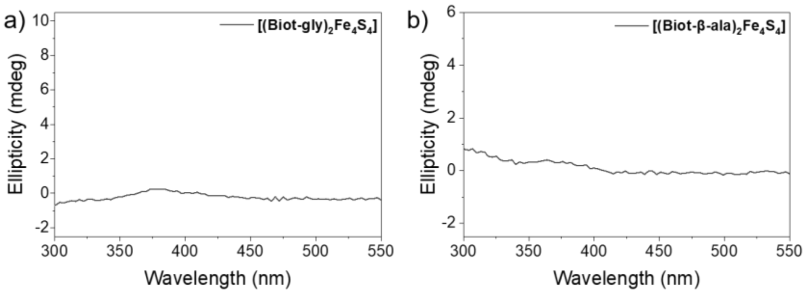


**Figure S7.** CD spectra of the unbound [$Fe_4S_4$] cofactors. a) CD Spectrum of **[(Biot-gly)$_2$Fe$_4$S$_4$]** (10 µM) in a mixture of borate buffer (200 mM, pH 8.2) and DMF (0.8% v/v). b) CD spectrum of **[(Biot-β-ala)$_2$Fe$_4$S$_4$]** (6.25 µM) in a mixture of borate buffer (200 mM, pH 8.2) and DMF (0.5% v/v).

### III.4) Native MS

Sav WT (250 µM stock solution in $H_2O$, final concentration 40 µM) and **[(Biot-gly)$_2$Fe$_4$S$_4$]** (10 mM stock solution in DMF, final concentration 100 µM) respectively **[(Biot-β-ala)$_2$Fe$_4$S$_4$]** (10 mM stock solution in DMF, final concentration 100 µM) were incubated for 2 h in an $NH_4OAc$ solution (50 mM). The solution was centrifuged and the supernatant was injected into a Bruker maXis ESI-QTof. The mass spectrometer was calibrated with ESI-Tof TuneMix (Agilent). The charge envelope was deconvoluted using the Compass DataAnalysis software (Bruker Daltonics).

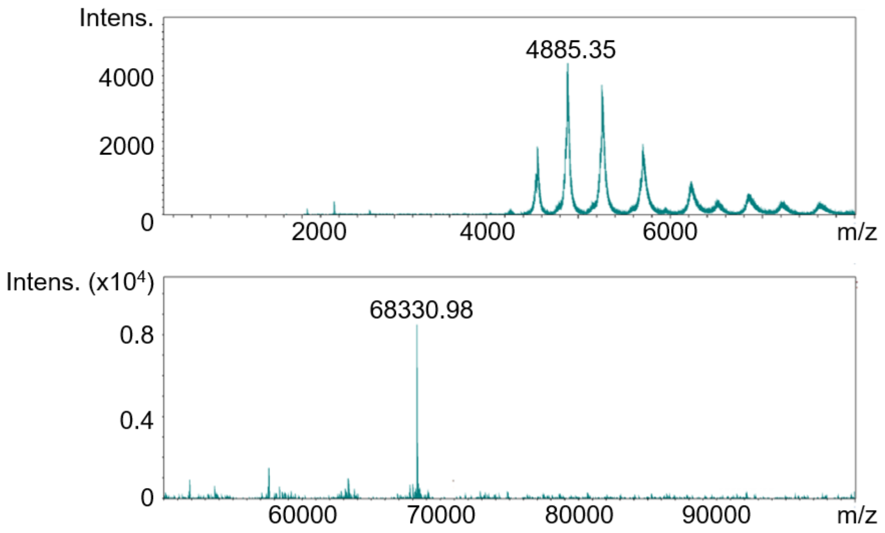


**Figure S8.** Native mass spectrum of **[(Biot-β-ala)$_2$Fe$_4$S$_4$]$_2$**·Sav WT: charge-state envelope (top) and deconvoluted (i.e., summed zero-charge mass distribution, bottom) mass spectrum resulting from incubating 2 equiv **[(Biot-β-ala)$_2$Fe$_4$S$_4$]$_2$** with 1 equiv homotetrameric Sav (calculated for **[(Biot-β-ala)$_2$Fe$_4$S$_4$]$_2$**·Sav: 68329.6 m/z, found: 68330.98 m/z).

# IV) Electrochemistry

## IV.1) Electrochemical Analysis of [(Biot-gly)$_2$Fe$_4$S$_4$] and [(Biot-β-ala)$_2$Fe$_4$S$_4$]

All cyclic voltammetry (CV) experiments were conducted under anaerobic conditions in a conventional three-electrode single-compartment cell. A CH-600D potentiostat was used for all the experiments. All potentials were referenced to an Ag/AgCl (saturated KCl) reference electrode. For the CV experiments of **[(Biot-gly)$_2$Fe$_4$S$_4$]**, a 3 mm diameter glassy carbon electrodes (GCE, BASi, CHI104) was used as the working electrode, and Pt was used as the counter electrode. Electrodes were polished with an alumina slurry (0.05 μm) on a wet polishing cloth to ensure their optimal performance before each set of experiments. A solution of **[(Biot-gly)$_2$Fe$_4$S$_4$]** in DMF (10 mM stock solution, final concentration 200 μM) was diluted with borate buffer (100 mM, pH 8.2) containing $KPF_6$ (100 mM) to a total volume of 2 mL. The scan rate

for the experiments was 1 V·s$^{-1}$ unless otherwise noted. Scan rate-dependent cyclic voltammograms were recorded to ensure the integrity of the cluster after reduction. The half-wave potentials ($E_{1/2}$) were defined as the weighing average of the corresponding cathodic peak potential ($E_c$) and anodic peak potential ($E_a$) for reversible processes. For irreversible processes, the potential at which half of the peak current ($I_c/2$) was reached, was set as $E_{1/2}$. A similar method was applied for the CV experiments with **[(Biot-β-ala)$_2$Fe$_4$S$_4$].**

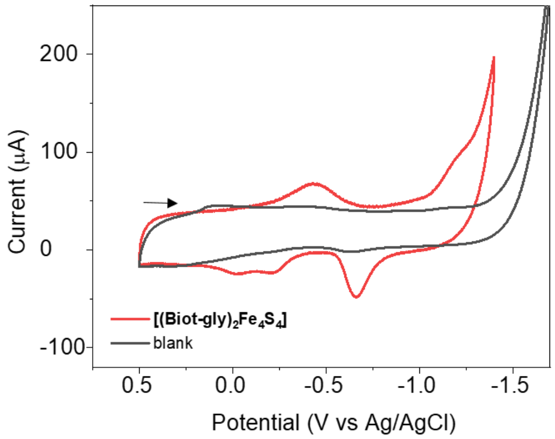


**Figure S9.** Cyclic voltammogram of **[(Biot-gly)$_2$Fe$_4$S$_4$]** (red trace) and the blank (black trace) (identical set-up except for the omission of **[(Biot-gly)$_2$Fe$_4$S$_4$]**). In the blank measurement, two small peaks are observed: one at around 0.1 V (cathodic scan) and one at –0.7 V (anodic scan) (performed in triplicate). We hypothesize that the signals arise from the borate buffer. However, the current densities of these two peaks are significantly lower than the current density observed for **[(Biot-gly)$_2$Fe$_4$S$_4$]**.

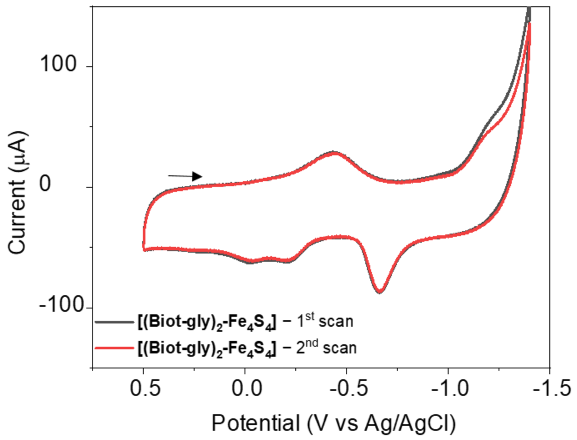


**Figure S10.** Cyclic voltammogram of **[(Biot-gly)$_2$Fe$_4$S$_4$]** (two consecutive scans).

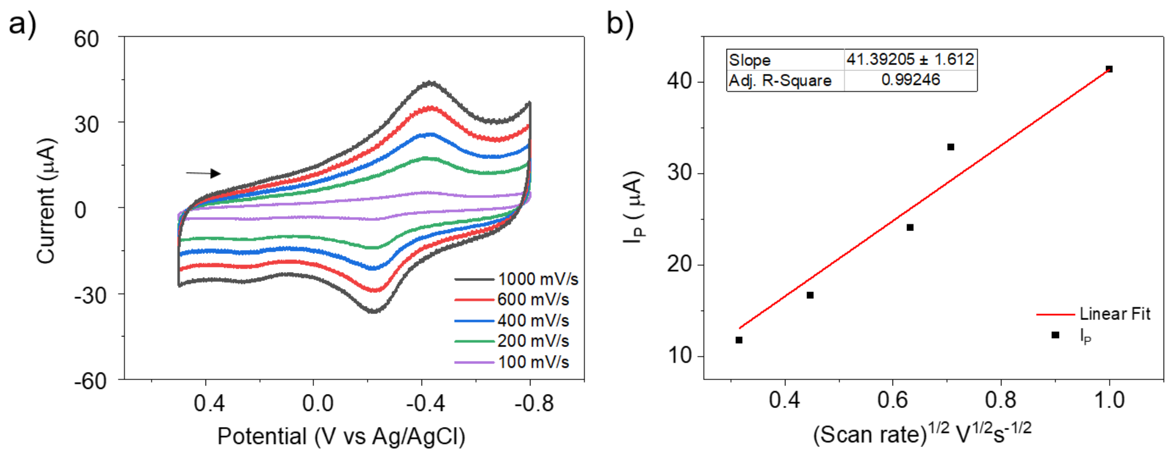


**Figure S11.** Scan rate-dependent cyclic voltammograms. a) Cyclic voltammogram of **[(Biot-gly)$_2$Fe$_4$S$_4$]** at different scan rates. b) Plot of peak current ($I_P$) vs square root of scan rate and the corresponding linear fit.

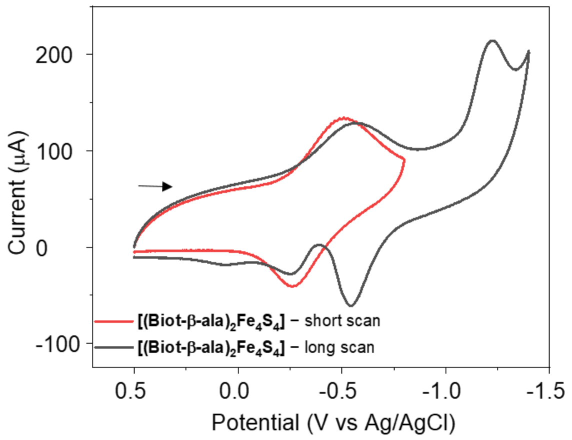


**Figure S12.** Cyclic voltammogram of **[(Biot-β-ala)$_2$Fe$_4$S$_4$]**.

### IV.2) Electrochemical Analysis of [(Biot-gly)$_2$Fe$_4$S$_4$]·Sav Isoforms and [(Biot-β-ala)$_2$Fe$_4$S$_4$]·Sav AA

A 5 mm diameter gold electrode (ALS Co. Ltd) was polished with a wet polishing cloth using an alumina slurry (0.05 µm) and cleaned electrochemically by sweeping several times between 1.7 V and −0.3 V (vs Ag/AgCl reference electrode) in $H_2SO_4$ (0.5 M). A solution of L-cysteine in Milli-Q water (2 mM) was prepared. The cleaned and dried electrode was immersed in this solution overnight to afford the L-cysteine modified gold electrode. Under an inert atmosphere, a solution of **[(Biot-gly)$_2$Fe$_4$S$_4$]** in DMF (10 mM stock solution, final concentration 250 µM) was added to a solution of Sav isoforms (250 µM) in a borate buffer (100 mM, pH 8.2, total volume 400 µL), and the mixture was incubated for 30 min. Note that, an excess of Sav was added to ensure the quantitative incorporation of **[(Biot-gly)$_2$Fe$_4$S$_4$]** in Sav. This solution was then centrifuged and the modified gold electrode was immersed in this solution for 3 hours under strictly anaerobic conditions. After that, the surfaces were thoroughly rinsed with Milli-Q water and the electrode was used as the working electrode. The same reference and counter electrodes were used as described in section IV.1. As electrolyte solution a borate buffer (100 mM, pH 8.2, 2 mL total volume) containing $KPF_6$ (100 mM) was

used. The rest of the protocol was identical to section IV.1. A similar method was used to conduct the CV experiments of **[(Biot-β-ala)$_2$Fe$_4$S$_4$]**·Sav AA.

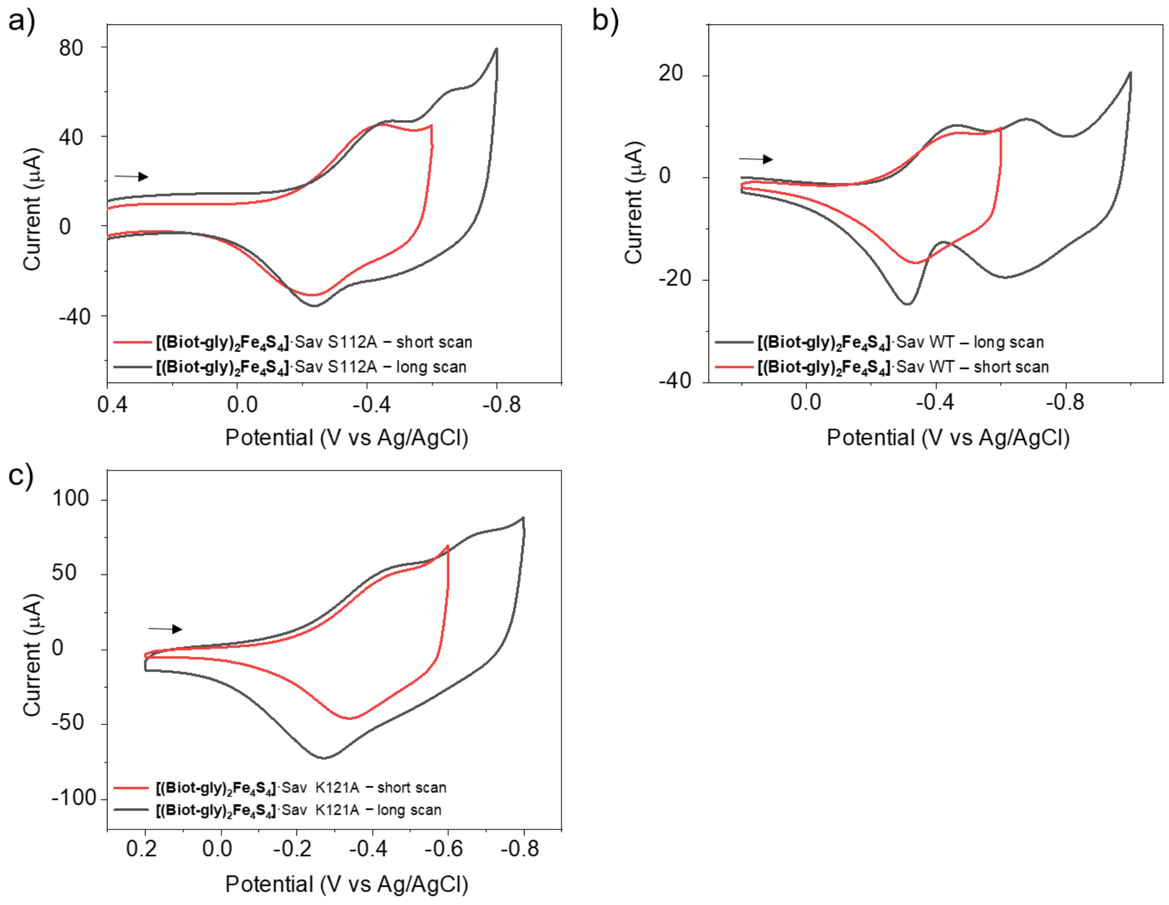


**Figure S13.** Cyclic voltammograms of different mutants of **[(Biot-gly)$_2$Fe$_4$S$_4$]**·Sav. a) S112A. b) Sav WT. c) Sav K121A.

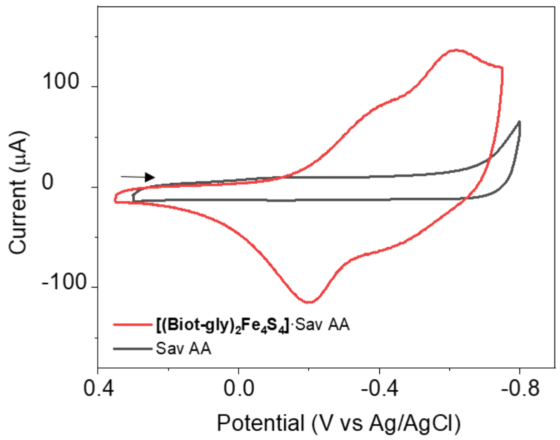


**Figure S14.** Cyclic voltammograms of **[(Biot-gly)$_2$Fe$_4$S$_4$]**·Sav AA (red trace) and apo Sav AA (black trace) (identical set-up except for the omission of **[(Biot-gly)$_2$Fe$_4$S$_4$]**).

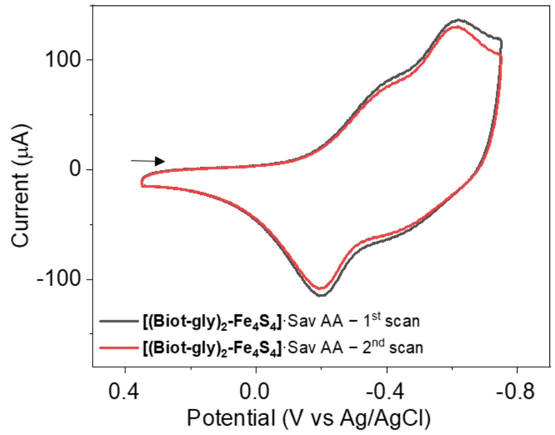


**Figure S15.** Cyclic voltammograms of **[(Biot-gly)$_2$Fe$_4$S$_4$]**·Sav AA (two consecutive scans).

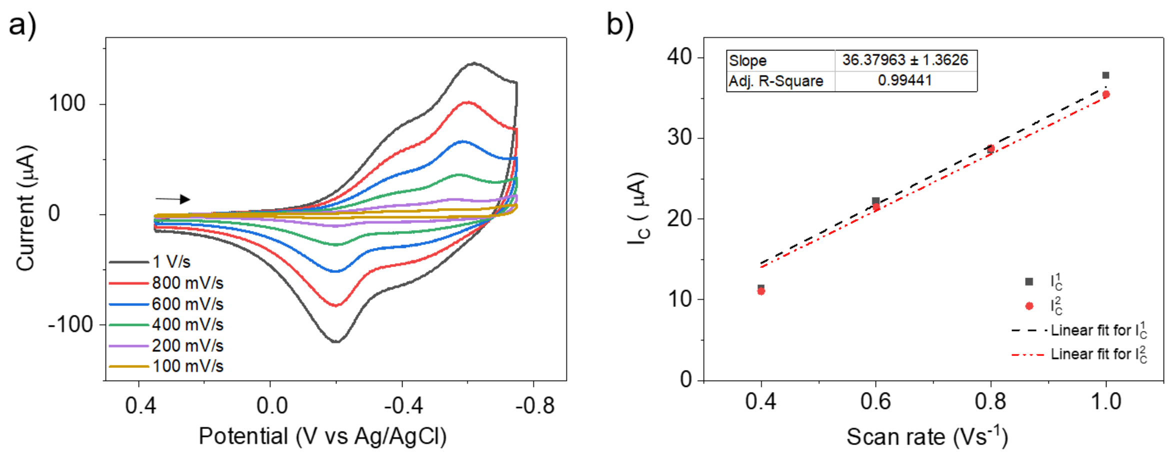


**Figure S16.** Scan rate-dependent cyclic voltammograms. a) Cyclic voltammogram of **[(Biot-gly)$_2$Fe$_4$S$_4$]**·Sav AA at different scan rates. b) Plot of peak currents of the [Fe$_4$S$_4$]$^{2+/1+}$-redox event ($I_C^1$) respectively the [Fe$_4$S$_4$]$^{1+/0}$-redox event ($I_C^2$) vs scan rate and the corresponding linear fits.

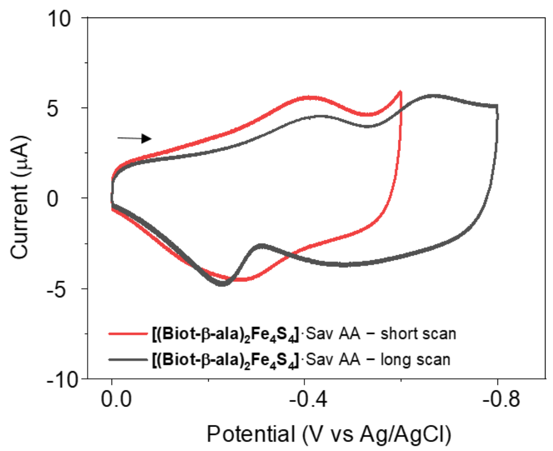


**Figure S17.** Cyclic voltammogram of **[(Biot-β-ala)$_2$Fe$_4$S$_4$]**·Sav AA.

### IV.3) Electrochemical Analysis of [(Biot-gly)$_2$Fe$_4$S$_4$], Biot·Sav

Sav AA (250 µM) was incubated with biotin (10 mM) in a borate buffer (100 mM, pH 8.2) for 30 minutes (Biot·Sav hereafter). To 400 µL of this solution, was added a

solution of **[(Biot-gly)$_2$Fe$_4$S$_4$]** in DMF (10 mM stock solution, final concentration 250 µM), and the mixture was incubated for 30 min. This solution was then centrifuged. A gold electrode, which had been modified with L-cystein (see section IV.2), was immersed in this solution for 3 hours under strictly anaerobic conditions. After that, the surfaces were thoroughly rinsed with Milli-Q water and the electrode was used as working electrode. The rest of the protocol was identical to section IV.2.

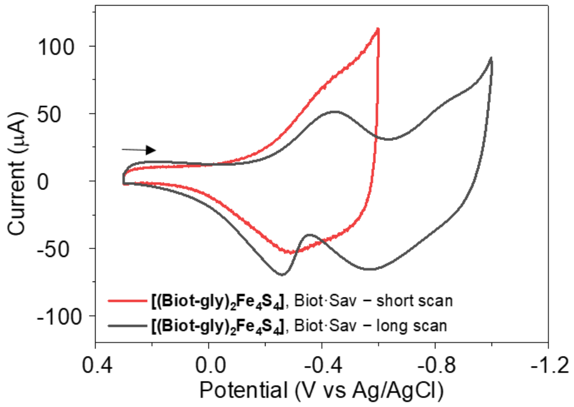


**Figure S18.** Cyclic voltammogram of **[(Biot-gly)$_2$Fe$_4$S$_4$]** in the presence of Biot·Sav.

### IV.4) Electrochemical Analysis of [(Biot-gly)$_2$Fe$_4$S$_4$], BSA

A solution of **[(Biot-gly)$_2$Fe$_4$S$_4$]** in DMF (10 mM stock solution, final concentration 250 µM) was added to a solution of BSA (1 mM) in borate buffer (100 mM, pH 8.2), and the mixture was incubated for 30 min. The rest of the protocol was identical to section IV.3, except that differential pulse voltammetry (DPV) was conducted instead of CV.

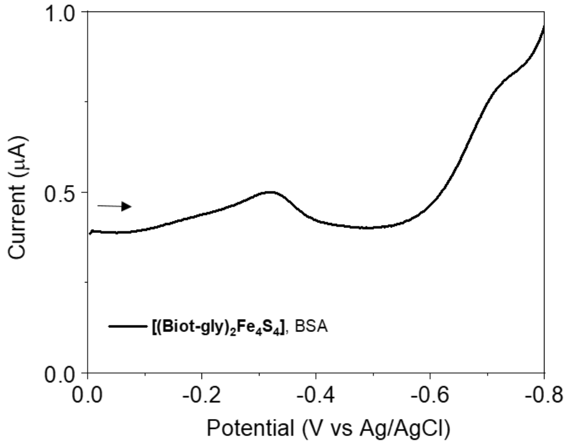


**Figure S19.** DPV of **[(Biot-gly)$_2$Fe$_4$S$_4$]** in the presence of BSA.

## IV.5) CV Summary

**Table S1.** Redox potentials determined for **[(Biot-gly)$_2$Fe$_4$S$_4$]**·Sav mutants (in triplicate with standard deviations).

| | **E$_1$** | **E$_2$** |
|---|---|---|
| no protein | −(343 ±15) mV | −(1014 ± 20) mV |
| Sav AA | −(305 ± 5) mV | −(514 ± 13) mV |
| Sav WT | −(390 ± 5) mV | −(637 ± 5) mV |
| Sav K121A | −(391 ± 9) mV | −(599 ± 4 ) mV |
| Sav S112A | −(322 ± 12) mV | −(590 ± 14) mV |
| Biot·Sav | −349 mV | −708 mV |
| BSA | −324 mV | −724 mV |

# V) Modelling Studies

Sav WT (PDB 6M9B) was processed with the PDB2PQR server to assign the missing H atoms at pH 7.4.[6] Next, the structure of **[(Biot-gly)$_2$Fe$_4$S$_4$]**·Sav WT was computed using the 2-layer ONIOM method implemented Gaussian09.[7] The calculations were performed at the ONIOM(uBP86/6-31G(d):Amber) level, without the inclusion of any symmetry constraints. The high layer included the [Fe$_4$S$_4$] core, the dithiolate ligand, and the glycine spacer (up to the amide bond between biotin and glycine). For the low layer, the catalyst's atomic charges (RESP) were calculated at the same level as the high layer. The force-field used for the catalyst was determined according to references.[8-9] The estimation of the initial orbital in Gaussian09 was generated according to the reference.[10] During the energy minimization process, only atoms within 8 Å of the cofactor **[(Biot-gly)$_2$Fe$_4$S$_4$]** were allowed to relax.

# VI) Catalysis

### VI.1) $CO_2$ Reduction Assay

**[(Biot-gly)$_2$Fe$_4$S$_4$]:** A solution of **[(Biot-gly)$_2$Fe$_4$S$_4$]** in DMF (5 mM stock solution, final concentration 5 µM) was placed in a GC vial (9.4 mL total volume) and closed with a crimped rubber cap inside of the glovebox. A $NaHCO_3$ solution (0.5 M) was purged with $CO_2$ for 3 h. This solution (0.8 mL) was added to the vials via a syringe. The headspace of the GC vials was purged with $CO_2$ for 1 min per sample. The solutions were then left to stand for 10 min. A stock solution of Eu(II)-DTPA (200 mM) was prepared by dissolving DTPA (79 mg, 0.2 mmol), NaOH (40 mg, 1 mmol) and $EuBr_2$ (62 mg 0.2 mmol) in $H_2O$ (1 mL). Aliquots of this solution were added to each vial via a syringe (0.2 mL). The vials were shaken in an incubator at 25 °C for two days. The

pH of the reaction mixture was 7.5 ± 0.1. **[(Biot-gly)$_2$Fe$_4$S$_4$]**·Sav mutants: A solution of Sav isoforms in $H_2O$ (250 μM stock solution, final concentration 5 μM) was added to the vials before closing the crimp cap. **[(Biot-gly)$_2$Fe$_4$S$_4$],** Biot·Sav: A solution of Sav AA in $H_2O$ (250 μM stock solution) was incubated with 100 equivalents of biotin (1 M stock solution dissolved in $H_2O$ with minimal amount of $NaHCO_3$ for dissolution) at 25 °C for 1 h. Aliquots of this solution (20 µL) were added to the vials before closing the crimp cap. **[(Biot-gly)$_2$Fe$_4$S$_4$]**, BSA: A solution of BSA in $H_2O$ (16 mg/mL stock solution, final concentration 0.3 mg/mL) was added to the vials before closing the crimp cap. **$FeCl_3$/$Na_2S$:** Stock solutions of $FeCl_3$ in $H_2O$ (20 mM), $Na_2S$ in $H_2O$ (20 mM) and **(Biot-gly**) in DMF (10 mM) were prepared. The solutions were placed in the GC vials instead of **[(Biot-gly)$_2$Fe$_4$S$_4$]**) (in equimolar amounts) before closing the crimp cap. A similar method was used for $CO_2$ reduction assay with **[(Biot-β-ala)$_2$Fe$_4$S$_4$]** and **[(Biot-β-ala)$_2$Fe$_4$S$_4$]**·Sav isoforms.

### VI.2) $CO_2$ Reduction in Phosphate Buffer

**[(Biot-gly)$_2$Fe$_4$S$_4$]:** A solution of **[(Biot-gly)$_2$Fe$_4$S$_4$]** in DMF (5 mM stock solution, final concentration 5 µM) was placed in a GC vial (9.4 mL) and a $NaHCO_3$ solution (0.74 M, 700 µL thereof) was added. Then the vials were closed with a crimped rubber cap and a $HNa_2PO_4$ solution (2 M, 100 µL thereof) was added via a syringe. The solutions were then left to stand for 10 min. The rest of the protocol was identical to section VII.1. The pH of the reaction mixture was 7.6 ± 0.1. **No $CO_2$:** A solution of **[(Biot-gly)$_2$Fe$_4$S$_4$]** in DMF (5 mM stock solution, final concentration 5 µM) was placed in a GC vial (9.4 mL) and a phosphate buffer (0.25 M, pH 7.5, 800 µL thereof) was added. The solutions were then left to stand for 10 min. The rest of the protocol was identical to section VII.1. The pH of the reaction mixture was 7.60 ± 0.1. **[(Biot-gly)$_2$Fe$_4$S$_4$]**·Sav AA**:** A solution

of Sav AA in $H_2O$ (250 µM stock solution, final concentration 5 µM) was added to the vials before closing the crimp cap.

### VI.3) $CO_2$ Reduction with $^{13}CO_3HNa$ or $^{12}CO_3HNa$

**[(Biot-gly)$_2$Fe$_4$S$_4$]:** A solution of **[(Biot-gly)$_2$Fe$_4$S$_4$]** in DMF (5 mM stock solution, final concentration 5 µM) was placed in a GC vial (9.4 mL total volume) and a $NaHCO_3$ solution ($^{13}CO_3HNa$ or $^{12}CO_3HNa$) (0.74 M, 700 µL thereof) was added. Then, the vials were closed with a crimped rubber cap and a $Na_2HPO_4$ solution (2 M, 100 µL thereof) was added via a syringe. The solutions were then left to stand for 10 min. The rest of the protocol was identical to section VII.1. The pH of the reaction mixture was 7.6 ± 0.1. **[(Biot-gly)$_2$Fe$_4$S$_4$]**·Sav AA: A solution of Sav AA in $H_2O$ (250 µM stock solution, final concentration 5 µM) was added to the vials before sealing the crimp cap.

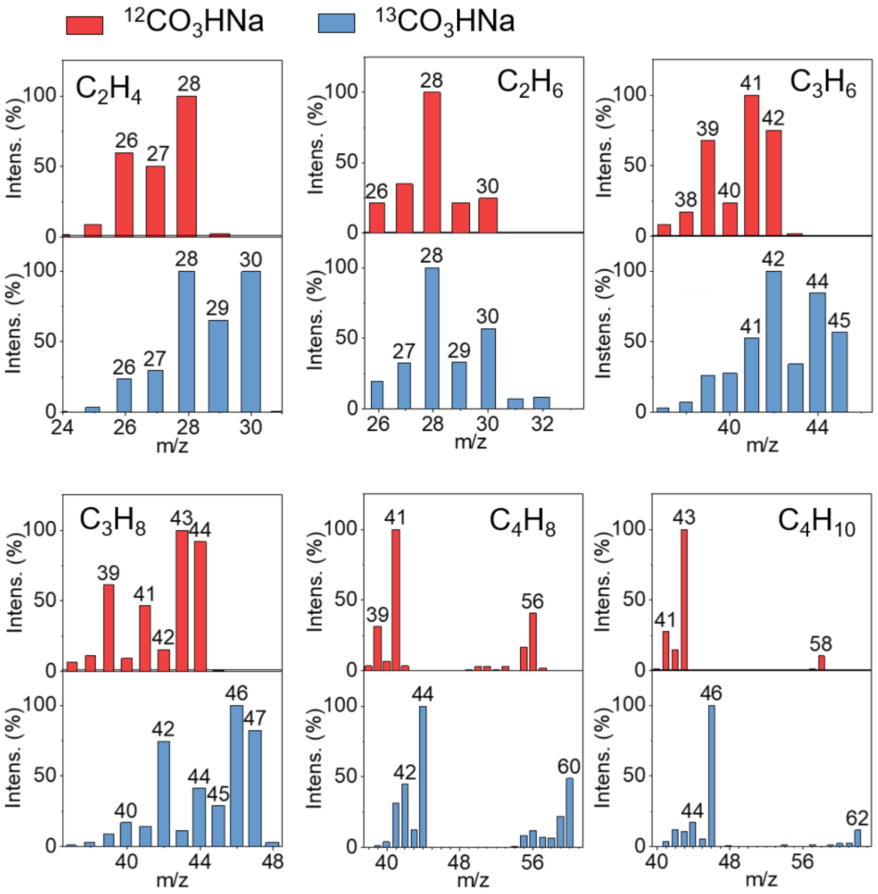


**Figure S20.** Fragmentation pattern of hydrocarbon products generated from the reduction of $^{13}CO_3HNa$ and $^{12}CO_3HNa$ respectively in the presence of **$[(Biot\text{-}gly)_2Fe_4S_4]$**·Sav AA.

### VI.4) Kinetics of $CO_2$ Reduction

The samples were prepared as described in section VII.1. The headspace samples were taken manually with a gastight syringe (250 µL each). Separate samples were used for the detection of $H_2$ (SRI GC) and $C_nH_m$ (Agilent GC). The results of three independent runs were averaged and the values fitted to first-order kinetics with the least-squares method, Equation 1.

$$S(t) = A\left(1 - e^{-\left(\frac{t}{\tau}\right)}\right) + B \qquad (1)$$

$\tau$: time constant; A: amplitude of reaction, B: Baseline

**Table S2.** Fitted parameters (Figure 5c).

| | | $C_nH_m$ | $H_2$ |
|---|---|---|---|
| **[(Biot-gly)$_2$Fe$_4$S$_4$]** | τ (min) | 33.4 ± 3.7 | 39.2 ± 12.4 |
| | A | 1.7 ± 0.1 | 4.1 ± 0.4 |
| | B | 0.0 ± 0.1 | 0.0 ± 0.4 |
| | $R^2$ | 99.8% | 98.4% |
| **[(Biot-gly)$_2$Fe$_4$S$_4$]**·Sav AA | τ (h) | 22.5 ± 3.8 | 15.9 ± 2.3 |
| | A | 7.1 ± 0.4 | 3.1 ± 0.1 |
| | B | 0.6 ± 0.2 | 0.8 ± 0.1 |
| | $R^2$ | 99.2% | 99.2% |
| **[(Biot-gly)$_2$Fe$_4$S$_4$]**, Biot·Sav | τ (min) | 209 ± 45 | 136 ± 20 |
| | A | 4.7 ± 0.4 | 3.8 ± 0.2 |
| | B | 0.0 ± 0.2 | 0.0 ± 0.1 |
| | $R^2$ | 98.9% | 99.1% |

### VI.5) Addition of Multiple Eu(II)-DTPA Aliquots

The samples were prepared as described in section VII.1. After completion of the reaction (evidenced by a color change from yellow to transparent after consumption of Eu(II)-DPTA), a headspace sample was taken with a gastight syringe (250 µL each), and analyzed by GC-FID (Agilent). An additional aliquot of the Eu(II)-DTPA solution (200 mM, 0.2 mL thereof) was added, and the sample was shaken again until completion of the reaction. The procedure was repeated as often as revealed in Figure S21.

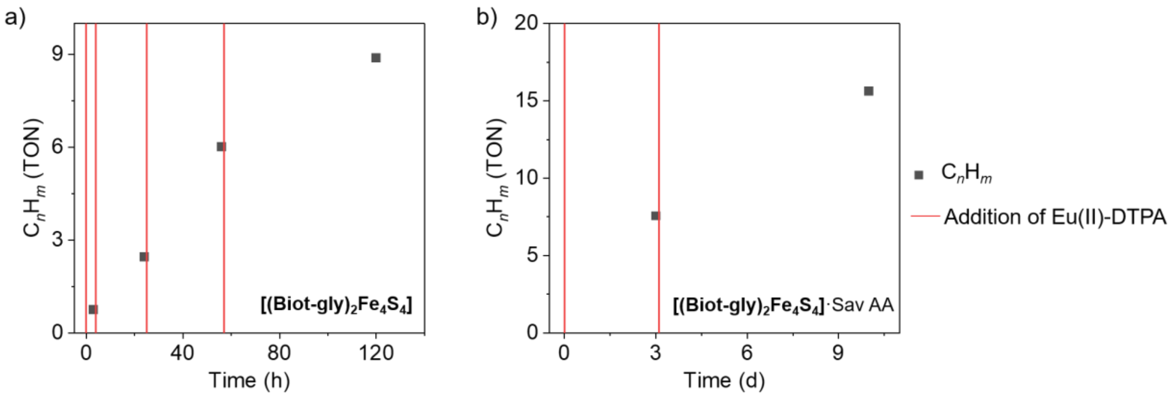


**Figure S21.** $CO_2$ reduction after adding multiple aliquots of Eu(II)-DTPA. a) $C_nH_m$ products detected during $CO_2$ reduction in the presence of **[(Biot-gly)$_2$Fe$_4$S$_4$]**. b) $C_nH_m$ products detected during $CO_2$ reduction in the presence of **[(Biot-gly)$_2$Fe$_4$S$_4$]**·Sav AA. We hypothesize that the erosion of catalytic activity is due to the rise of pH resulting from repeated addition of highly basic Eu(II)-DPTA.

# VII) GC Traces and Calibration

## VII.1) GC Calibrations

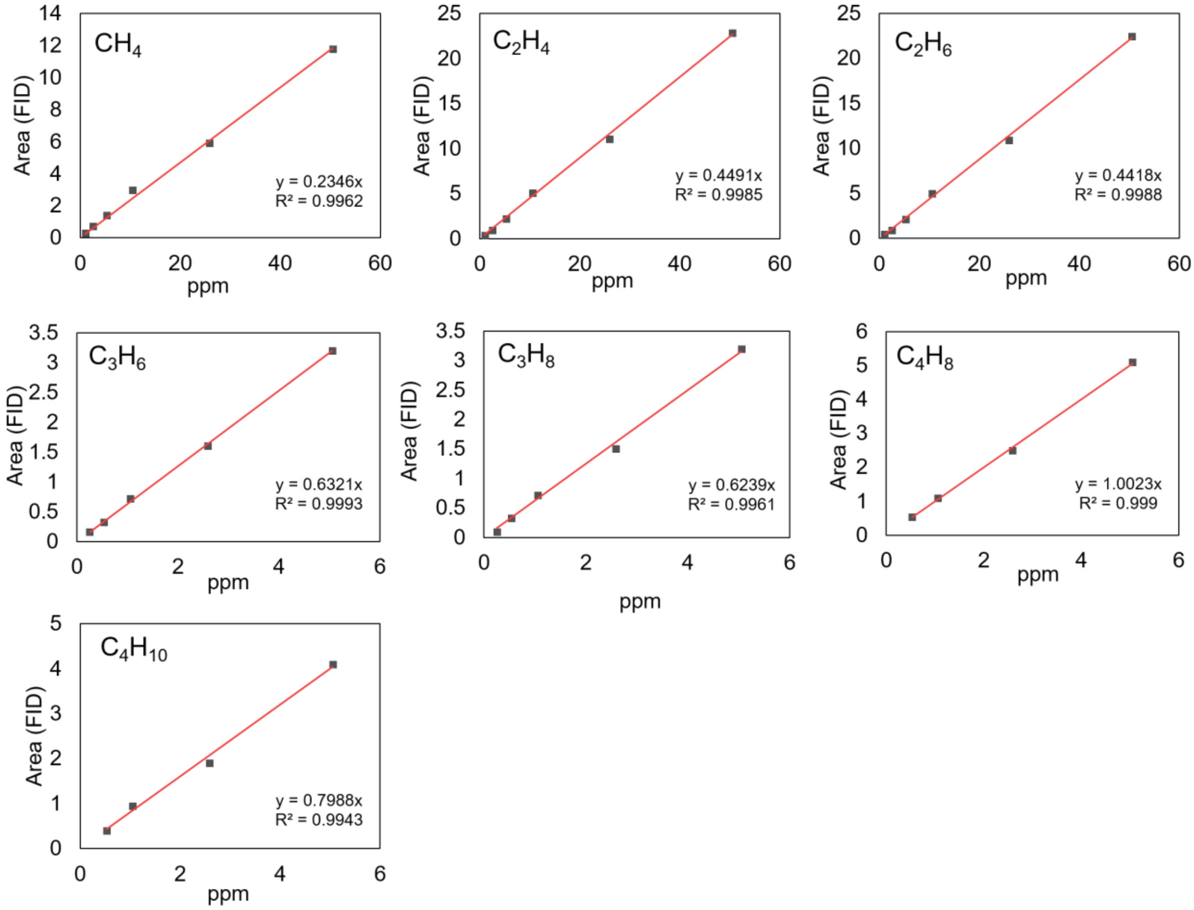


**Figure S22.** Calibration of Agilent GC.

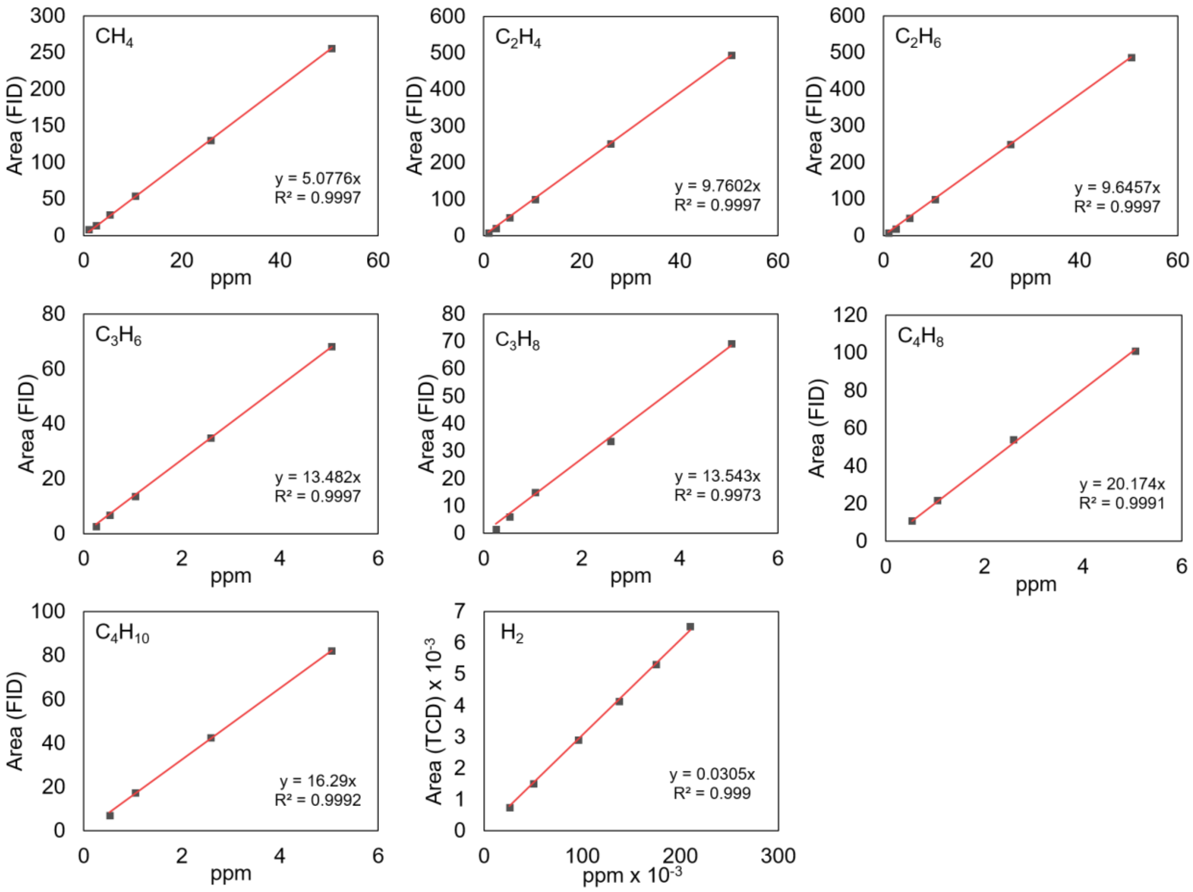


**Figure S23.** Calibration of SRI GC.

## VII.2) GC Traces

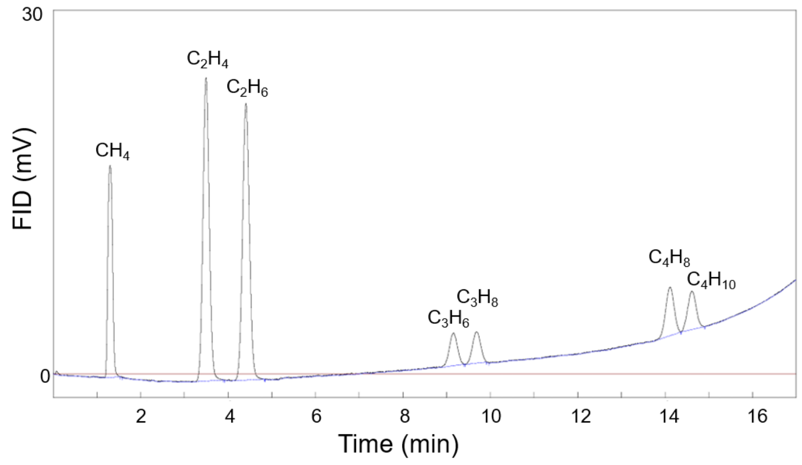


**Figure S24.** SRI GC-FID chromatogram of commercial $C_1$−$C_4$ mixture.

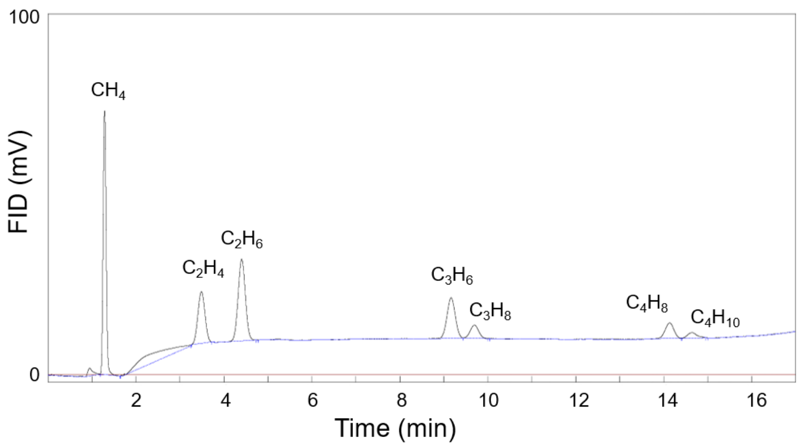


**Figure S25.** SRI GC-FID chromatogram of $CO_2$ reduction with **[(Biot-gly)$_2$Fe$_4$S$_4$]**·Sav AA.

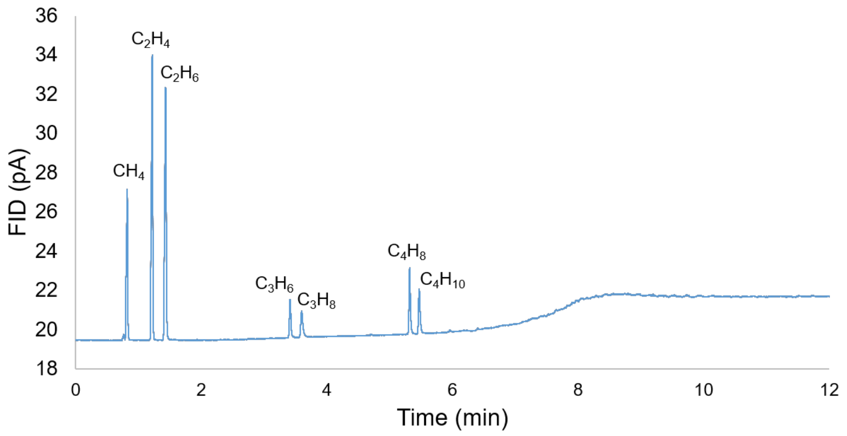


**Figure S26.** Agilent GC-FID chromatogram of commercial $C_1$−$C_4$ mixture.

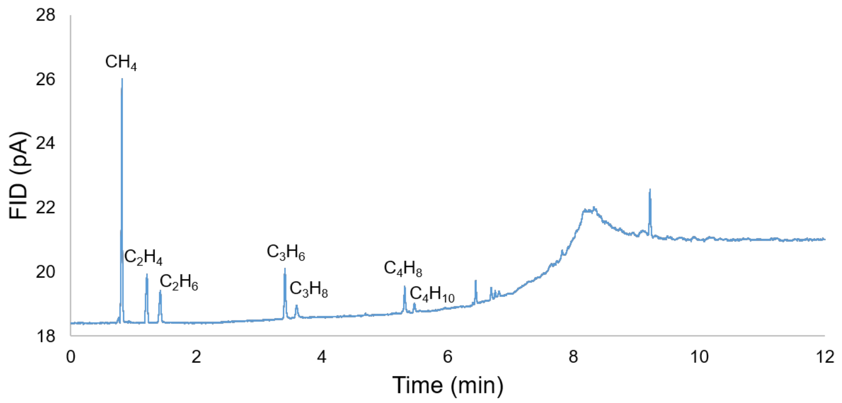


**Figure S27.** Agilent GC-FID chromatogram of $CO_2$ reduction with **[(Biot-gly)$_2$Fe$_4$S$_4$]**·Sav AA.

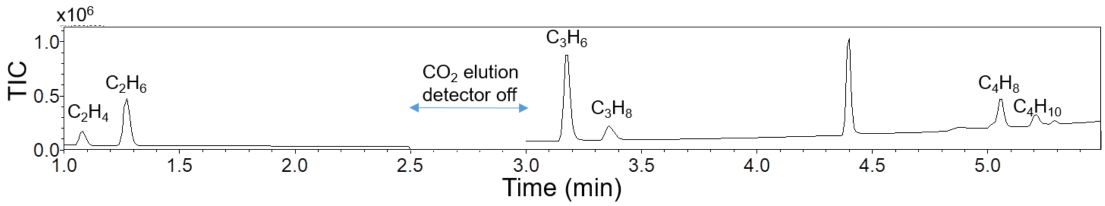


**Figure S28.** GC-MS chromatogram of $^{12}CO_2$ Reduction. $CH_4$ could not be detected since it elutes with the solvent window from min [0–1]. Detector shutoff from min [2.5–3] due to $CO_2$ elution.

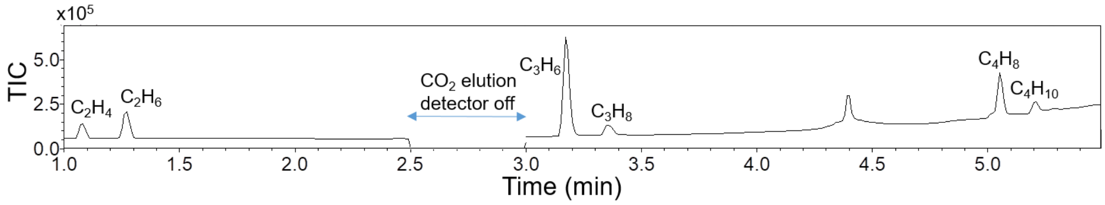


**Figure S29.** GC-MS chromatogram of $^{13}CO_2$ Reduction. $CH_4$ could not be detected since it elutes with the solvent window from min [0–1]. Detector shutoff from min [2.5–3] due to $CO_2$ elution.

## VIII) References


(1) Mallin, H.; Hestericová, M.; Reuter, R.; Ward, T. R., Library design and screening protocol for artificial metalloenzymes based on the biotin-streptavidin technology. *Nat. Protoc.* **2016,** *11* (5), 835-852.

(2) Jafari, M. R.; Yu, H.; Wickware, J. M.; Lin, Y.-S.; Derda, R., Light-responsive bicyclic peptides. *Org. Biomol. Chem.* **2018,** *16* (41), 7588-7594.

(3) Chambers, J. M.; Lindqvist, L. M.; Webb, A.; Huang, D. C. S.; Savage, G. P.; Rizzacasa, M. A., Synthesis of Biotinylated Episilvestrol: Highly Selective Targeting of the Translation Factors eIF4AI/II. *Org. Lett.* **2013,** *15* (6), 1406-1409.

(4) Holm, R. H.; Phillips, W. D.; Averill, B. A.; Mayerle, J. J.; Herskovitz, T., Synthetic analogs of the active sites of iron-sulfur proteins. V. Proton resonance properties of the tetranuclear clusters $[Fe_4S_4(SR)_4]^{2-}$. *J. Am. Chem. Soc.* **1974,** *96* (7), 2109-2117.

(5) DavidáGarner, C., A convenient synthesis of tetrakis[thiolato-$\mu_3$-sulphido-iron](2–) clusters. *J. Chem. Soc., Dalton Trans. (1972-1999)* **1979,** (6), 1093-1094.

(6) Jurrus, E.; Engel, D.; Star, K.; Monson, K.; Brandi, J.; Felberg, L. E.; Brookes, D. H.; Wilson, L.; Chen, J.; Liles, K.; Chun, M.; Li, P.; Gohara, D. W.; Dolinsky, T.; Konecny, R.; Koes, D. R.; Nielsen, J. E.; Head-Gordon, T.; Geng, W.; Krasny, R.; Wei, G.-W.; Holst, M. J.; McCammon, J. A.; Baker, N. A., Improvements to the APBS biomolecular solvation software suite. *Protein Sci.* **2018,** *27* (1), 112-128.

(7) J. Frisch, G. W. T., H. B. Schlegel, G. E. Scuseria, M. A. Robb, J. R. Cheeseman, G. Scalmani, V. Barone, G. A. Petersson, H. Nakatsuji, X. Li, M. Caricato, A. Marenich, J. Bloino, B. G. Janesko, R. Gomperts, B. Mennucci, H. P. Hratchian, J. V. Ortiz, A. F. Izmaylov, J. L. Sonnenberg, D. Williams-Young, F. Ding, F. Lipparini, F. Egidi, J. Goings, B. Peng, A. Petrone, T. Henderson, D. Ranasinghe, V. G. Zakrzewski, J. Gao, N. Rega, G. Zheng, W. Liang, M. Hada, M. Ehara, K. Toyota, R. Fukuda, J. Hasegawa, M. Ishida, T. Nakajima, Y. Honda, O. Kitao, H. Nakai, T. Vreven, K. Throssell, J. A. Montgomery, Jr., J. E. Peralta, F. Ogliaro, M. Bearpark, J. J. Heyd, E. Brothers, K. N. Kudin, V. N. Staroverov, T. Keith, R. Kobayashi, J. Normand, K. Raghavachari, A. Rendell, J. C. Burant, S. S. Iyengar, J. Tomasi, M. Cossi, J. M. Millam, M. Klene, C. Adamo, R. Cammi, J. W. Ochterski, R. L. Martin, K. Morokuma, O. Farkas, J. B. Foresman, and D. J. Fox, *Gaussian 09, Revision E.01*, Gaussian, Inc., Wallingford CT: 2016.

(8) Cornell, W. D.; Cieplak, P.; Bayly, C. I.; Gould, I. R.; Merz, K. M.; Ferguson, D. M.; Spellmeyer, D. C.; Fox, T.; Caldwell, J. W.; Kollman, P. A., A Second Generation Force

Field for the Simulation of Proteins, Nucleic Acids, and Organic Molecules. *J. Am. Chem. Soc.* **1995,** *117* (19), 5179-5197.

(9) Carvalho, A. T. P.; Teixeira, Ana F. S.; Ramos, M. J., Parameters for molecular dynamics simulations of iron-sulfur proteins. *J. Comput. Chem.* **2013,** *34* (18), 1540-1548.

(10) Szilagyi, R. K.; Winslow, M. A., On the accuracy of density functional theory for iron—sulfur clusters. *J. Comput. Chem.* **2006,** *27* (12), 1385-1397.

# IX) HRMS and NMR Spectra

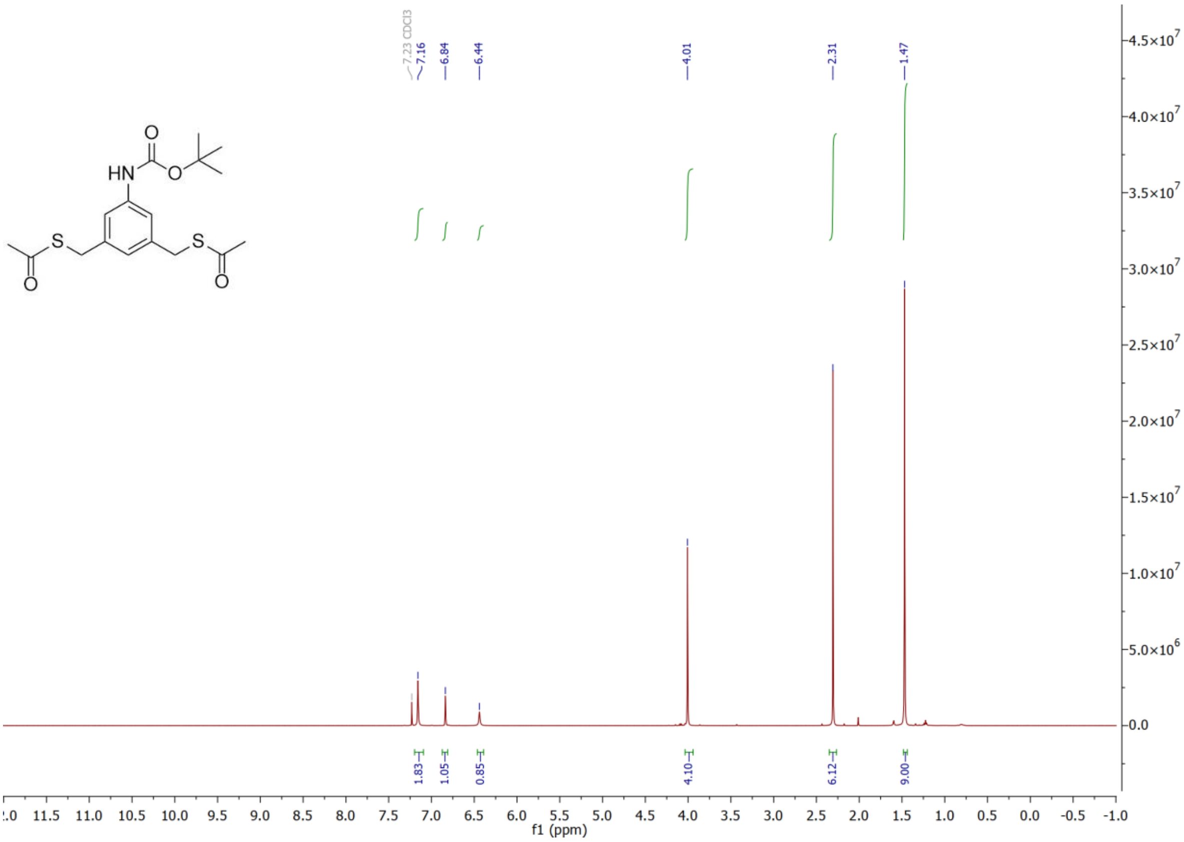


**Spectrum 1.** $^1$H NMR Spectrum of compound **3** in $CDCl_3$.

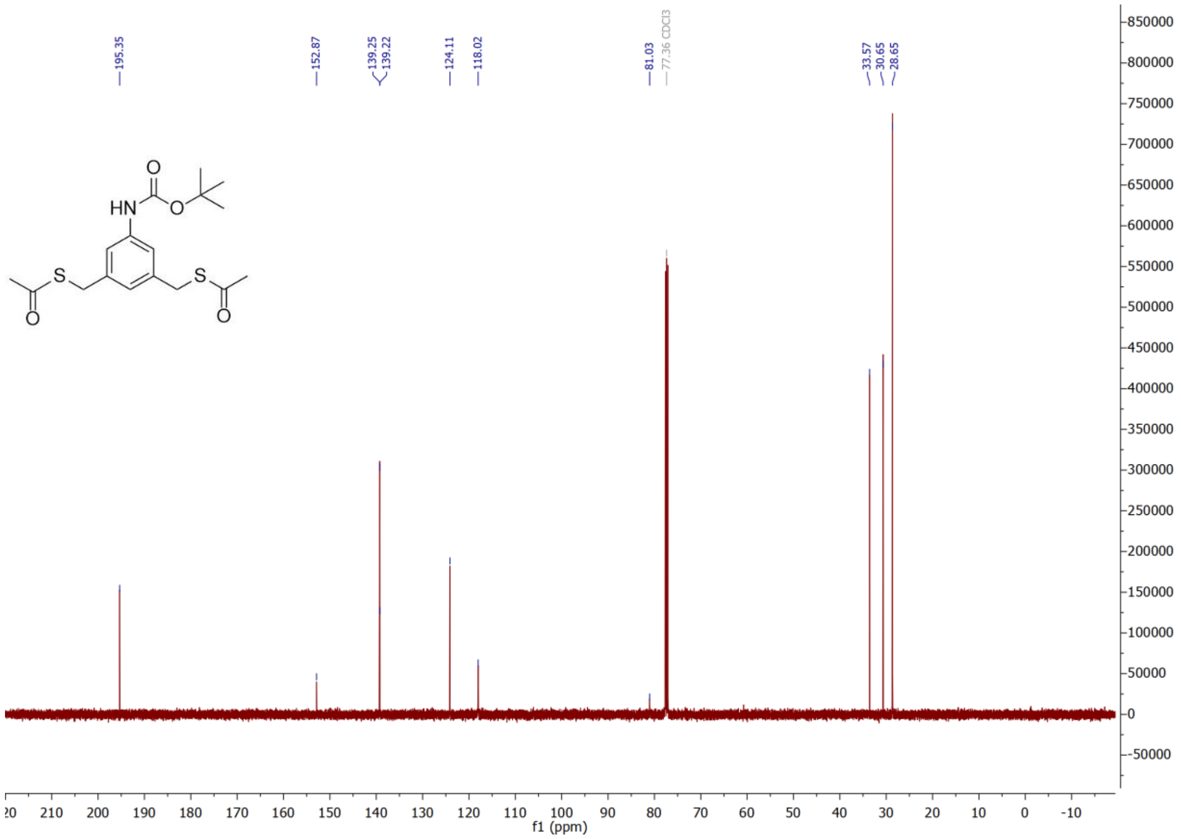


**Spectrum 2.** $^{13}$C NMR Spectrum of compound **3** in $CDCl_3$.

# High Resolution Mass Spectrometry Report

| | | | |
|---|---|---|---|
| Sample Name | **Valerie Waser / VW-70** | Instrument | maXis 4G |
| Comment | 10 ug/mL in ACN/H2O 1:1, analyzed in MeOH | Method | 22 Direct_pos_mid.m |

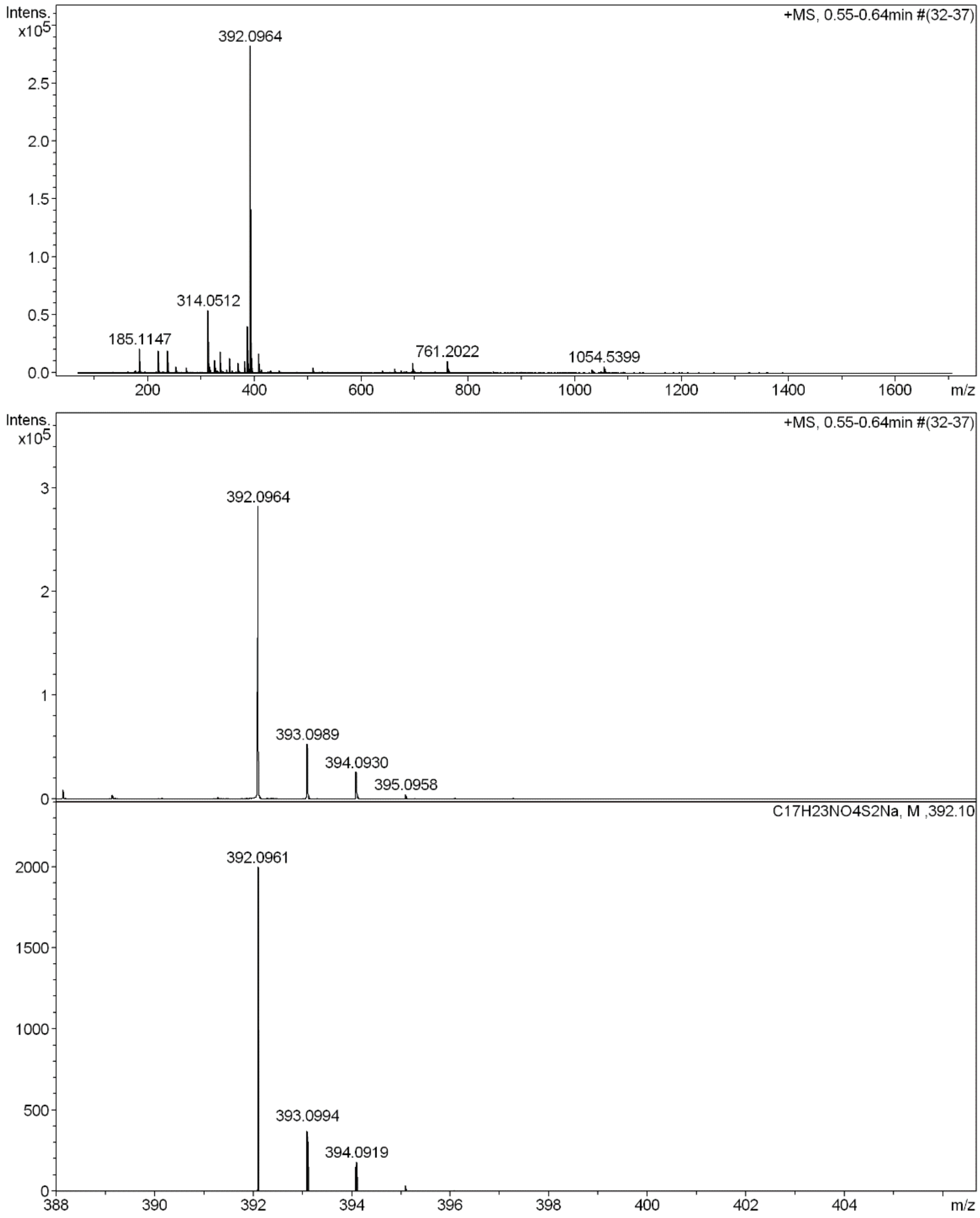


Bruker Compass DataAnalysis 4.0 Acquisition Date 11.06.2019 16:34:33 Page 1 of 3

**Spectrum 3.** HRMS of compound **3**.

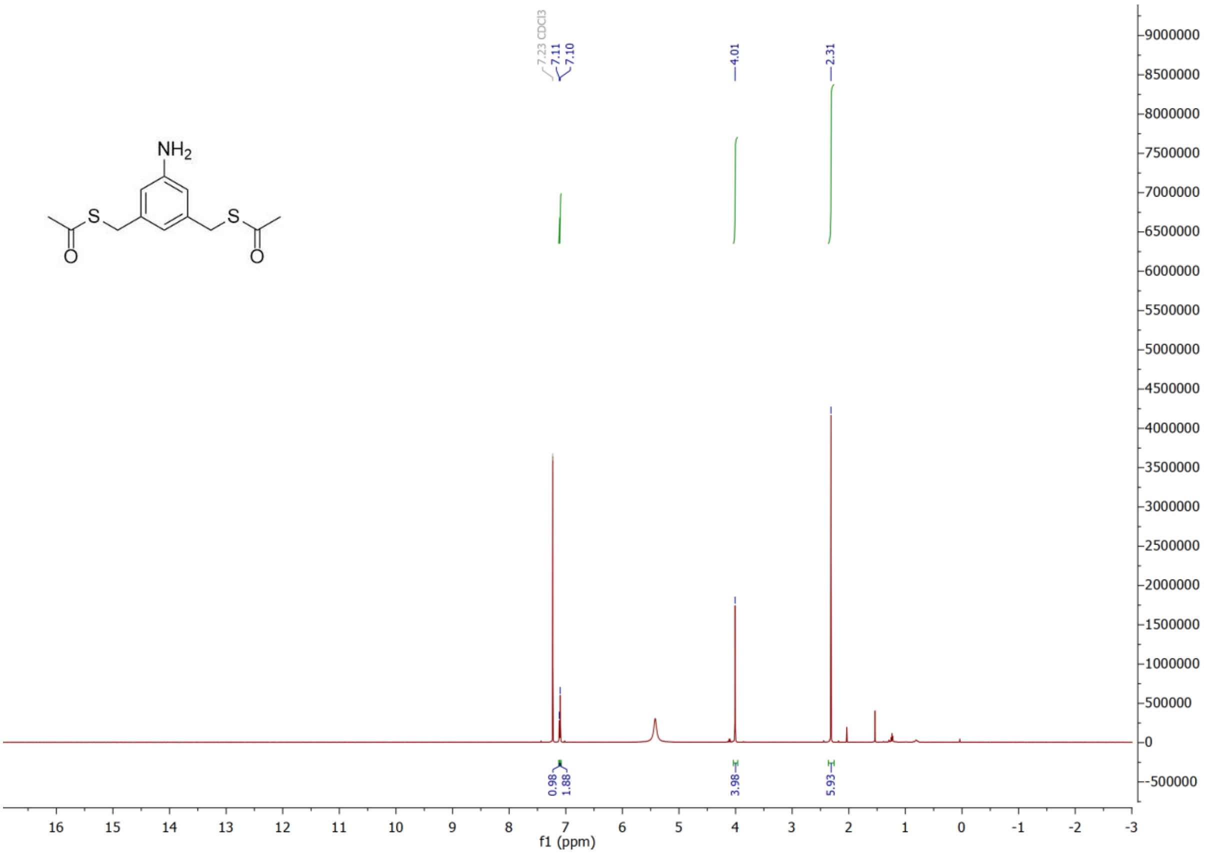


**Spectrum 4.** $^{1}$H NMR Spectrum of intermediate **4a** in $CDCl_3$.

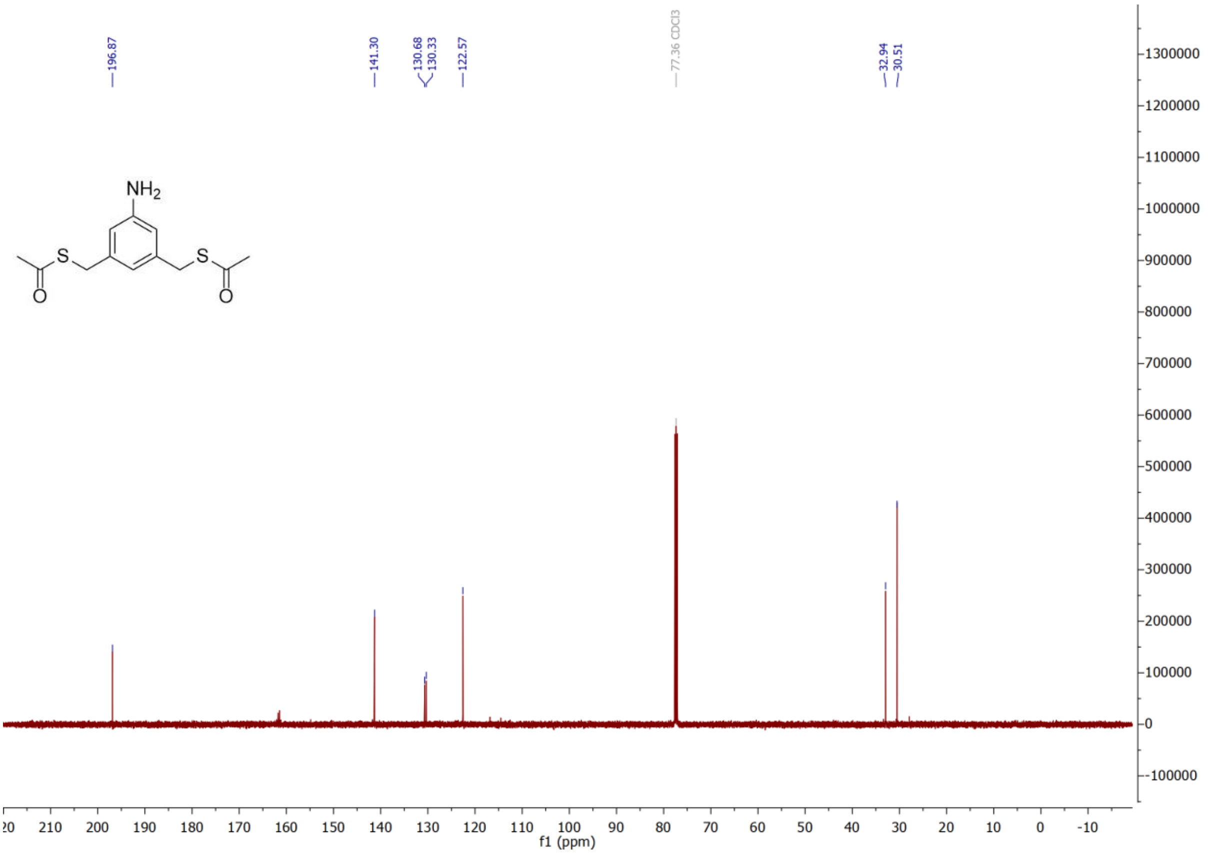


**Spectrum 5.** $^{13}$C NMR Spectrum of intermediate **4a** in $CDCl_3$.

# High Resolution Mass Spectrometry Report

| | | | |
|---|---|---|---|
| Sample Name | **Valerie Waser / VW-72** | Instrument | maXis 4G |
| Comment | 10 ug/mL in ACN/H2O 1:1, analyzed in MeOH | Method | 22 Direct_pos_mid.m |

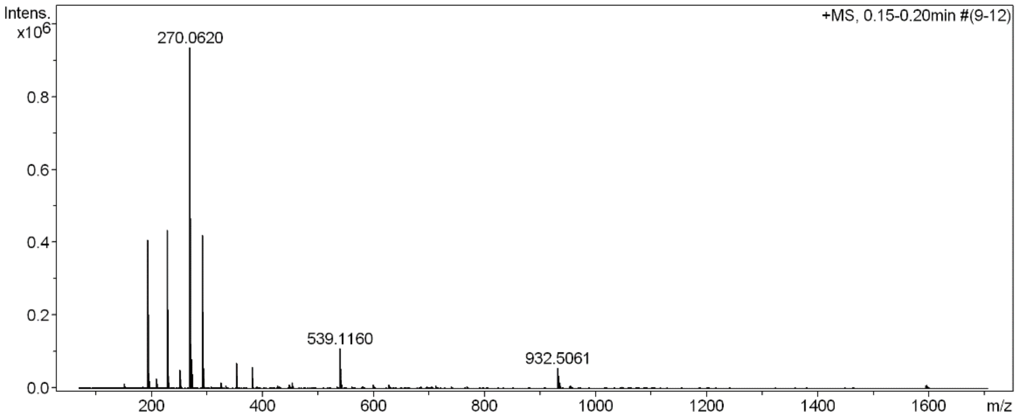


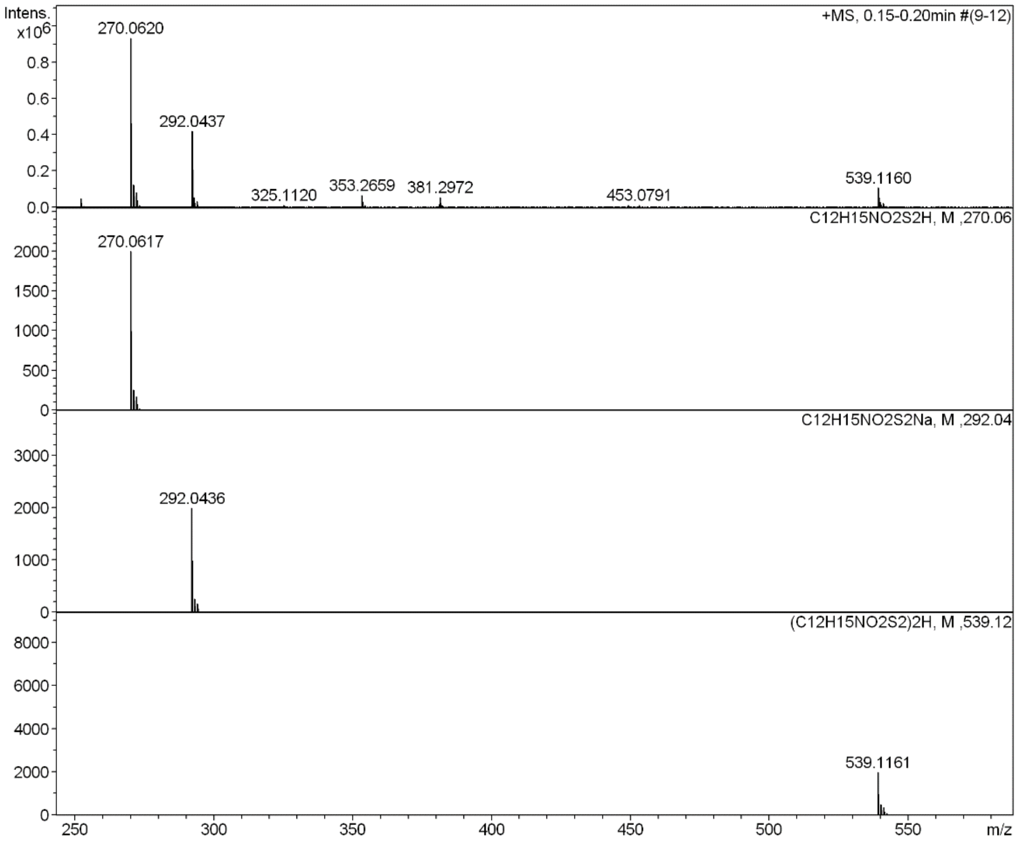


Bruker Compass DataAnalysis 4.0 | Acquisition Date 11.06.2019 16:47:14 | Page 1 of 3

**Spectrum 6.** HRMS of intermediate **4a**.

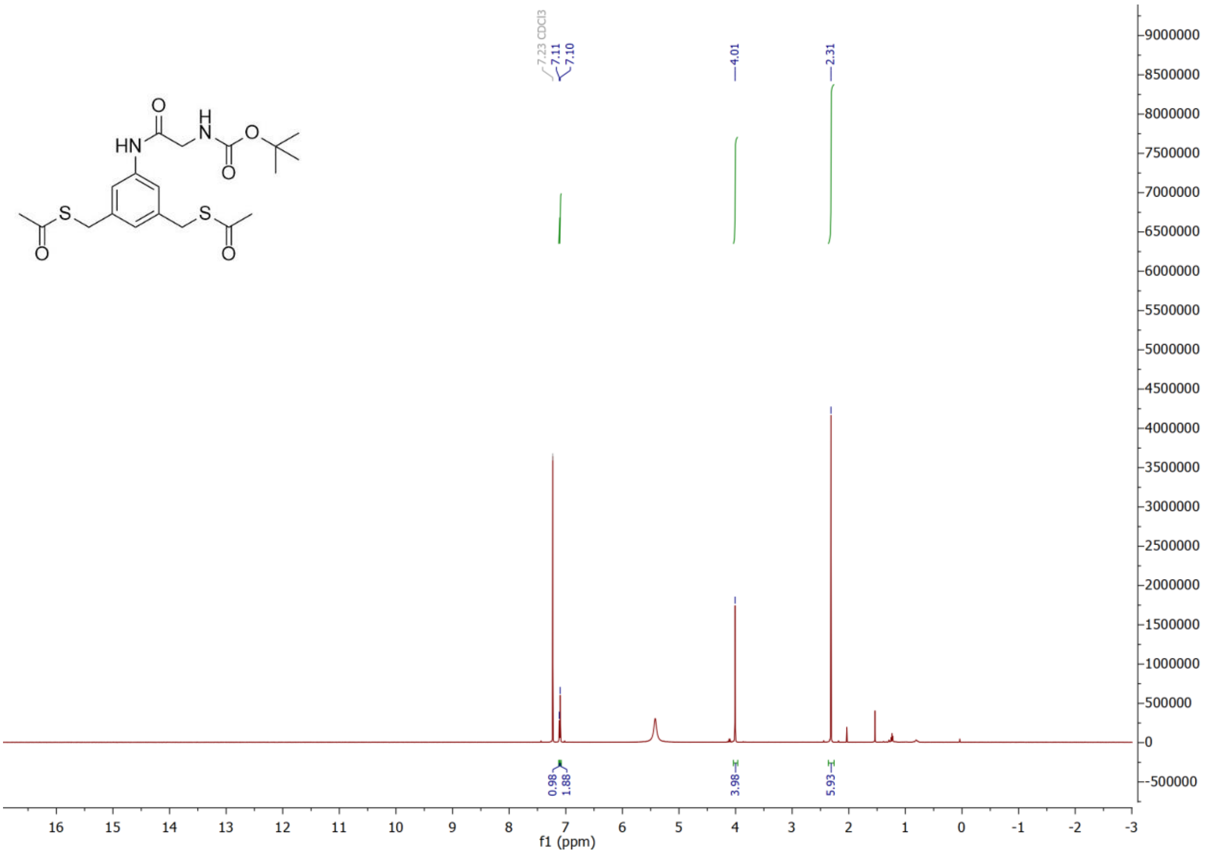


**Spectrum 7.** $^{1}H$ NMR Spectrum of intermediate **4b** in $CDCl_3$.

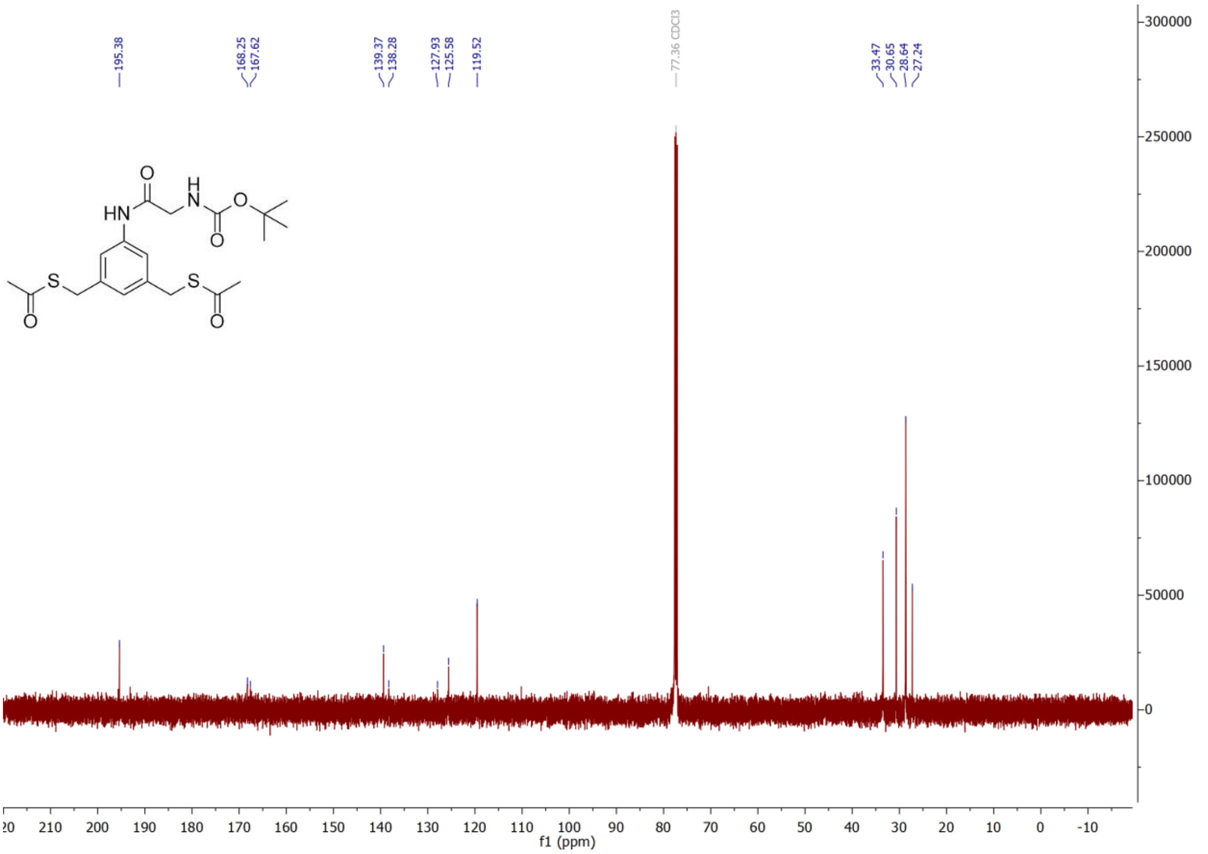


**Spectrum 8.** $^{13}C$ NMR Spectrum of intermediate **4b** in $CDCl_3$.

# High Resolution Mass Spectrometry Report

| | | | |
|---|---|---|---|
| Sample Name | **Valerie Waser / VW-74** | Instrument | maXis 4G |
| Comment | 10 ug/mL in ACN/H2O 1:1, analyzed in MeOH | Method | 22 Direct_pos_mid.m |

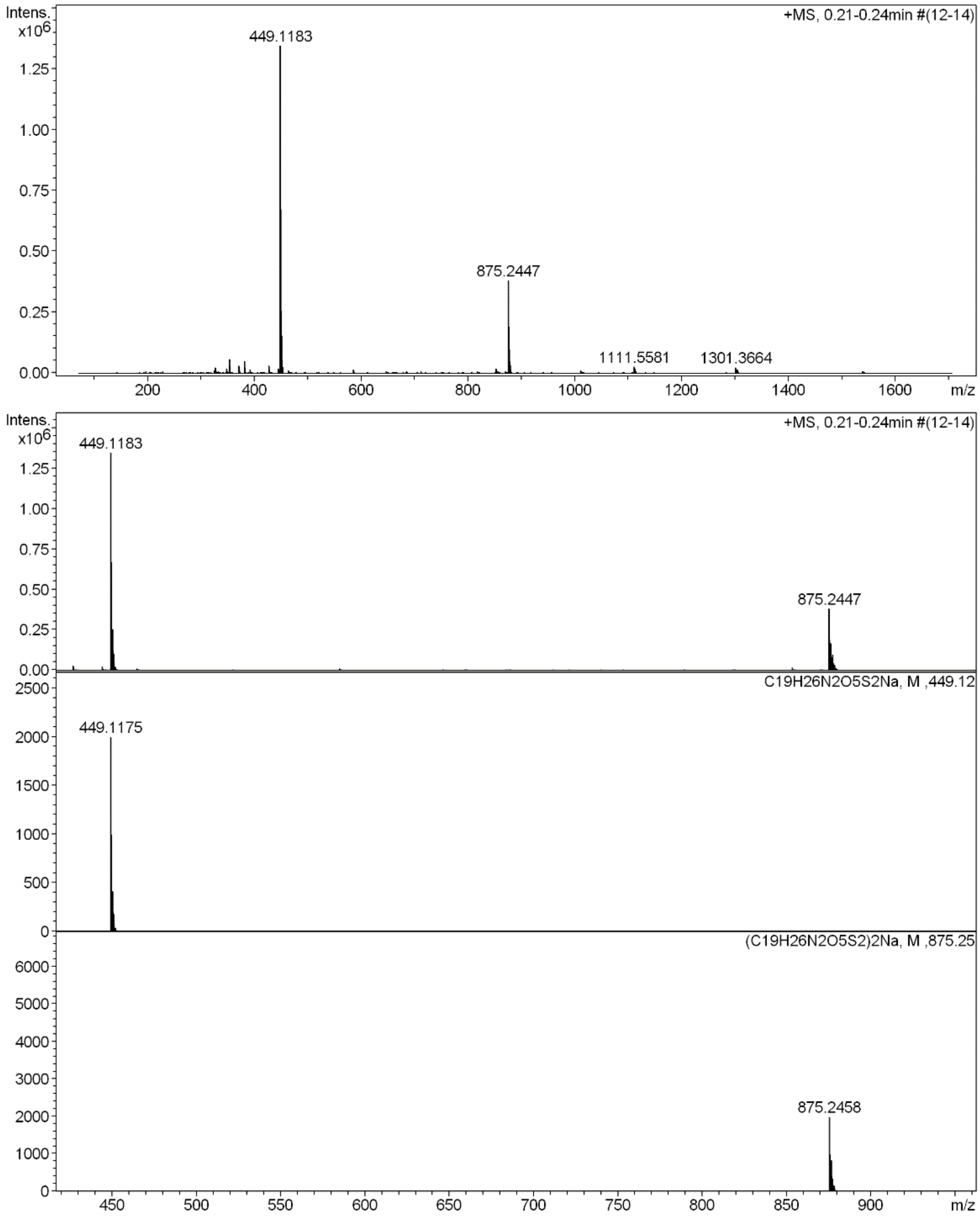


Bruker Compass DataAnalysis 4.0 Acquisition Date 11.06.2019 16:41:54 Page 1 of 3

**Spectrum 9.** HRMS of intermediate **4b**.

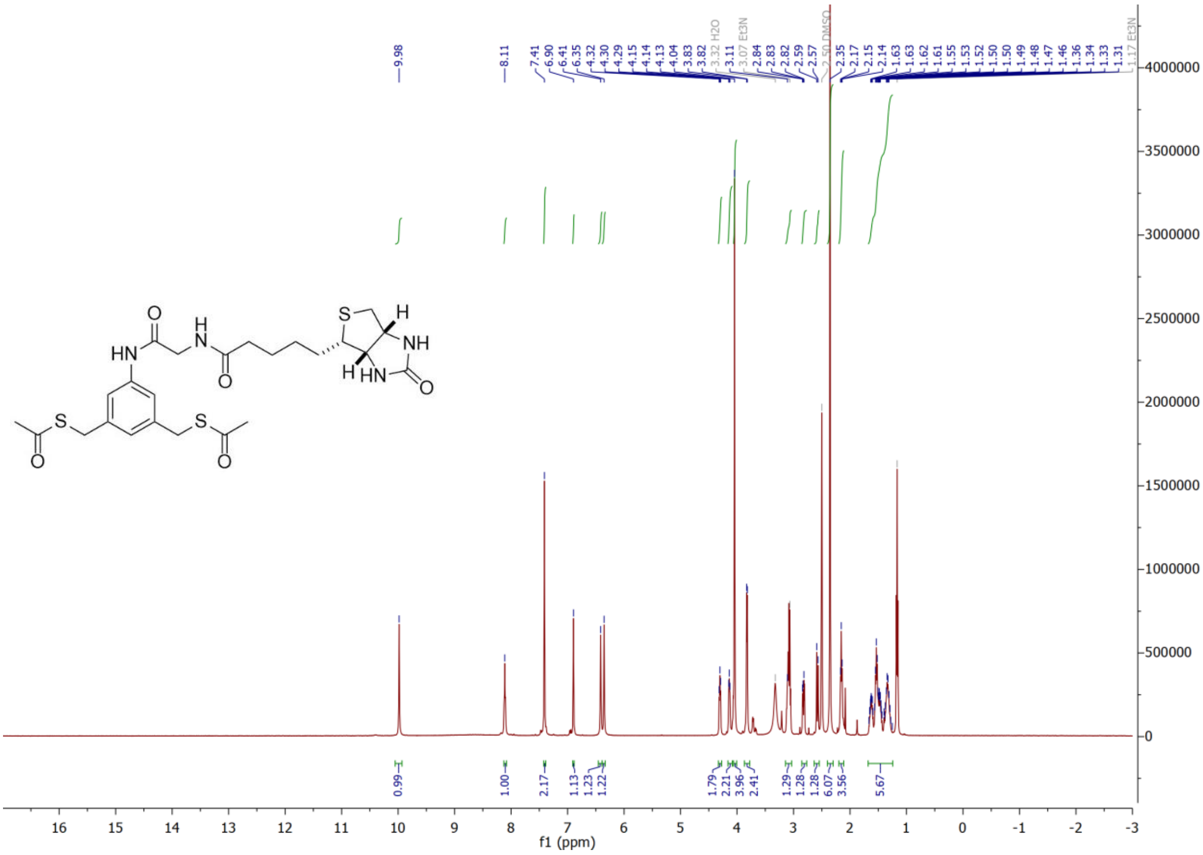


**Spectrum 10.** $^{1}$H NMR Spectrum of compound **4** in DMSO-$d_6$.

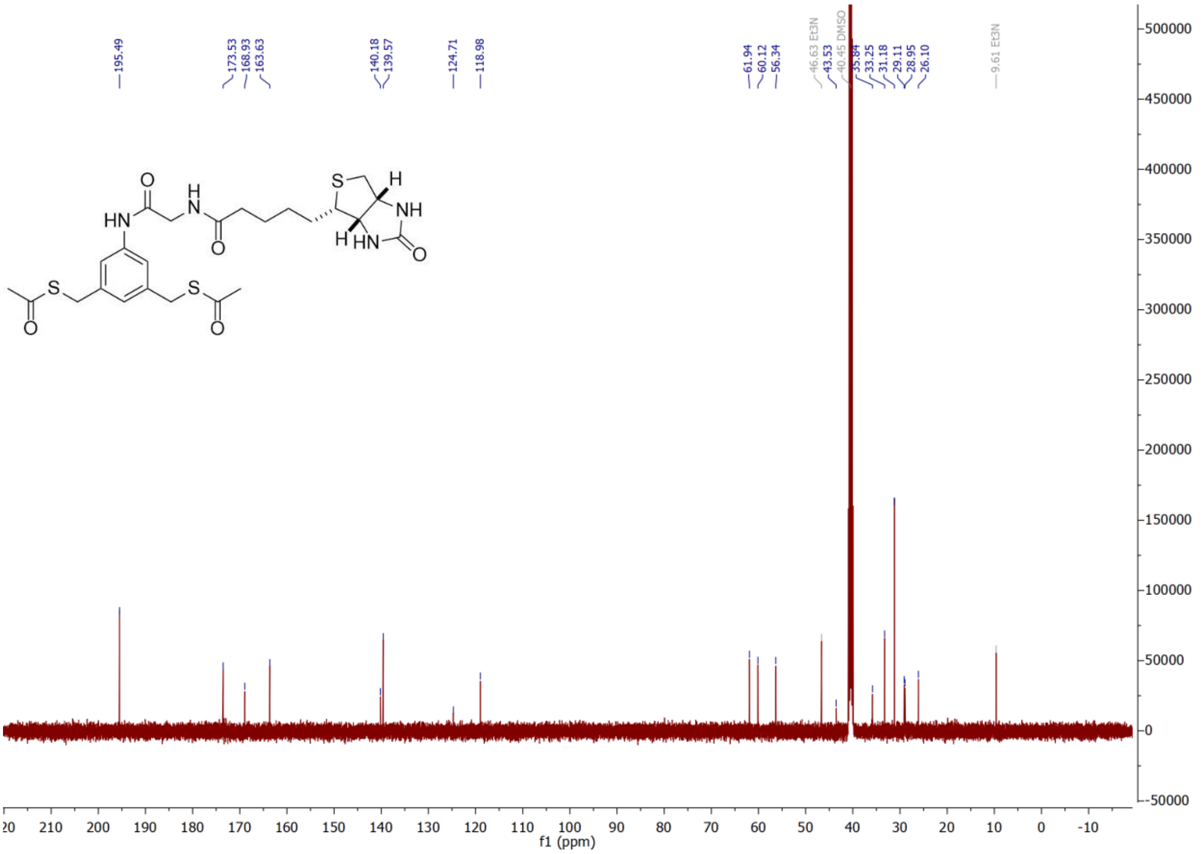


**Spectrum 11.** $^{13}$C NMR Spectrum of compound **4** in DMSO-$d_6$.

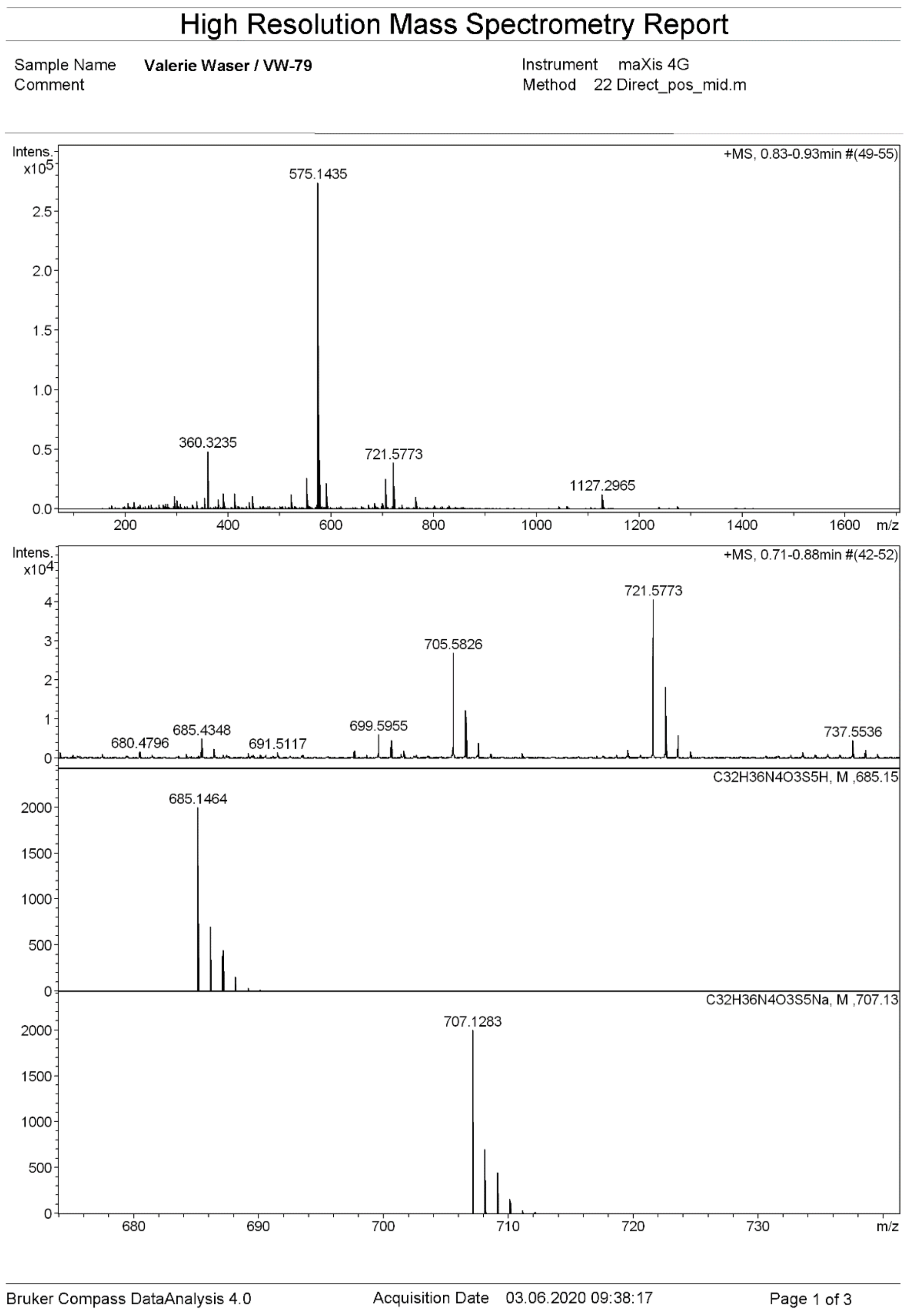


**Spectrum 12.** HRMS of compound **4**.

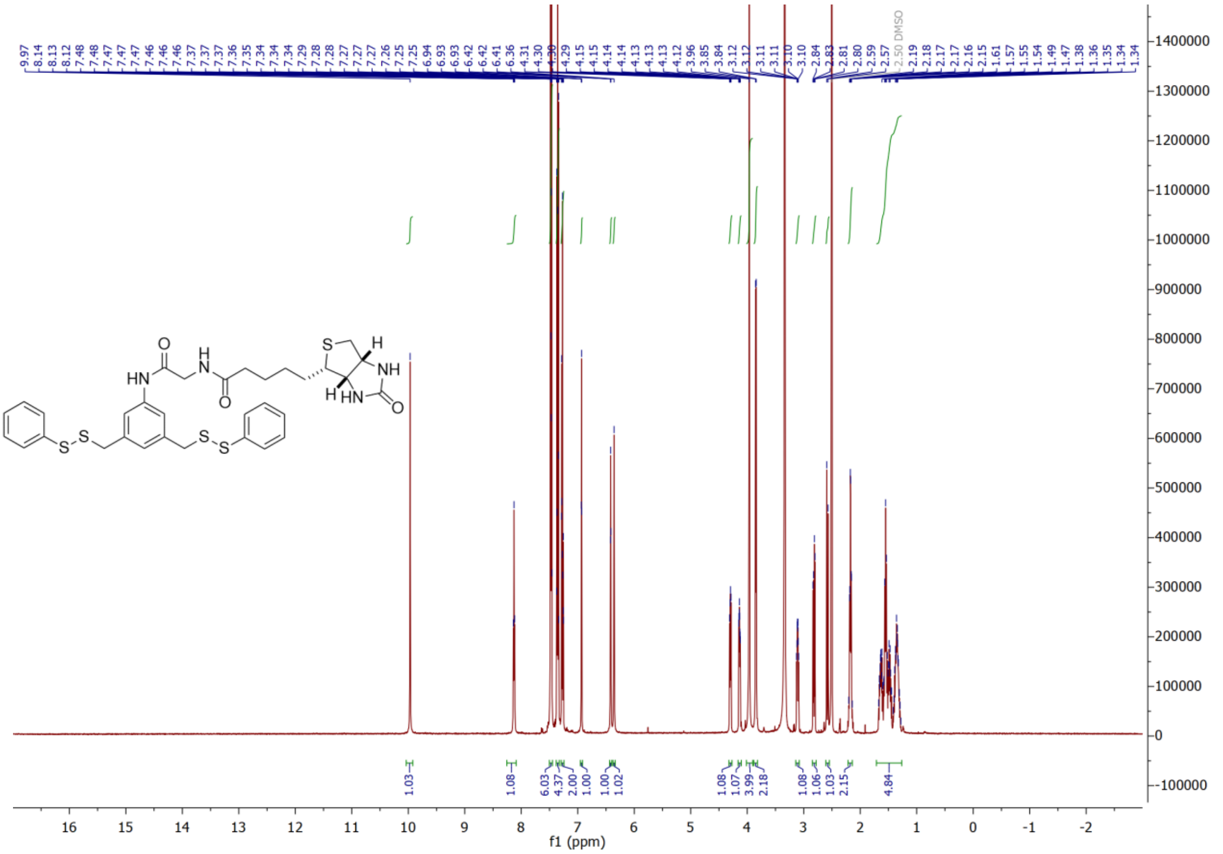

**Spectrum 13.** $^{1}$H NMR Spectrum of compound **5** in DMSO-$d_6$.

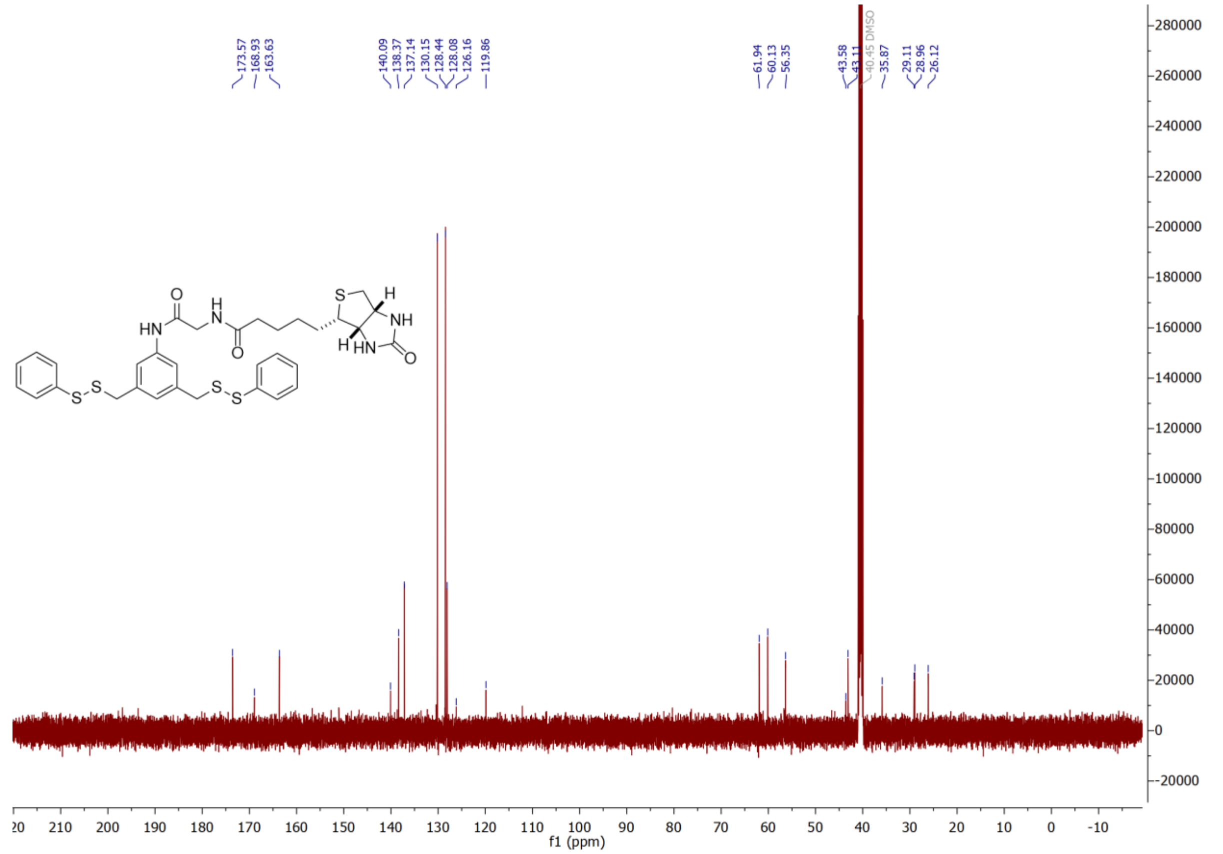

**Spectrum 14.** $^{13}$C NMR Spectrum of compound **5** in DMSO-$d_6$.

## High Resolution Mass Spectrometry Report

| | | | |
|---|---|---|---|
| Sample Name | **VW-296** | Instrument | maXis 4G |
| Comment | dissolved in DCM, MeOH added for analysis | Method | 24 Direct_pos_high.m |

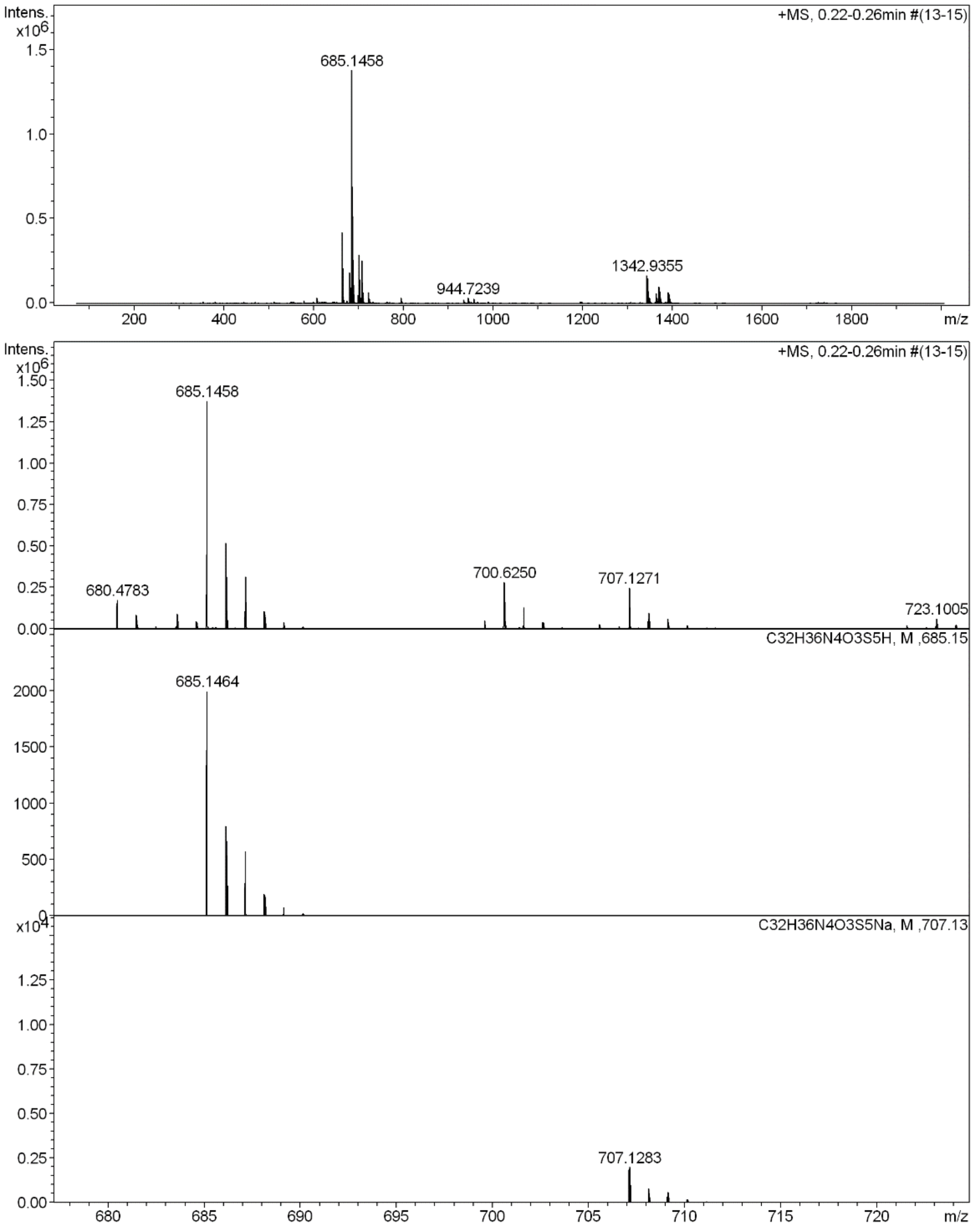


Bruker Compass DataAnalysis 4.0 Acquisition Date 21.12.2021 13:39:20 Page 1 of 3

**Spectrum 15.** HRMS of compound **5**.

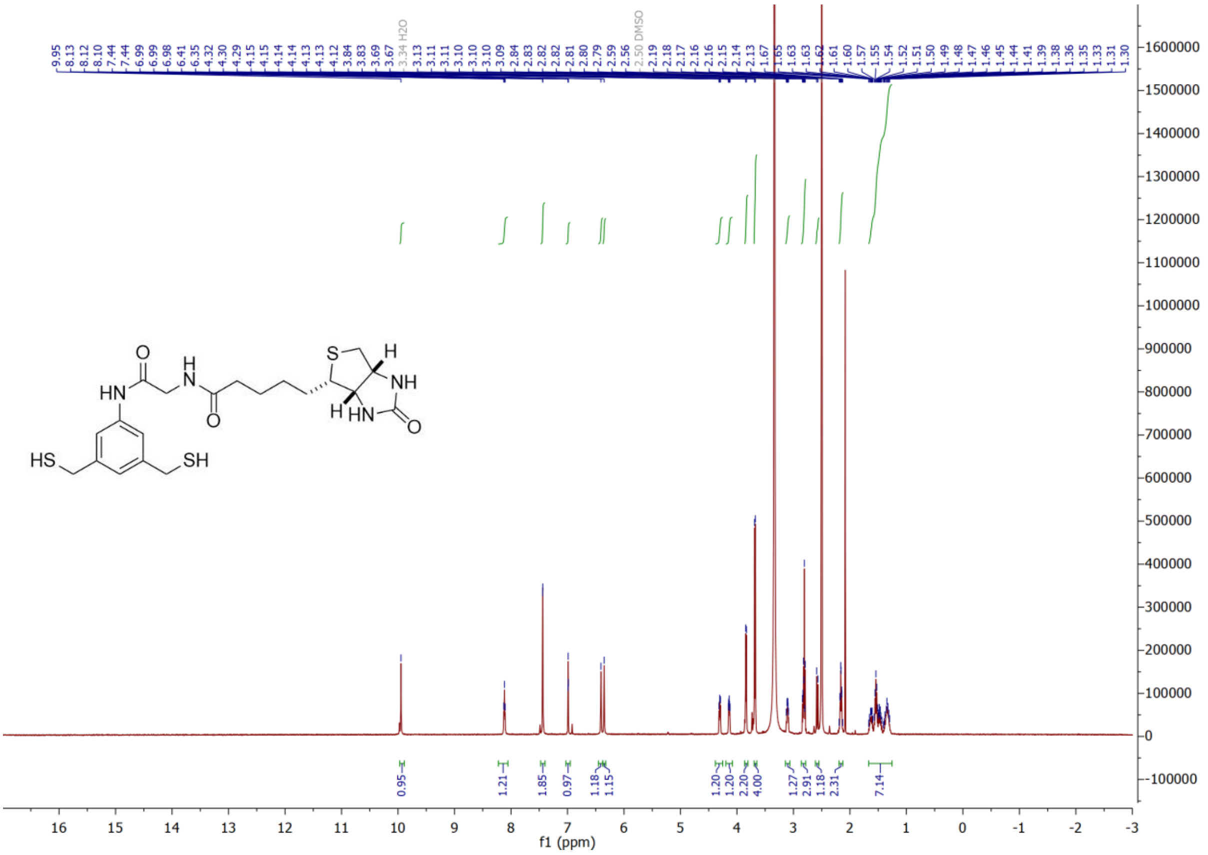

**Spectrum 16.** $^{1}$H NMR Spectrum of **(Biot-gly)** in DMSO-$d_6$.

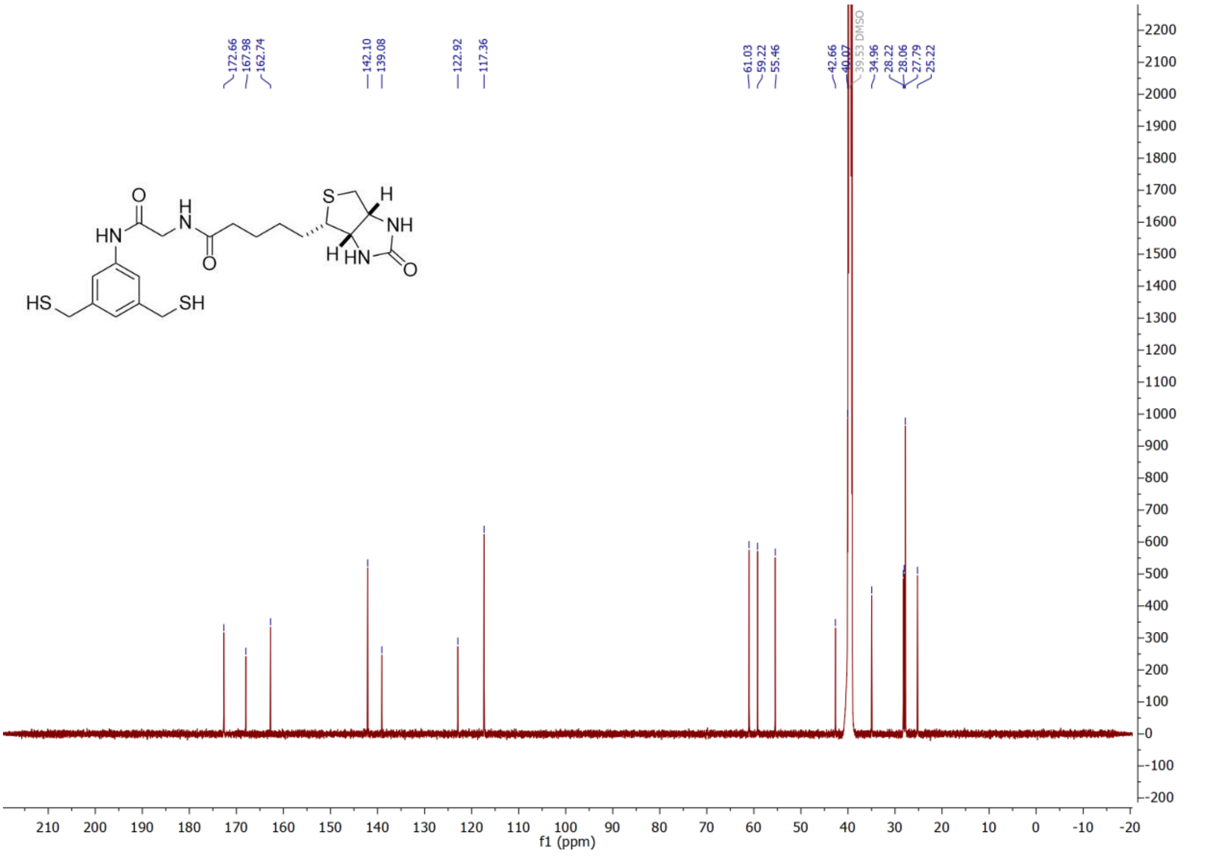

**Spectrum 17.** $^{13}$C NMR Spectrum of compound **(Biot-gly)** in DMSO-$d_6$.

## High Resolution Mass Spectrometry Report

| | | | |
|---|---|---|---|
| Sample Name | **VW-293** | Instrument | maXis II |
| Comment | | Method | ms_nocolumn_pos_mid.m |

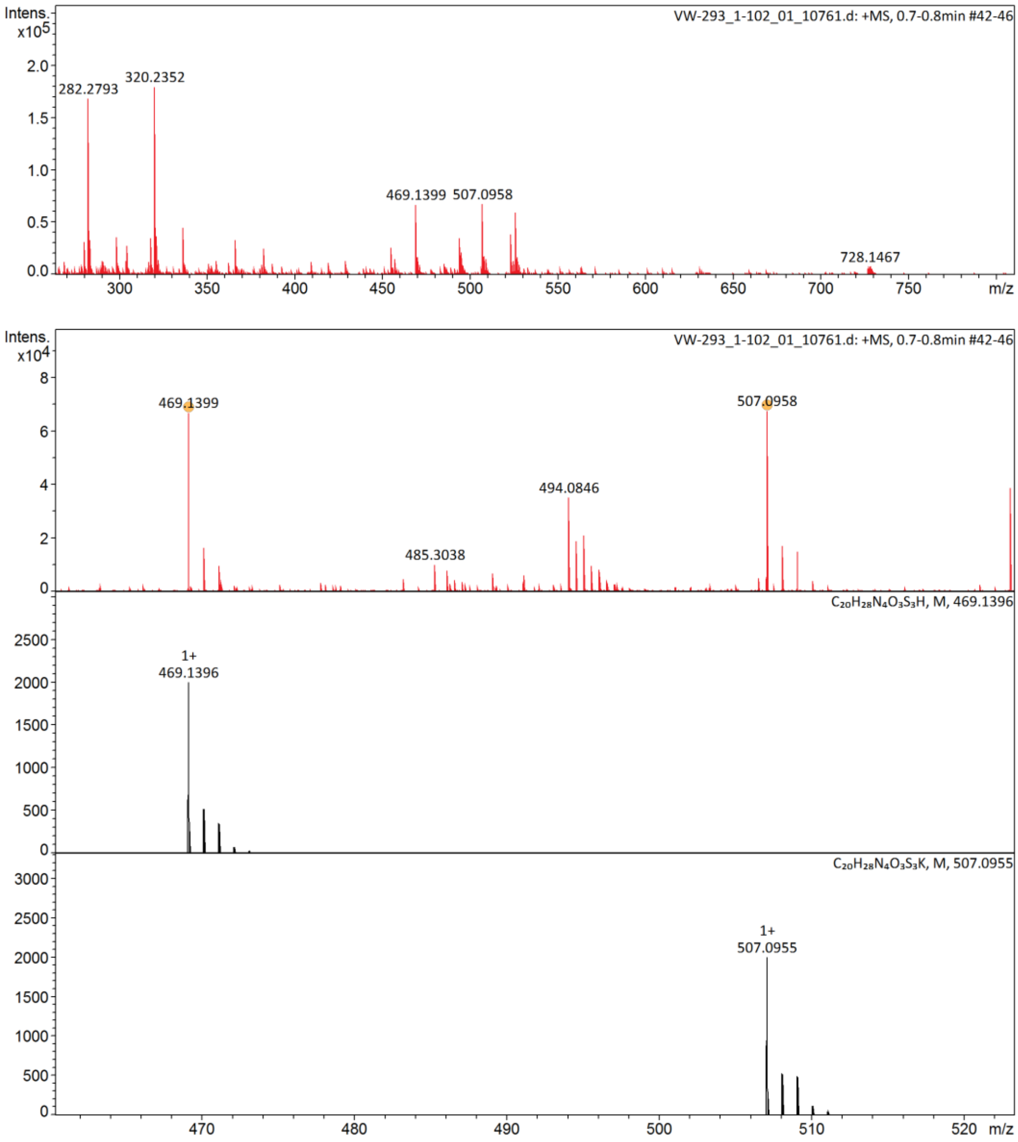


Bruker Compass DataAnalysis 4.4 Acquisition Date 9/21/2022 11:59:33 AM Page 1 of 3

**Spectrum 18.** HRMS of **(Biot-gly)**.

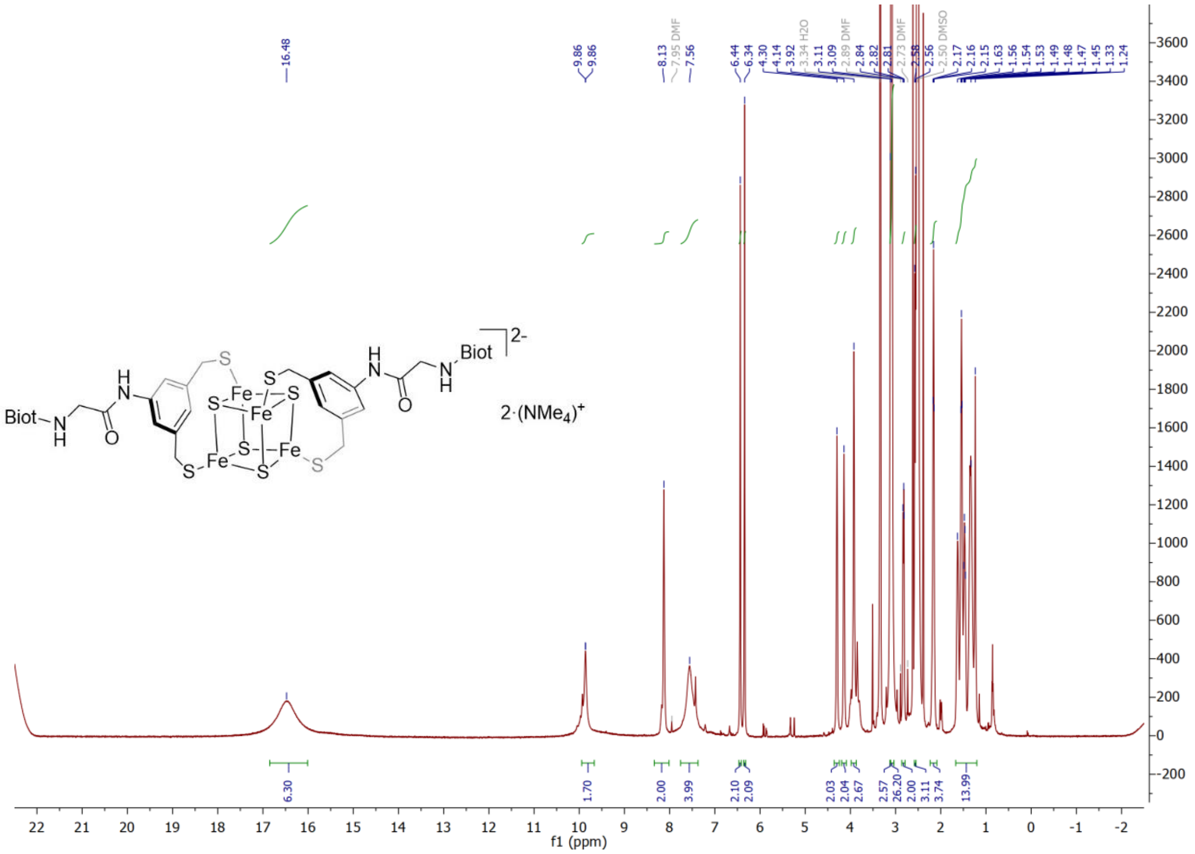

**Spectrum 19.** $^{1}$H NMR Spectrum of compound **[(Biot-gly)$_2$Fe$_4$S$_4$]** in DMSO-$d_6$.

# High Resolution Mass Spectrometry Report

| | | | |
|---|---|---|---|
| Sample Name | **Valérie Waser / VW-81** | Instrument | maXis 4G |
| Comment | | Method | 32 Direct_neg_mid.m |

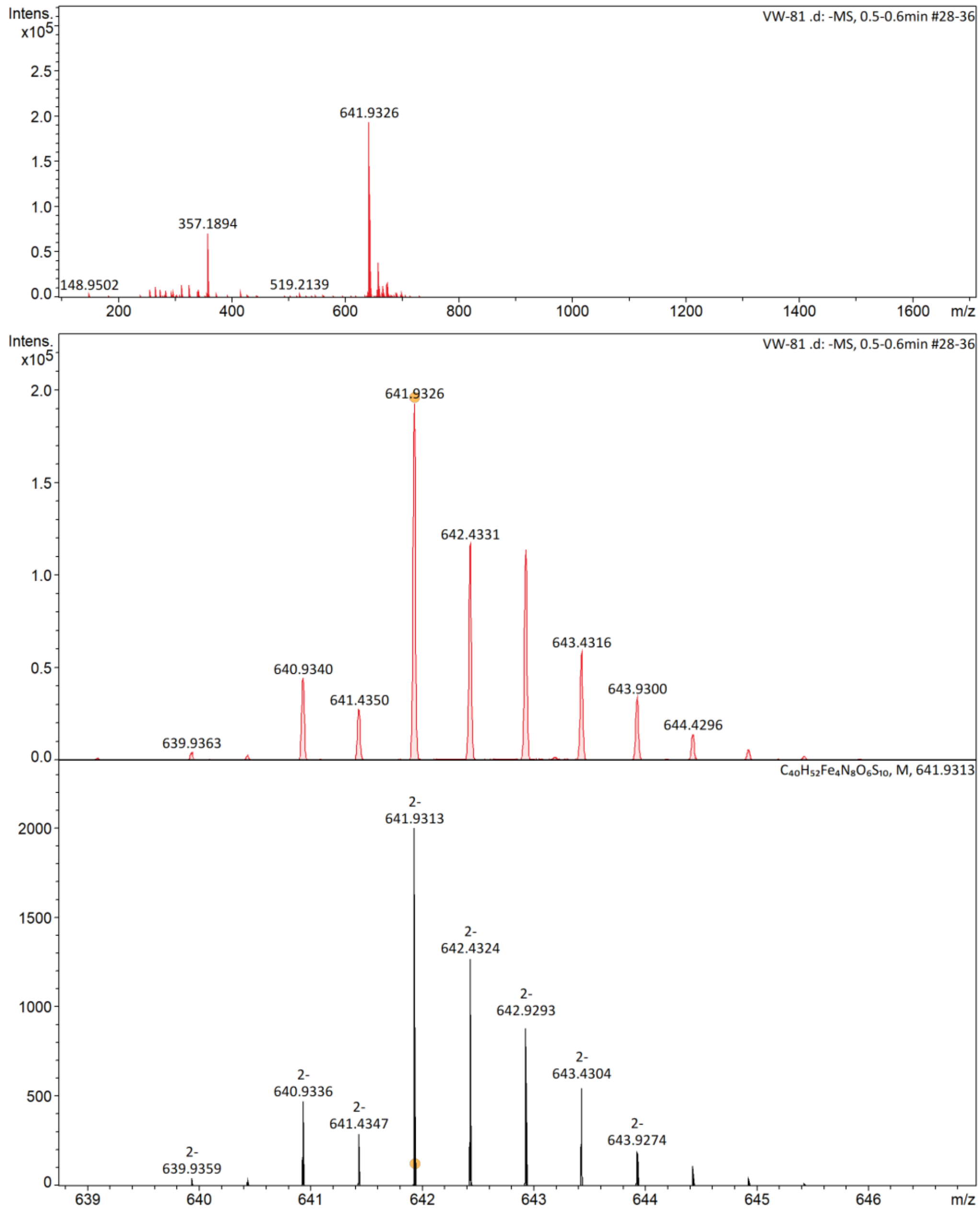


Bruker Compass DataAnalysis 4.4 Acquisition Date 19.06.2019 12:13:20 Page 1 of 3

**Spectrum 20.** HRMS of compound **[(Biot-gly)$_2$Fe$_4$S$_4$]**.

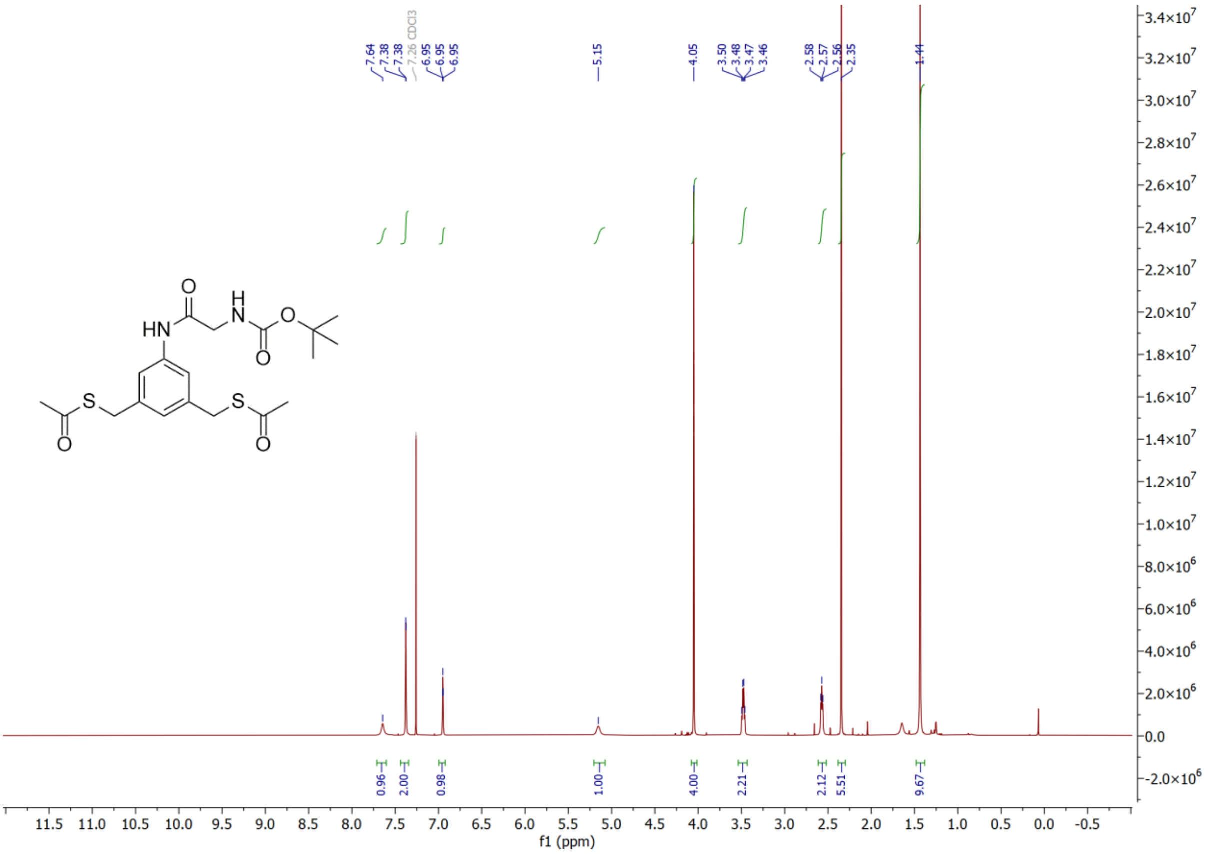

**Spectrum 21.** $^{1}$H NMR Spectrum of intermediate **4b’** in $CDCl_3$.

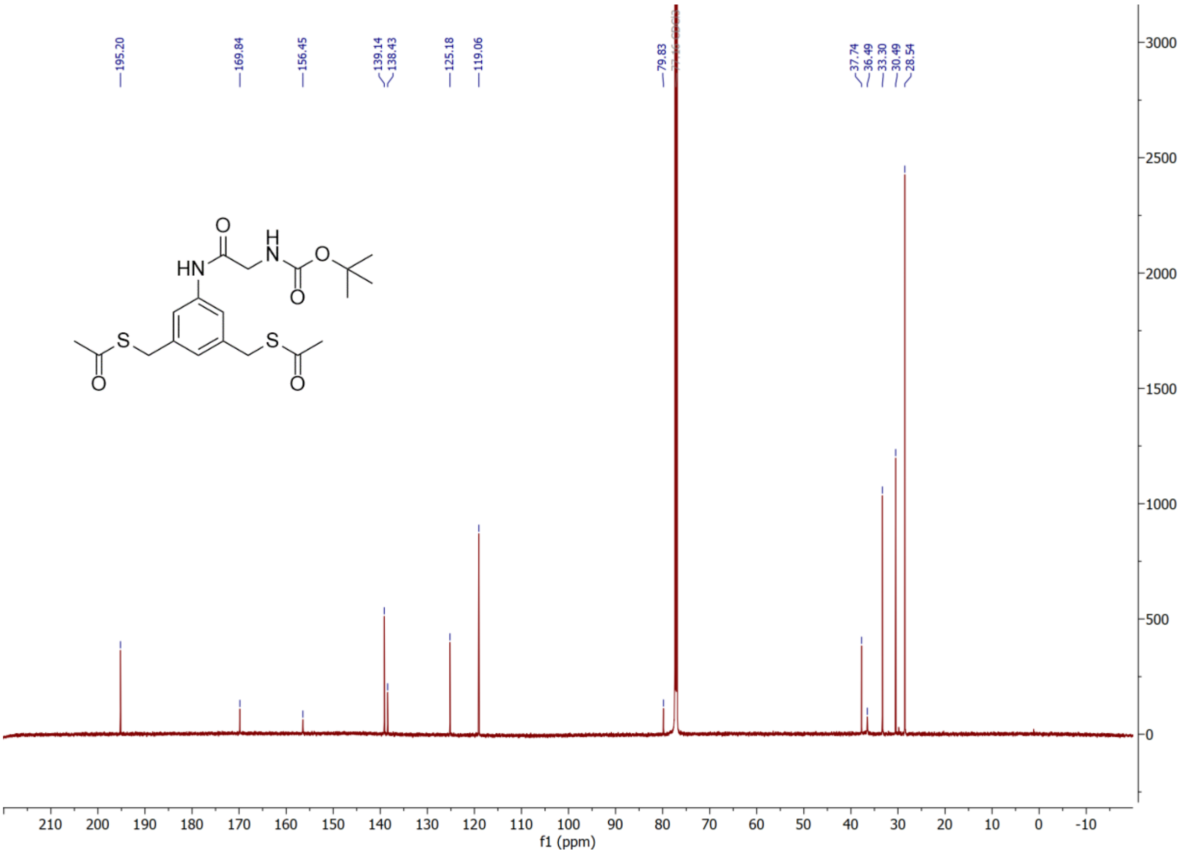

**Spectrum 22.** $^{13}$C NMR Spectrum of intermediate **4b’** in $CDCl_3$.

## High Resolution Mass Spectrometry Report

Sample Name **VW 220**
Comment

Instrument maXis 4G
Method ms_nocolumn_mid_pos.m

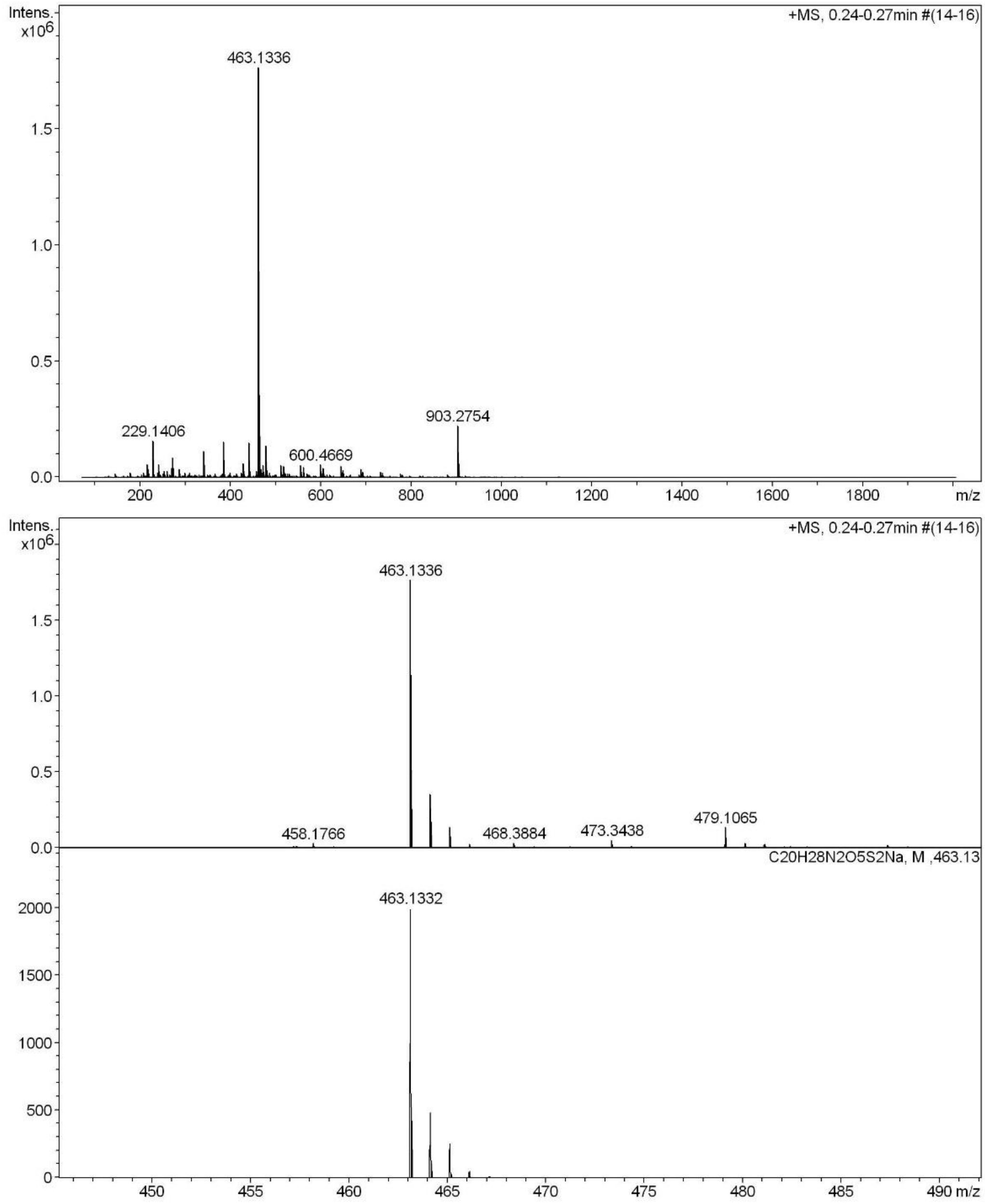


Bruker Compass DataAnalysis 4.0 Acquisition Date 10.06.2021 11:11:16 Page 1 of 3

**Spectrum 23.** HRMS of intermediate **4b'**.

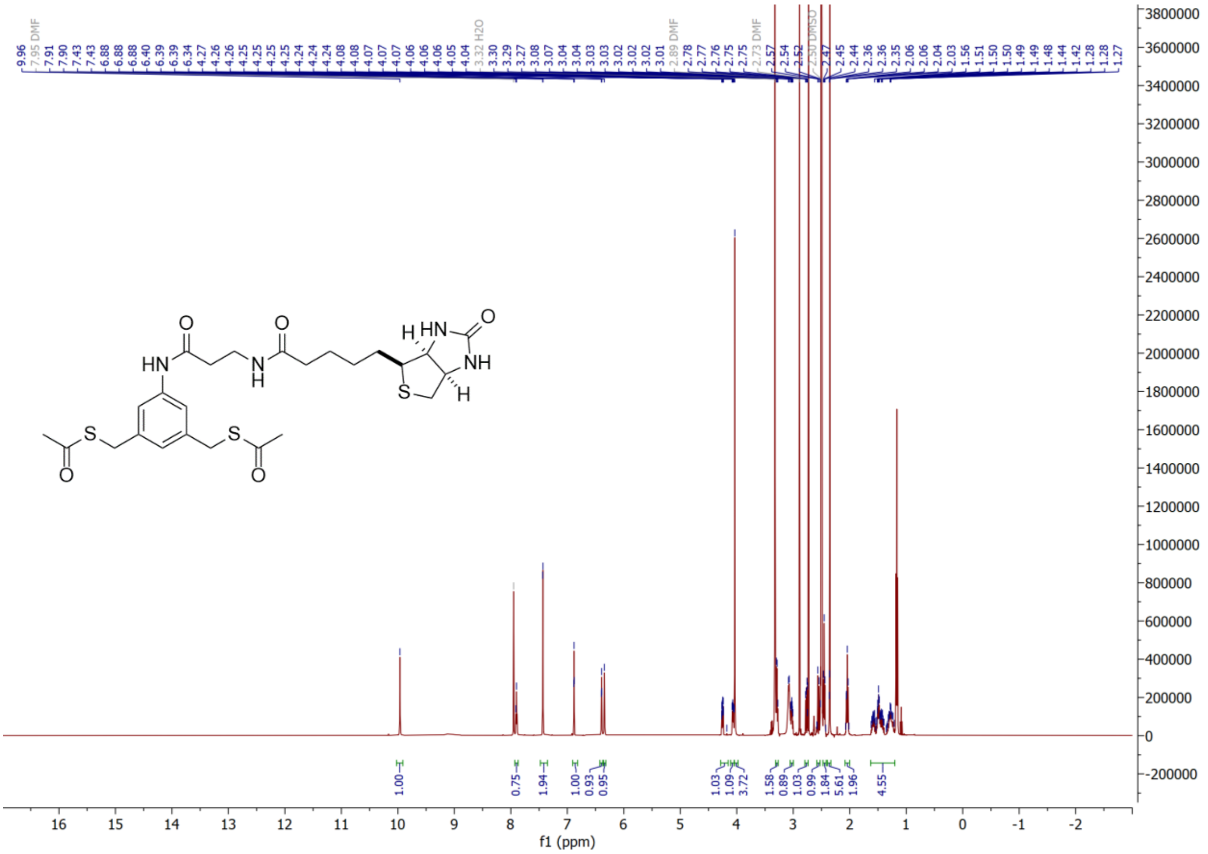


**Spectrum 24**. $^{1}$H NMR Spectrum of compound **4’** in DMSO-$d_6$.

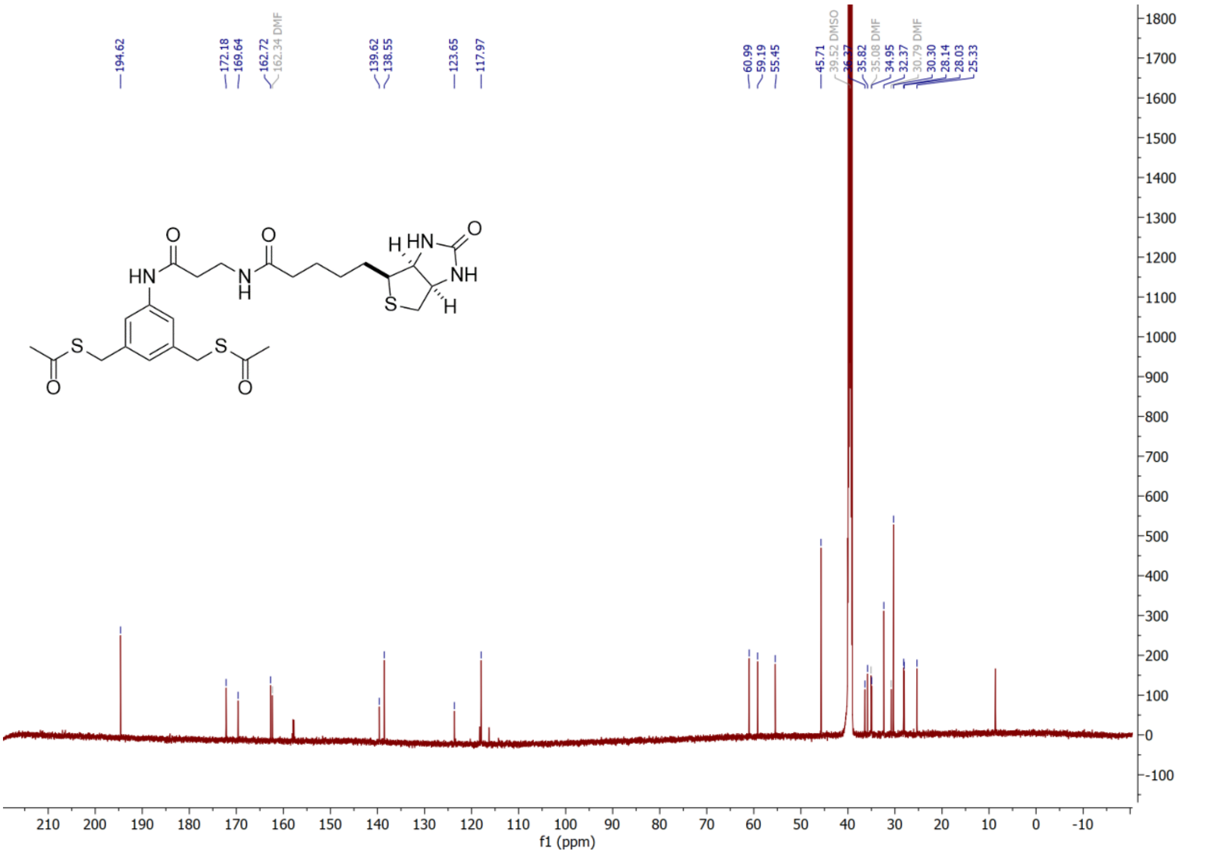


**Spectrum 25.** $^{13}$C NMR Spectrum of compound **4’** in DMSO-$d_6$.

# High Resolution Mass Spectrometry Report

Sample Name **VW 224**
Comment

Instrument maXis 4G
Method ms_nocolumn_mid_pos.m

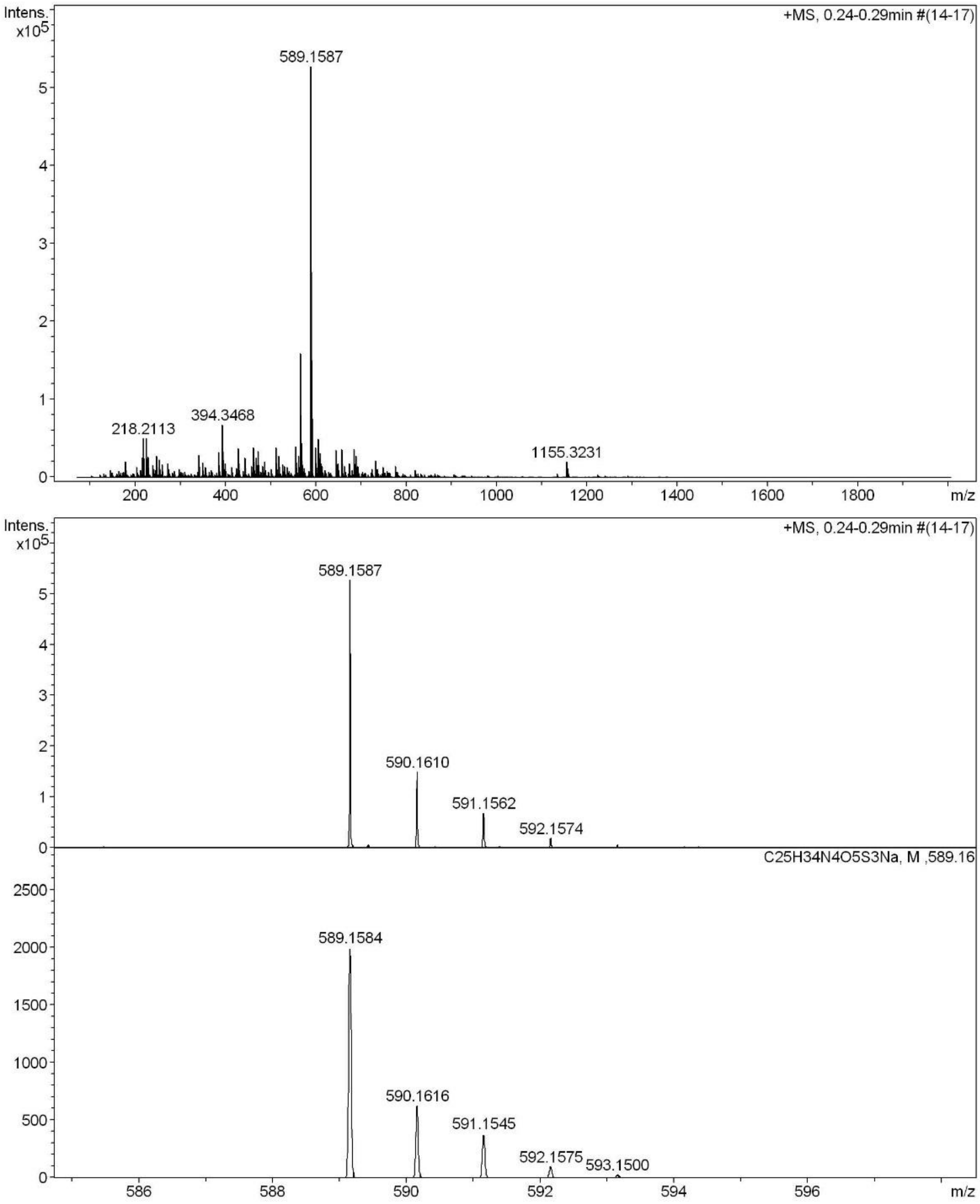


Bruker Compass DataAnalysis 4.0 Acquisition Date 10.06.2021 11:14:19 Page 1 of 3

**Spectrum 26.** HRMS of compound **4’**.

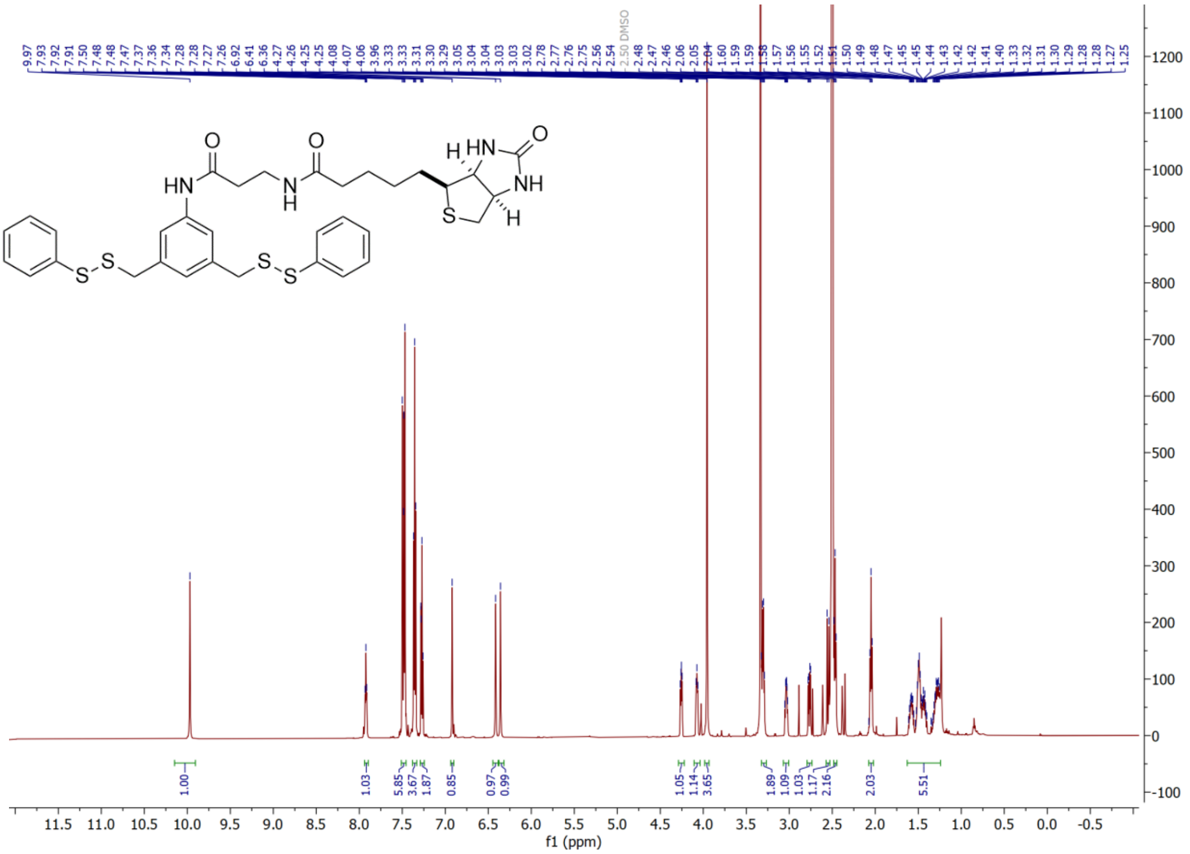

**Spectrum 27**. $^{1}$H NMR Spectrum of compound **5’** in DMSO-$d_6$.

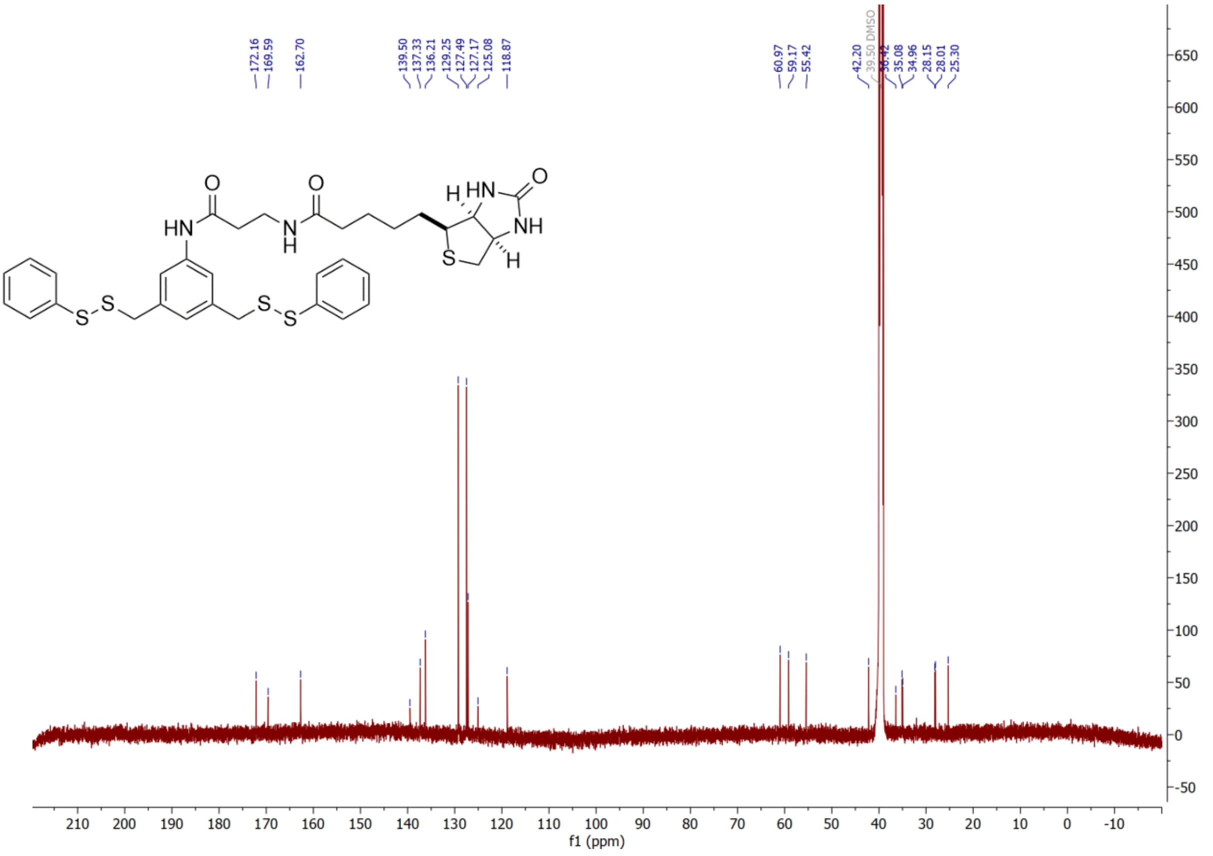

**Spectrum 28**. $^{13}$C NMR spectrum of compound **5’** in DMSO-$d_6$.

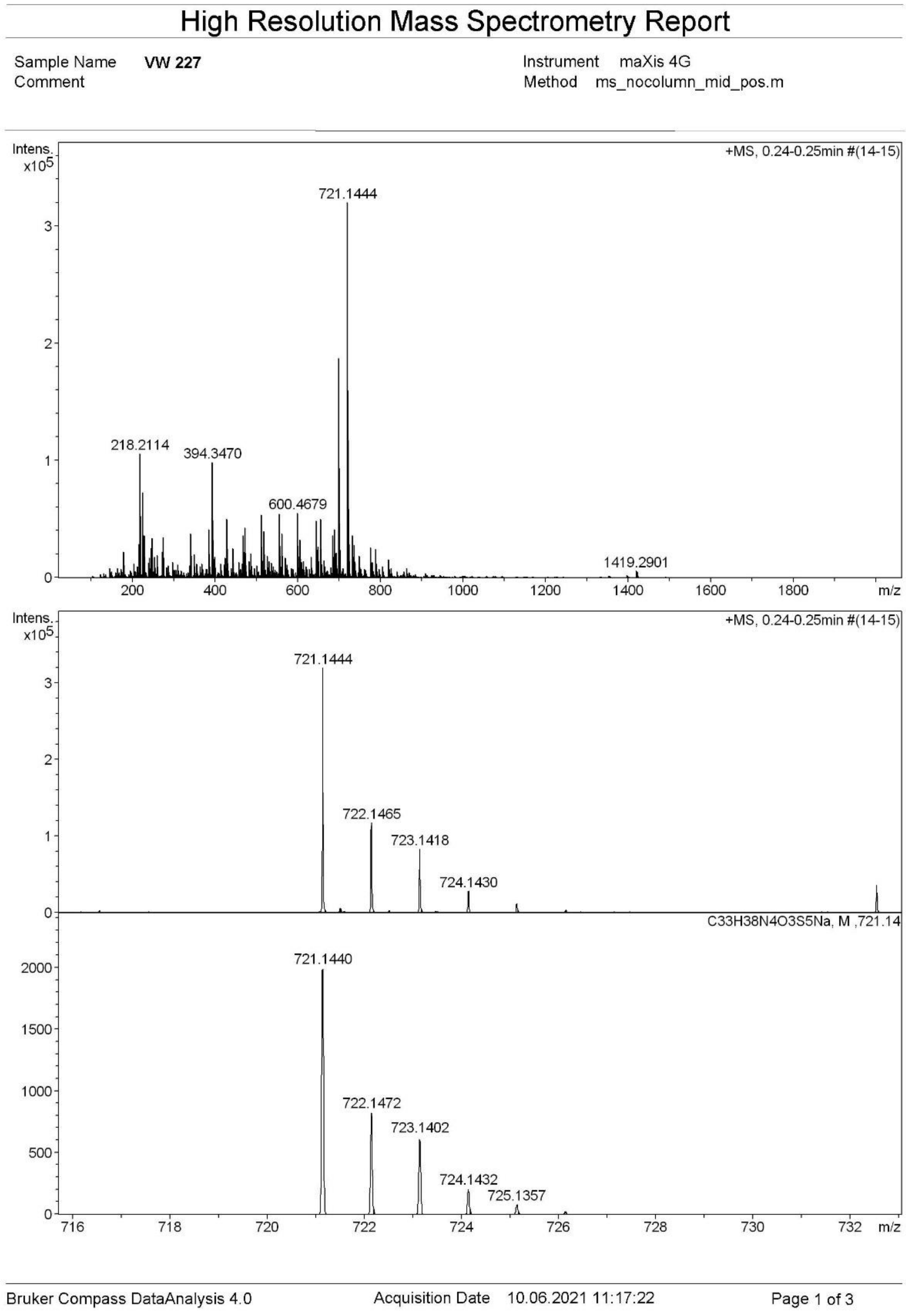


**Spectrum 29.** HRMS of compound compound **5’**.

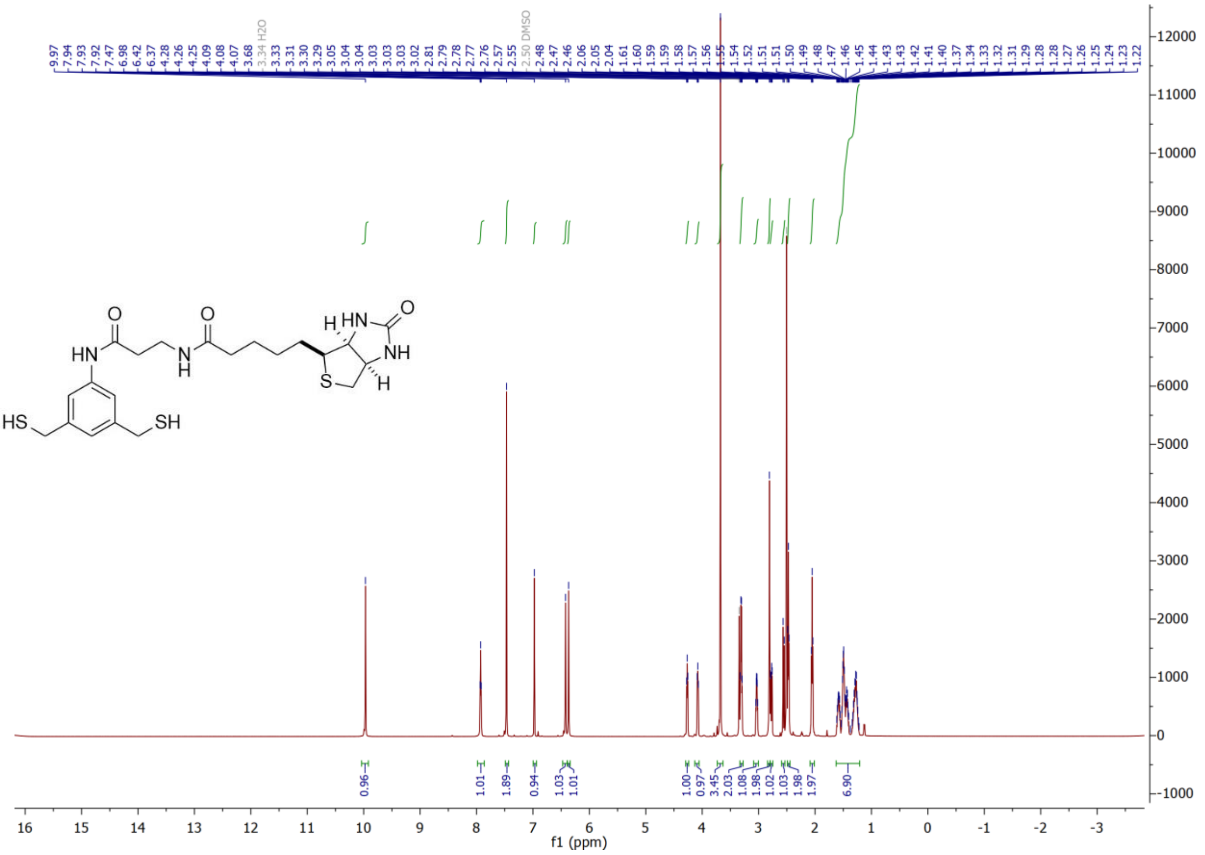

**Spectrum 30.** $^{1}$H NMR Spectrum of compound **(Biot-β-ala)** in DMSO-$d_6$.

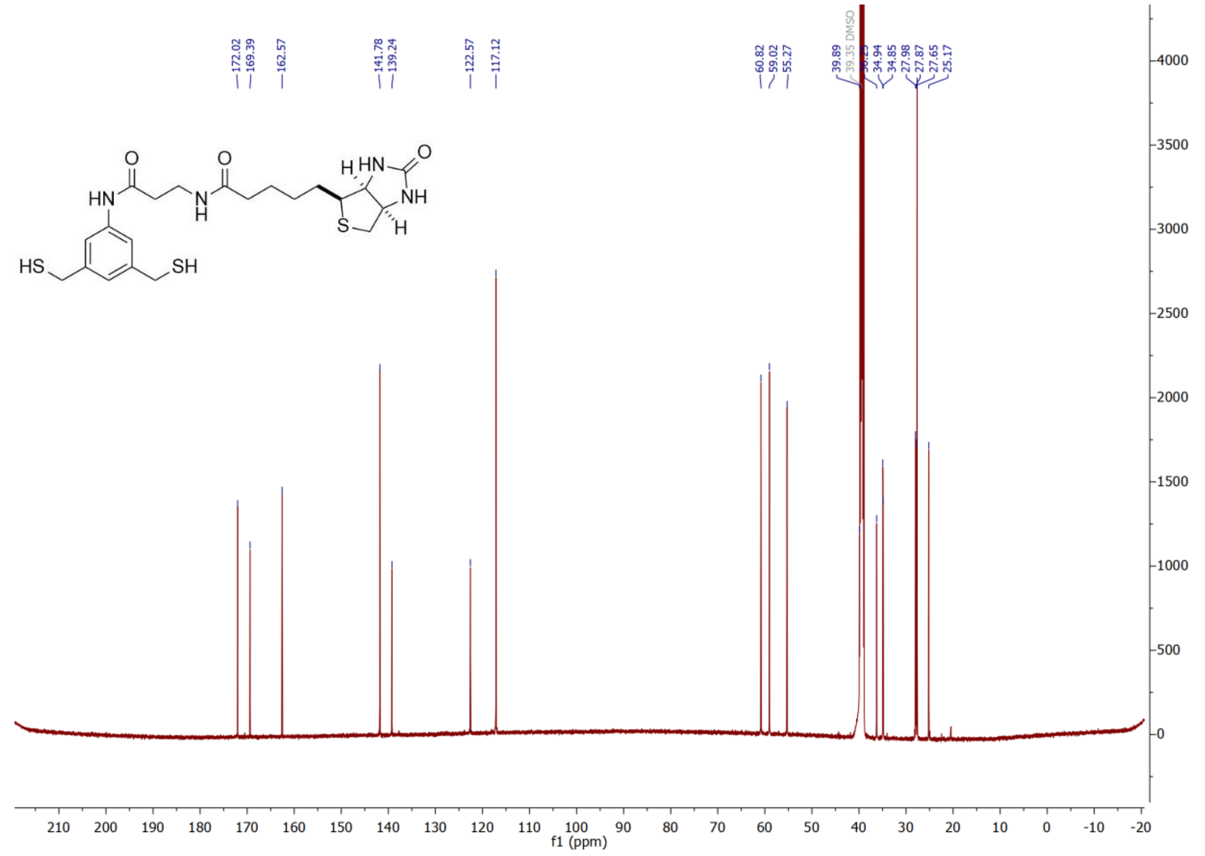

**Spectrum 31.** $^{13}$C NMR Spectrum of compound **(Biot-β-ala)** in DMSO-$d_6$.

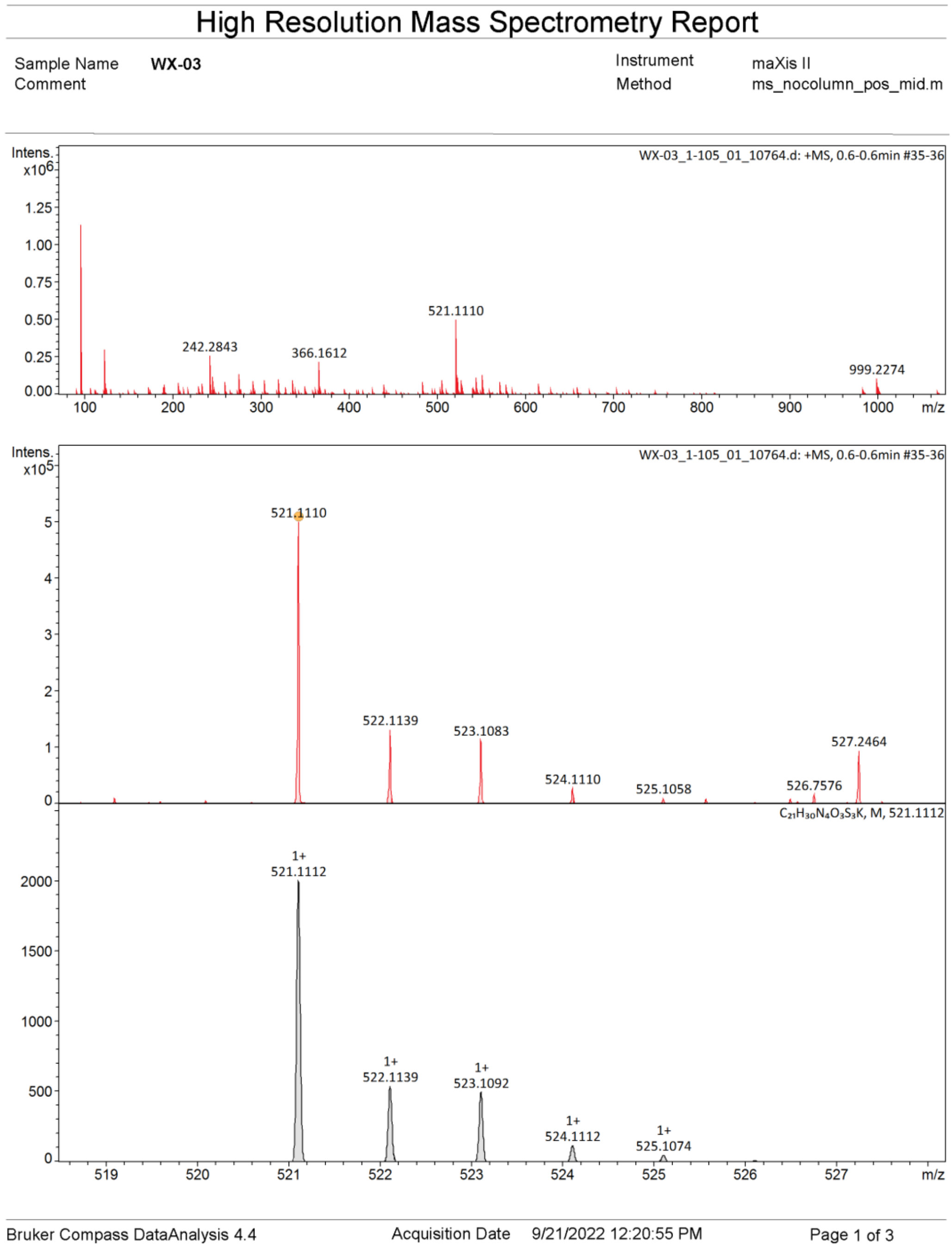


**Spectrum 32.** HRMS of **(Biot-β-ala)**.

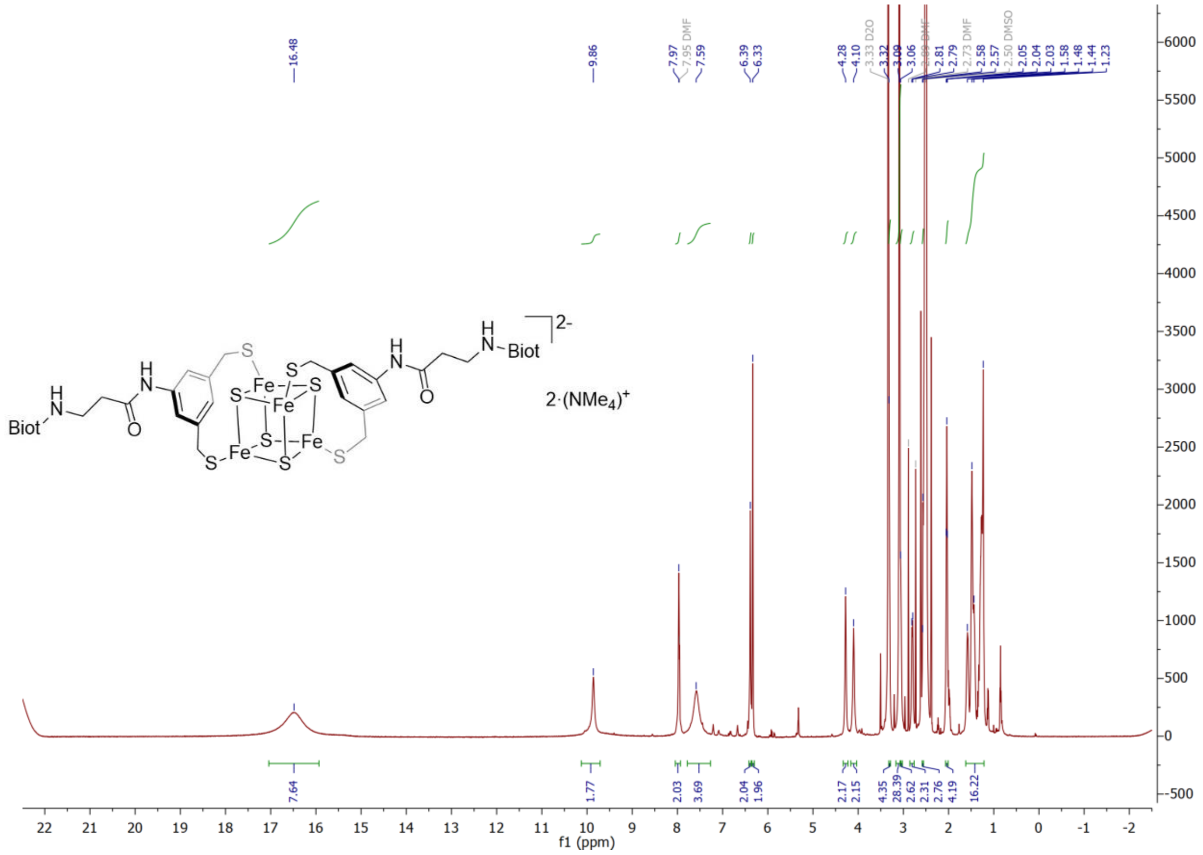

**Spectrum 33.** $^{1}$H NMR Spectrum of **[(Biot-β-ala)$_2$Fe$_4$S$_4$]** in DMSO-$d_6$.

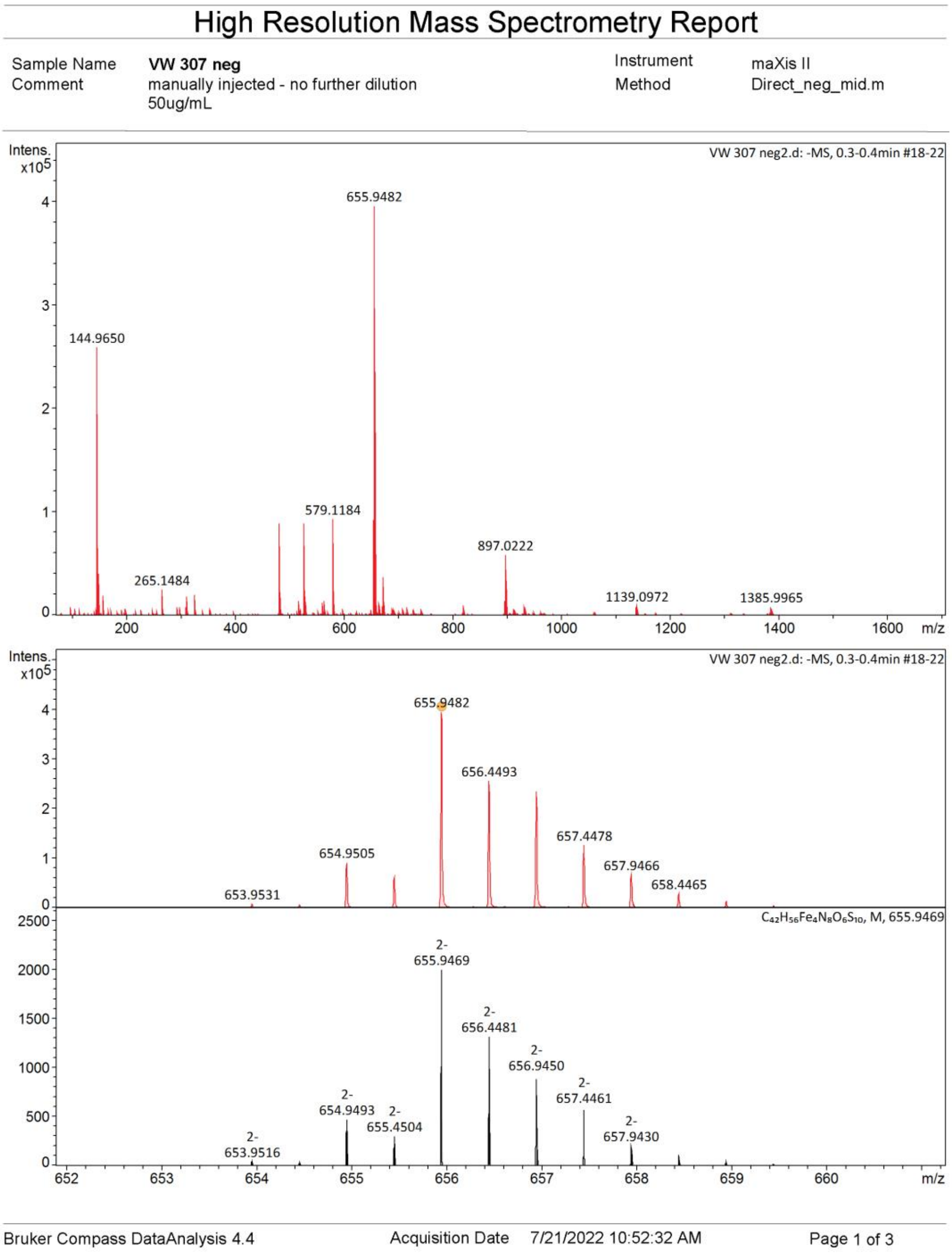


**Spectrum 34.** HRMS of **[(Biot-β-ala)$_2$Fe$_4$S$_4$]**.
